# Recent climate-driven ecological change across a continent as perceived through local ecological knowledge

**DOI:** 10.1371/journal.pone.0224625

**Published:** 2019-11-22

**Authors:** Suzanne M. Prober, Nat Raisbeck-Brown, Natasha B. Porter, Kristen J. Williams, Zoe Leviston, Fiona Dickson

**Affiliations:** 1 CSIRO Land and Water, Wembley, Western Australia, Australia; 2 CSIRO Land and Water, Canberra, Australian Capital Territory, Australia; 3 School of Arts and Humanities, Edith Cowan University, Joondalup, Western Australia, Australia; 4 Department of the Environment and Energy, Canberra, Australian Capital Territory, Australia; University of Waikato, NEW ZEALAND

## Abstract

Documenting effects of climate change is an important step towards designing mitigation and adaptation responses. Impacts of climate change on terrestrial biodiversity and ecosystems have been well-documented in the Northern Hemisphere, but long-term data to detect change in the Southern Hemisphere are limited, and some types of change are generally difficult to measure. Here we present a novel approach using local ecological knowledge to facilitate a continent-scale view of climate change impacts on terrestrial biodiversity and ecosystems that people have perceived in Australia. We sought local knowledge using a national web-based survey, targeting respondents with close links to the environment (e.g. farmers, ecologists), and using a custom-built mapping tool to ask respondents to describe and attribute recent changes they had observed within an area they knew well. Results drawn from 326 respondents showed that people are already perceiving simple and complex climate change impacts on hundreds of species and ecosystems across Australia, significantly extending the detail previously reported for the continent. While most perceived trends and attributions remain unsubstantiated, >35 reported anecdotes concurred with examples in the literature, and >20 were reported more than once. More generally, anecdotes were compatible with expectations from global climate change impact frameworks, including examples across the spectrum from organisms (e.g. increased mortality in >75 species), populations (e.g. changes in recruitment or abundance in >100 species, phenological change in >50 species), and species (e.g. >80 species newly arriving or disappearing), to communities and landscapes (e.g. >50 examples of altered ecological interactions). The overarching pattern indicated by the anecdotes suggests that people are more often noticing climate change losers (typically native species) than winners in their local areas, but with observations of potential ‘adaptation in action’ via compositional and phenological change and through arrivals and range shifts (particularly for native birds and exotic plants). A high proportion of climate change-related anecdotes also involved cumulative or interactive effects of land use. We conclude that targeted elicitation of local ecological knowledge about climate change impacts can provide a valuable complement to data-derived knowledge, substantially extending the volume of explicit examples and offering a foundation for further investigation.

## Introduction

Climate change is rapidly emerging as one of the most significant threats to biodiversity and ecosystems worldwide [1, 2]. Global temperatures now average nearly 1°C greater than in the pre-industrial era, and climate variability and extreme events are increasing [3, 4]. Researchers have already documented widespread climate-associated ecological change, including mass mortality events, shifting species distributions, altered phenology, and changing ecosystem productivity [2, 4–6], with subsequent effects on societies and economies predicted around the world [7].

Observing and documenting effects of climate change on biodiversity and ecosystems is an important step towards achieving effective responses through climate change mitigation and adaptation activity. In particular, understanding impacts can directly guide choice of adaptation options [8], as well as influence willingness to take action [9, 10]. While ecological effects of climate change have been widely documented for a broad range of taxonomic groups and ecosystem types, large geographic gaps in documentation remain [5]. Most empirical studies of ecological impact originate from northern Europe, North America and Russia, facilitated by the availability of long-term observational records. Data from the Southern Hemisphere, including Africa, South America and Australia, are limited [5, 11–13] despite disparities in projected changes in climate from those in the Northern Hemisphere [14].

Indeed, a synthetic understanding of recent climate change effects on the biota and ecosystems of the Australian continent is limited by lack of long-term accessible ecological data streams [2, 4, 5, 14–16]. The Intergovernmental Panel on Climate Change Australasia report [13] for example, suggests there is robust evidence only for distributional change (with all examples provided from marine environments) and phenological change [14, 17], with ‘limited agreement and evidence’ cited for vegetation change and disease, and no evidence provided for other types of change.

On the other hand, a growing number of observations and anecdotes about impacts of climate change on biodiversity and ecosystems is emerging in Australia (e.g. [2, 18]. The value of local ecological knowledge as a complement to scientific ecological knowledge is increasingly recognized [19, 20], but its systematic application to understanding climate change impacts remains limited [21]. Nevertheless, its potential value for climate change science is slowly being recognized [9, 21, 22], for example a number of studies found that most anecdotal records of climate change were supported by or complementary to climatic records [9, 21, 22]. Further, Pyhälä, Fernandez-Llamazares [21] argue that people’s perceptions are a ‘key ingredient in the design, planning, and implementation of successful adaptation strategies’. When interpreted with sufficient caution, such observations and anecdotes are thus of potential value for providing an indicative overview of potential climate change impacts before sufficient numerical data become available [9, 21]. For example, they can point to potential organisms, ecosystems and locations for future research focus, provide alternative lines of evidence for data or models [20, 23], and be used as illustrations or case examples in communicating potential consequences of climate change to broader audiences.

Towards these goals, we present a novel approach that uses local ecological knowledge to facilitate a continent-scale view of climate change impacts on terrestrial biodiversity and ecosystems that people have already perceived in Australia. Our study aimed to elicit and synthesise anecdotal information about recent ecological change in Australia, drawing on a respondent group with close links to Australian environments. To maximize the continental perspective, we sought input to a web-based survey from such people across Australia who had been familiar with a location (that they selected) for at least 10–20 years, with the aid of a custom-built survey mapping tool. We used the resulting spatial, numerical and textual information to collate an overview of perceptions of climate-related ecological change in Australia. Of particular interest was the potential of the method to complement data-derived knowledge, by eliciting information on the following themes: (a) the frequency and spatial location of different types of change perceived by respondents, (b) details about the species, ecosystems and ecological processes involved in perceived changes, (c) the perceived importance of interactions among land use, climate and other drivers of change, and (d) the prevalence of perceived ecological cascades and other complex changes.

## Methods

### Procedure

We conducted a national online survey about perceptions of recent ecological change and the potential drivers of these changes using Qualtrics software (October 2016, Qualtrics, Provo, UT, USA). All research was undertaken according to protocols approved by the CSIRO Human Ethics Committee (ID 094/16). Two pilot surveys involving 35 respondents participating in one or both pilots (including nine farmers, nine natural resource managers, 14 ecological researchers and three government agency staff) were undertaken to refine the survey questions.

The refined survey (S1 Appendix) was open from June-December 2017. We actively promoted participation from people with close links to the Australian environment by contacting 300 known experts (farmers, ecologists) and over 300 organizations that represent farmers, graziers, bee keepers, ecological researchers, natural resource managers, naturalists and others with interests in environmental management and ecological change in Australia. We contacted each organization up to three times by email or phone, and requested our survey information and link be distributed via their networks and newsletters. We also advertised in the Ecological Society of Australia National Conference Handbook 2017, and contributed to occasional extension articles and radio interviews. This resulted in 326 survey responses.

### Measures

All questions in the survey were optional. The survey began with a custom-designed mapping tool that we embedded in Qualtrics, allowing respondents to outline on a map of Australia a terrestrial location (of any size) that they had ‘known well for at least 10–20 years’ (hereafter ‘selected area’). All subsequent questions about ecological change pertained to this selected area.

The remainder of the survey involved three parts. In Part 1, we asked multiple-choice questions about ten types of ecological change respondents may have observed in their selected area within the last 10–20 years (hereafter ‘primary change types’ and associated ‘primary questions’, Table 1). The primary change types captured readily observable impacts of environmental and land management change, reflecting key elements of the climate change ecological impact framework of Bellard, Bertelsmeier [1] and Scheffers, De Meester [4] (adapted for this study in Table 1). The impact framework recognizes levels of change grading from organisms (mortality, disease) and populations (phenology, recruitment and abundance) to species (distributions), communities, ecosystems and landscapes (although questions were not asked in this order).

**Table 1:** Framework used for classifying impacts of climate change on biodiversity from organism to landscape scales [1, 4].

Scale	Impact	#	Primary questions #1–10 asked in Part 1 of survey Over the last 10–20 years have you seen:
1. Organisms	*Genetics*	10	Other changes?
	*Morphology*	10	Other changes?
	*Physiology*		
	Mortality	1	Plants dying more than you’d normally expect?
		2	Animals dying more than you’d normally expect?
	Pests and diseases	3	Unusually higher or lower levels of pests or diseases in plants or animals?
2. Populations	*Phenology*	4	Plants flowering, fruiting, germinating or having growth flushes at unusual times of year?
		5	Birds, butterflies, other insects or migrating animals appearing earlier or later than you would normally expect? (or other timing changes)
	*Dynamics*		
	Age structure	10	Other changes?
	Sex ratios	10	Other changes?
	Recruitment	6	Unusually high or low levels of shrubs or trees establishing (e.g. woody thickening or lack of tree recruitment)?
	Abundance	10	Other changes (in plant abundance)?
		7	Unusual increases or decreases in the abundance of animals?
3. Species	*Distribution*	8	Species disappearing from the area?
		9	Different species arriving in the area?
4. Communities	*Interspecific relationships*	10	Other changes/cascades?
to biomes	*Ecosystem productivity*	10	Other changes?
	*Ecosystem structure/composition*	10	Other changes?
5. Landscapes	*Landscape processes*	10	Other changes?

#Primary question or change type from Part 1 of the survey

For any of the ten primary change types, we asked the respondents whether they had observed the relevant change in their selected area, and if so, whether they felt that any of a suite of land use, climate or other drivers had contributed to that change (multiple drivers could be selected; S1 Appendix). Where a climate driver was selected, further information was requested regarding whether respondents felt the climate driver was part of a normal climate cycle, unprecedented or related to climate change. Participants were provided opportunities to elaborate on any of these changes, and the tenth question allowed respondents to report changes that were not captured in the prior questions.

In Part 2, respondents were asked to provide a free-text anecdote describing one or more of the observations of recent ecological change in their selected area that they had indicated in Part 1, and the potential drivers for these changes. Anecdotes reflecting changes that the participants felt may be due to climate change were encouraged (in Part 2 only), to maximize capture of climate change-related anecdotes. Details of potential drivers, and species and ecological communities involved, were requested in conjunction with these anecdotes.

Part 3 sought demographic information about the respondents, and their views about environmental change. To gauge views about climate change of respondents in our sample compared with the views of the general public, we asked a question that had been included in a previous survey of the general Australian public [24]. This asked respondents to indicate whether they think climate change is happening, and if so whether they believe humans are causing it (hereafter ‘climate change belief’). We also included a question about perceptions of the Australian environment generally (hereafter ‘environmental belief’, scored as (1) generally improving, (2) staying much the same, and (3) generally worsening.

### Data analysis

We analysed responses to Part 1 multiple-choice questions using summary statistics, graphs and spatial maps. To gauge the influence of respondent demographics on responses, we applied binomial generalized linear models with the log link function, using Genstat 16.0. We used the total number of the ten primary change types that respondents indicated they had observed (for any drivers, or for land use, climate or climate change drivers only) as response variables, with the total number of primary questions answered by each respondent as binomial totals (to account for differential survey effort). We tested the response variables against each demographic variable individually, then used all subsets regression to identify the best combined model (accepting the models with highest adjusted R^2^ in which all explanatory variables were significant).

We systematically analysed text responses by considering text responses from Parts 1 and 2 together, partitioning these into independent anecdotes (hereafter ‘anecdotes’), and scoring each of these anecdotes against a fixed set of criteria (Table 2). To qualify as an anecdote for the purposes of this study, we required that it describe a biological or ecological change (rather than a purely physical change such as increased stream erosion), and that the respondent had suggested at least one potential driver of this change.

**Table 2:** Information recorded for systematic partitioning and scoring of anecdotal information from the survey.

Information recorded
Description of biotic change or linked set of changes (e.g. cascade)
Description of reported driver(s) of biotic change(s)
Primary change types for which the anecdote provides an example (1–10, Table 1)
Attribution to climate change drivers (1, likely; 2, possible; 3, climate only; 4, no attribution)^1^
Attribution to land use/management drivers (1, likely; 2, possible; 3, no attribution; 4, restoration)^2^
Attribution to biotic invasion drivers (1, likely; 2, no attribution) ^3^
Types of organisms observed to undergo change (and species names where available)
Organisms affected native or exotic (1, native only; 2, exotic only; 3, both; 4, unknown) ^4^
Ecosystem type
Whether the anecdote includes a cascade

^**1**^**Attribution to climate change:** Indicates our interpretation of whether the respondent considered an ecological change to have arisen at least in part due to a climate change-related driver. This was scored as ‘likely’ if the respondent provided a clear text attribution to climate change (e.g. warming or drying conditions; sometimes with a statement of uncertainty). It was scored as ‘possible’ if the attribution to climate change was uncertain. The latter most commonly involved attribution to the Millenium drought, which was part of the most sustained drying trend in southern Australia since records began in 1900, and was linked to higher mean sea level pressure in southern Australia, a known response to global warming [25]. A ‘possible’ score was also attributed if climate change drivers were implied (but not explicitly stated) through the nature of the ecological change (typically a phenological change). If climate drivers were described without links to climate change (e.g. frosts, floods or droughts), the anecdote was attributed to ‘climate only’ drivers; otherwise they were scored as having ‘no attribution to climate change’. All attributions represent consensus between at least two researchers.

^2^**Attribution to land use drivers:** Indicates our interpretation of whether the respondent considered an ecological change to have arisen at least in part due to a land use or land management driver, e.g. overgrazing, urbanization or control burning regimes. This was scored as ‘likely’ if the respondent provided a clear text attribution to a land use or land management driver that was not an ecological restoration activity (sometimes including a statement of uncertainty). Where a clear statement was made that active ecological restoration management had contributed to the change, it was scored as ‘restoration’. Otherwise, the anecdote was scored as having ‘no attribution to land use drivers’.

^3^**Attribution to biological invasion:** Indicates our interpretation of whether the respondent considered an ecological change to have arisen at least in part due to a biological invasion (e.g. mortality of reptiles due to cane toad invasion). This was scored as ‘likely’ if the respondent provided a clear text attribution to a biotic invasion driver (sometimes including a statement of uncertainty). Otherwise, the anecdote was scored as having ‘no attribution to biological invasion drivers’.

^4^**Species origin.** Given expected movements of species under climate change, the term ‘native’ is used in this study to refer to species known to occur within Australia prior to the Industrial era (pre-1750). Local and non-local native species are used to delimit finer origin details where appropriate. 'Exotic species' is used in this study to refer to species with recent (post-Industrial era) origins outside of Australia. 'Planted' includes garden, amenity or cultivated plants.

Scoring included assessment of whether respondents suggested that climate change, land use, and/or biological invasions had contributed to the observed change, including allowance for some level of uncertainty (e.g. a climate change attribution was scored as possible if the attribution involved the Millenium drought, which was part of the most sustained drying trend in southern Australia since records began in 1900 [25]. Methods for assigning attribution are described in more detail in Table 2. A land use driver was defined as a management input associated with anthropogenic use of land (e.g. clearing, livestock grazing, or prescribed burning) or its consequences (e.g. fragmentation, spray drift). Anecdotes involving ecological cascades (i.e. one ecological change resulting in at least one other ecological change) or multiple linked pathways (e.g. related outcomes of the same drivers, such as suites of birds appearing and others disappearing due to drying conditions) were scored as single (complex) anecdotes in order to maintain these links.

Initial partitioning and scoring of all responses was undertaken by a single researcher (SMP) for maximum consistency, then treatment of each response was checked by a second researcher (FD or KJW). Disagreements occurred for about 21% of cases and were generally minor. They were resolved by discussion until consensus was reached, and scoring rules were refined if required. The most common point of contention involved whether to aggregate or partition an anecdote that involved several outcomes from the same or similar reported drivers. As noted above, we aimed to aggregate these, but the aggregated anecdote was still scored to capture the range of primary change types that were articulated (e.g. one anecdote could be linked to both plant mortality and animal decline).

We used anecdote scores as tools to facilitate synthesis of results, in particular for enumerating and summarising anecdotes representing each of the ten primary change types, to provide an overview of ecosystems and organisms involved, and to identify ecological cascades and interactions among land use, climate and other drivers of ecological change. We utilised the reference list from [26], and undertook general and specific literature searches using Web of Science to compare anecdotes with climate change impacts on Australian terrestrial biodiversity and ecosystems reported in the literature. Taxonomy follows the Australian Plant Census [27] and the Australian Faunal Directory [28] unless otherwise cited, with Latin names provided by respondents (updated where appropriate) or derived from common names where unambiguous. Common names only are provided where generic and species names could not be determined. We include quotes from respondents in our synthesis to help to convey the character of responses.

## Results

### Characteristics of survey respondents

There were 326 survey respondents, with 263‒326 responding to questions about each primary change type (average 8.76 of 10 primary questions answered per respondent). The following summary of respondent characteristics is drawn from the pool of 224 of the 326 respondents who answered at least some of the demographic questions (in Part 3).

Our sample included mostly people who believed climate change is happening (95.5%) and felt Australia’s environment is ‘generally worsening’ (82.9%). The former is a somewhat larger proportion than documented in surveys of the general public (84.5%,[24]), and included substantially more respondents who felt that humans are largely causing climate change (86%) when compared with the general population [24], (S2 Appendix: Figure A). There was participation from a wide diversity of occupations or interests related to the environment (S2 Appendix: Figure B), with the highest representation from respondents who considered themselves naturalists or observers of nature, bird watchers, ecological researchers, farmers, graziers, and members of Land Care or Catchment Management groups. More respondents were male (63.5%) than female (36.0%), and 2.1% of respondents identified as Indigenous. Respondents were mostly highly educated, with 45.9% having completed a post-graduate qualification, and another 30.5% having an undergraduate degree as their highest qualification. Household incomes were relatively evenly spread among income brackets (S2 Appendix: Table A).

**Fig 1:**
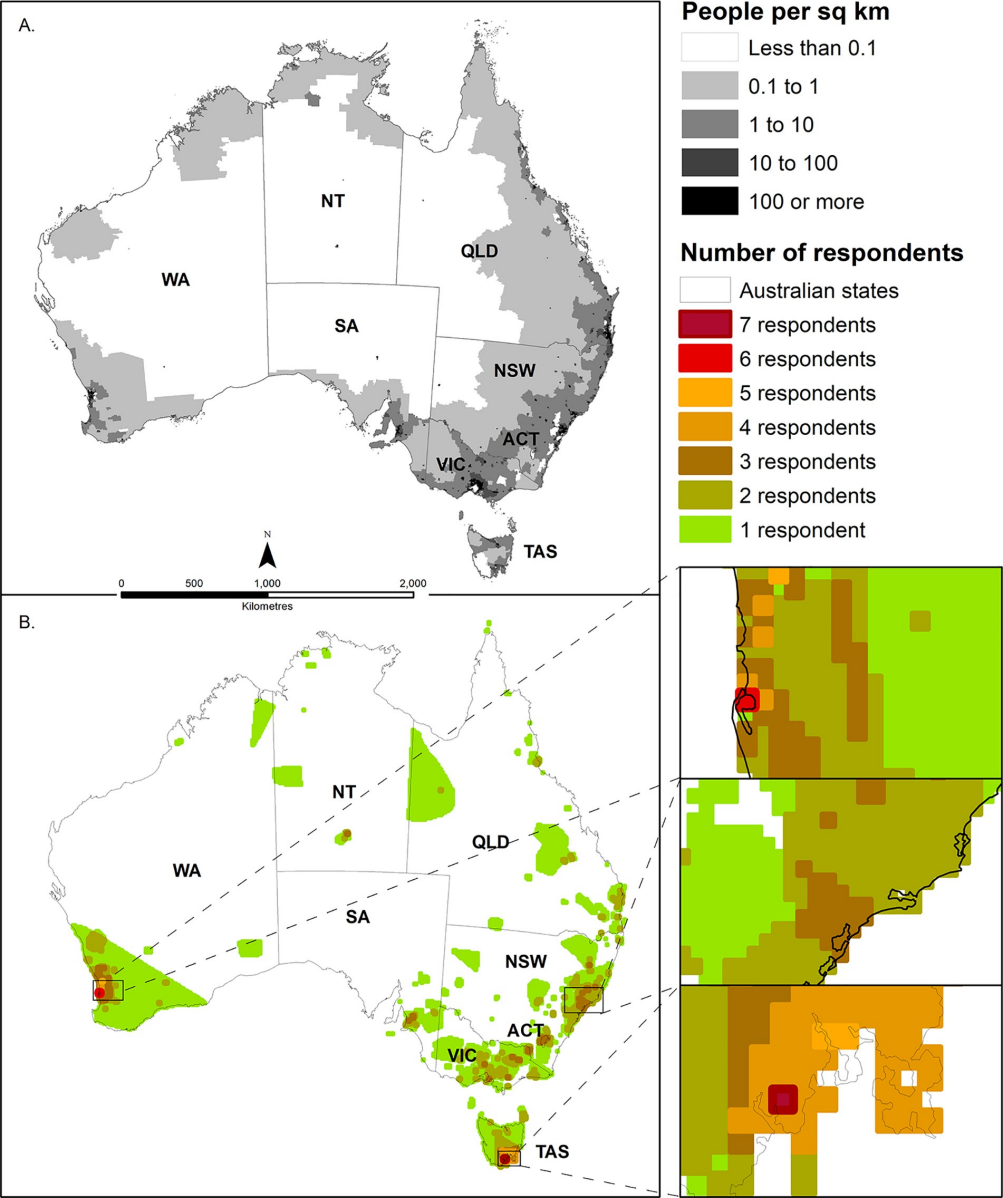
Distribution of respondent’s selected areas compared with Australian population densities. (a) Population density in Australia, 2016 [29], with state boundaries for Western Australia (WA), Northern Territory (NT), South Australia (SA), Queensland (QLD), New South Wales (NSW), Australian Capital Territory (ACT), Victoria (VIC) and Tasmania (TAS), (b) a heat map showing the distribution and concentration of respondents’ selected areas.

Geographically, approximately 10% of Australia was covered by at least one participant in their selected areas, with a bias towards south-eastern and south-western Australia, reflecting the distribution of the Australian population [29] (Fig 1). Nevertheless, numerous respondents selected areas in northern or remote Australia, with a notable absence being the mid-latitudes of Western Australia. Largely consistent with the distribution of selected areas, most respondents lived in New South Wales, Queensland or Victoria, with the fewest from the Northern Territory, Australian Capital Territory and Tasmania (S2 Appendix: Table B).

### Types and drivers of change observed by Part 1 respondents

Part 1 multiple-choice questions in our survey indicated a remarkably high degree of perceived ecological change in the parts of Australia reported on by participants, with respondents indicating they had perceived recent ecological changes associated with an average of 4.1 (range 0–10) or 41% of the ten primary change types in their selected areas. Notably, respondents attributed nearly two thirds of these perceived ecological changes to a potential climate change-related driver (with respondents perceiving an average of 2.7 (range 0–10) or 27% of primary change types out of a potential ten per respondent). This compared with a similar proportion attributed to land use drivers (averaging 2.4 (range 0–8) or 30% of eight potential primary change types per respondent). An average of 1.5 (range 0–8) or 19% of primary change types out of a potential eight per respondent were attributed to a land use plus a climate-related driver, suggesting climate change impacts are often compounded by land use factors. Only 3% of respondents indicated zero observations of change in their selected area, and 27% reported no potentially climate change-related observations. Twenty-four percent of respondents reported no land use drivers.

**Fig 2:**
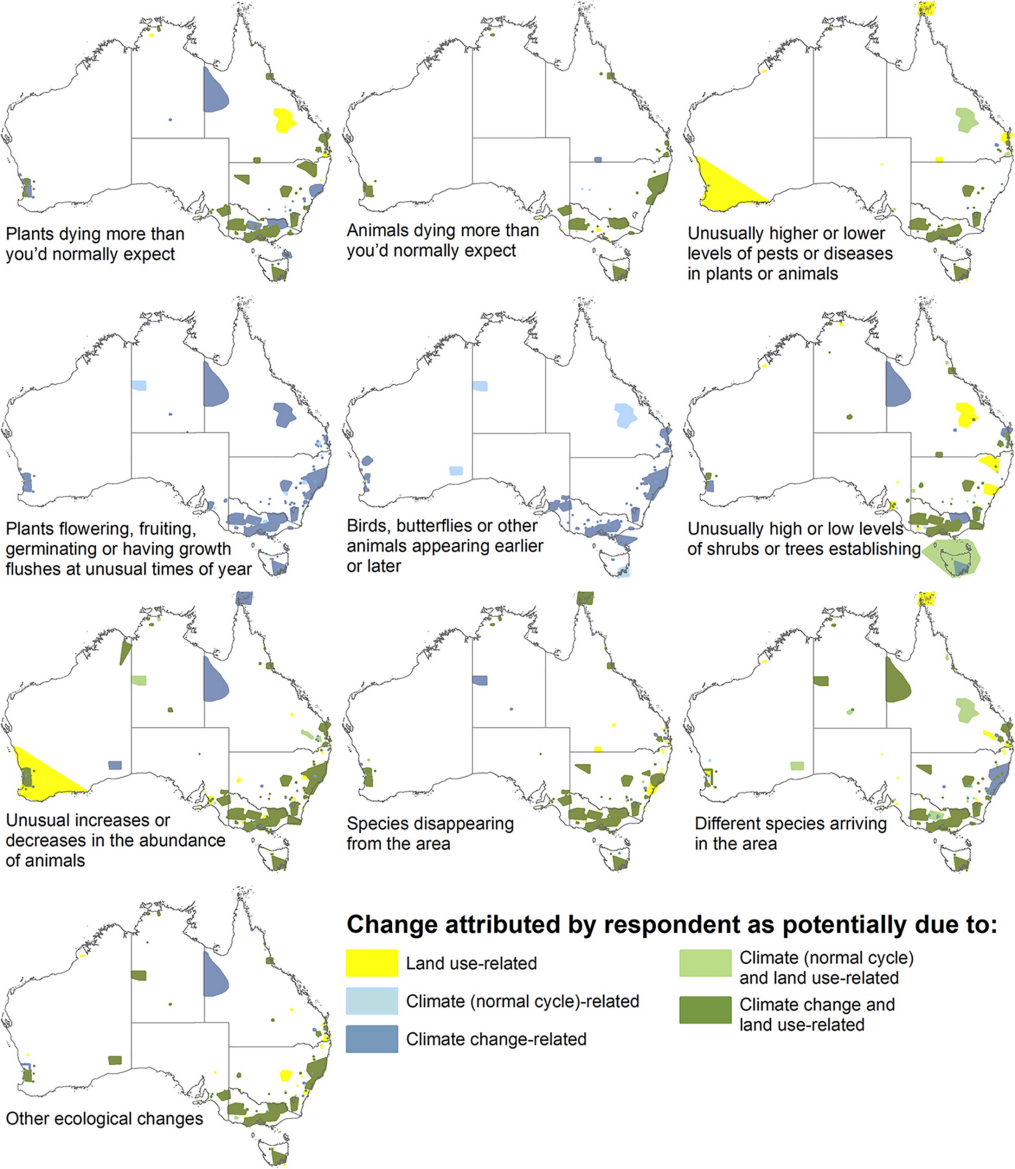
Location of ‘selected areas’ for which respondents had observed an ecological change relevant to each primary change type. Uses Part 1 multiple-choice scores and shows also where change was attributed to combinations of potential land use, climate (scored as ‘normal cycle’) or climate change (scored as unprecedented or climate change) drivers.

Of the ten primary change types considered, the most common types of perceived change in Part 1 multiple-choice responses were changes in animal abundance and the arrival of new species, each observed by over 60% of respondents. However, when considering only changes attributed to potential climate change-related drivers, plants dying, changes in plant phenology, and changes in animal abundance were the three most frequent observations (reported by 39–45% of respondents); high reporting of phenological change in particular is consistent with the predominance of this type of climate change-related observation globally [5, 13]. The least common types of change reported were animals dying and changes in levels of pests and diseases, and these were also the least commonly observed in response to climate change-related drivers. No strong geographic patterns were evident in reporting of the different types of ecological change (Fig 2).

**Fig 3:**
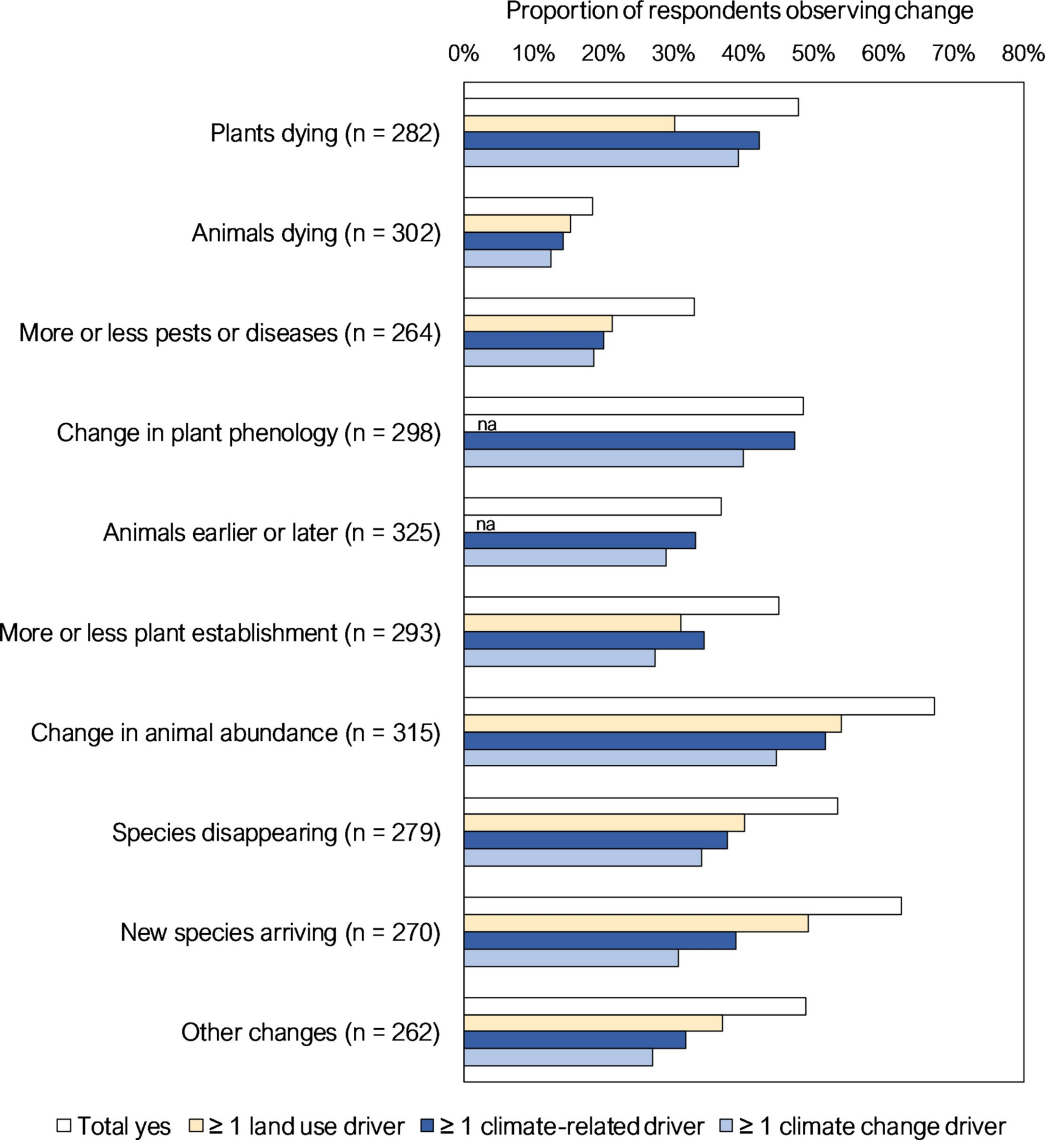
Proportion of respondents observing each of the ten investigated types of change. Refers to Part 1 multiple-choice questions, and includes proportion for each with attribution by respondents to land use, climate and climate change drivers. For summary purposes, ‘potentially climate-change related’ includes climate drivers scored by respondents as ‘unprecedented’ or as ‘climate-change-related’ (break down into those two groups is shown in S2 Appendix: Figure C).

For most types of change, respondents attributed the observed change with similar frequency to climate-related and land use related drivers (in many cases to both), with only slightly less frequent attribution to climate change-related drivers (Fig 3). This resulted in the predominant attribution being a combination of unprecedented climate/climate change drivers (hereafter ‘climate change drivers’) and land use drivers, except for phenological change that as expected was mostly attributed to climate change alone (Fig 2).

Drought, heatwaves and warming conditions were the three most commonly cited climate-related drivers of ecological change across the ten primary change types in Part 1 multiple-choice questions (S2 Appendix: Figure C), with abnormal weeds, pests or diseases also commonly important for a subset of change types (animals dying, more or less plant establishment, change in animal abundance, species disappearing or arriving and other changes). Other types of climate-drivers were still represented across most change types, including hot fires, cold snaps or frost, storms or cyclones, and flooding or heavy rain. In the majority of cases, climate-related drivers were scored as unprecedented (e.g. unprecedented floods or droughts) or related to climate change, with generally smaller proportions considered part of normal cycles (e.g. normal heatwaves, droughts or disease outbreaks, S2 Appendix: Figure C).

### Relationships among respondent demographics and perceptions

Of the demographic variables collected, only climate change belief (i.e. whether the respondent believed that climate change is happening, and if so whether humans are causing it), environmental belief (i.e. whether the respondent believed the environment is generally improving, staying much the same, or generally worsening) and whether the respondent was an ecological/biological researcher, were significantly related to the number of primary changes observed by respondents and attributed to any of land use, climate or climate change drivers (S2 Appendix: Table C). Number of primary change types attributed to land use drivers only showed a very minor positive relationship with environmental belief (Adjusted R^2^ = 0.018, P = 0.026). For all other drivers, the best model explaining the proportion of primary change types to which the respondent answered yes included environmental belief and whether the respondent was an ecological researcher: those with stronger belief that Australia’s environment is declining were likely to have observed a higher number of ecological changes, and ecological researchers were more conservative in the number of changes they identified. This pattern was distinctly strongest for the subset where climate change drivers were nominated, with the model explaining 17.6% of the variation (Table 3, S2 Appendix: Figure D).

**Table 3:** Best statistical models to explain responses to Part 1 multiple-choice questions using Part 3 demographic explanatory variables.

	#Yes		#Yes with land use driver		#Yes with climate driver		#Yes with climate change driver	
	coeff	P	coeff	P	coeff	P	coeff	P
Environmental belief	0.55	<0.001	0.30	0.026	0.81	<0.001	1.23	<0.001
Ecological Researcher (yes)	-0.56	<0.001	na	ns	-0.67	<0.001	-0.72	<0.001
Adjusted R^2^	0.105		0.018		0.142		0.176	

#Yes refers to the total number of the ten primary change types respondents had observed in their selected area (for any drivers, or for land use, climate or climate change drivers only); coeff, coefficient; na, not applicable; ns, not significant

### Overview of the anecdotes

Text anecdotes recorded by Part 2 respondents (in total comprising c. 62,000 words) afforded substantial ecological detail to the Part 1 multiple-choice results, providing a rich array of examples of biotic change across scales from individuals to landscapes. Sixty-five percent of respondents (212/326) described at least one text anecdote that met our qualifying criteria, and of these, each provided an average of 2.5 (range 1–10) anecdotes after partitioning (see Table 2) (summing to 520 anecdotes in total). These included 320 anecdotes attributed to likely or possible climate change drivers (drawn from 158 respondents), noting many individual anecdotes included multiple linked changes. Timeframes were provided for 64% of the 224 unpartitioned (pre-processed) anecdotes. Of these, 63% of ecological changes were considered to have begun sometime in the past 20 years (1997 to 2016), with 80%, 90%, and 95% in the past 30, 40 and 50 years (since 1987, 1977 and 1967) respectively. Most (93%) were considered to be ongoing or to have continued to 2017.

Ecological changes were reported across a wide range of ecosystem types, with an emphasis on woodlands and forests, and including pasture, crop, urban and garden environments (Table 4). They were most commonly reported for trees, birds, shrubs and mammals (in decreasing order), but many plant and animal groups were represented (Table 5). There were also 11 anecdotes reporting on microbes (7 with climate change drivers), and 28 reporting on whole-of-ecosystem impacts (16 with climate change drivers).

**Table 4:** Number of anecdotes specifying occurrence of ecological changes in particular ecosystem types.

Ecosystem	# anecdotes (any driver) ^§^	# anecdotes (cc driver) ^§^
Woodland	107	80
Forest	88	70
Rainforest	30	26
Aquatic/riparian	24	20
Grassland/herbland	24	15
Shrubland	24	16
Orchard/garden	22	17
Wetland	18	18
Coastal	14	11
Pasture	14	8
Crop	5	4
Urban	4	3
Saline habitat	3	2

# number of

^§^attributed to any or at least one climate change-related (cc) driver

**Table 5:** Types of organisms observed to have undergone recent ecological change in Australia.

Type of organism	# anecdotes (any driver) ^§^	# anecdotes (cc driver) ^§^	Type of organism	# anecdotes (any driver) ^§^	# anecdotes (cc driver) ^§^
**Animals**	**312**	**174**	**Plants**	**478**	**312**
Birds	115	64	Trees	164	106
Mammals	76	36	Plants unspec.	136	88
Invertebrates	42	35	Shrubs	77	45
Animals unspec.	33	13	Forbs	47	32
Amphibians	21	12	Graminoids	36	27
Reptiles	20	11	Lower plants	12	9
Fish	5	3	Climbers or vines	6	5
**Microbes**	**11**	**7**	**Ecosystems**	**28**	**16**

# number of

^**§**^attributed to any or at least one climate change-related (cc) driver

**Fig 4:**
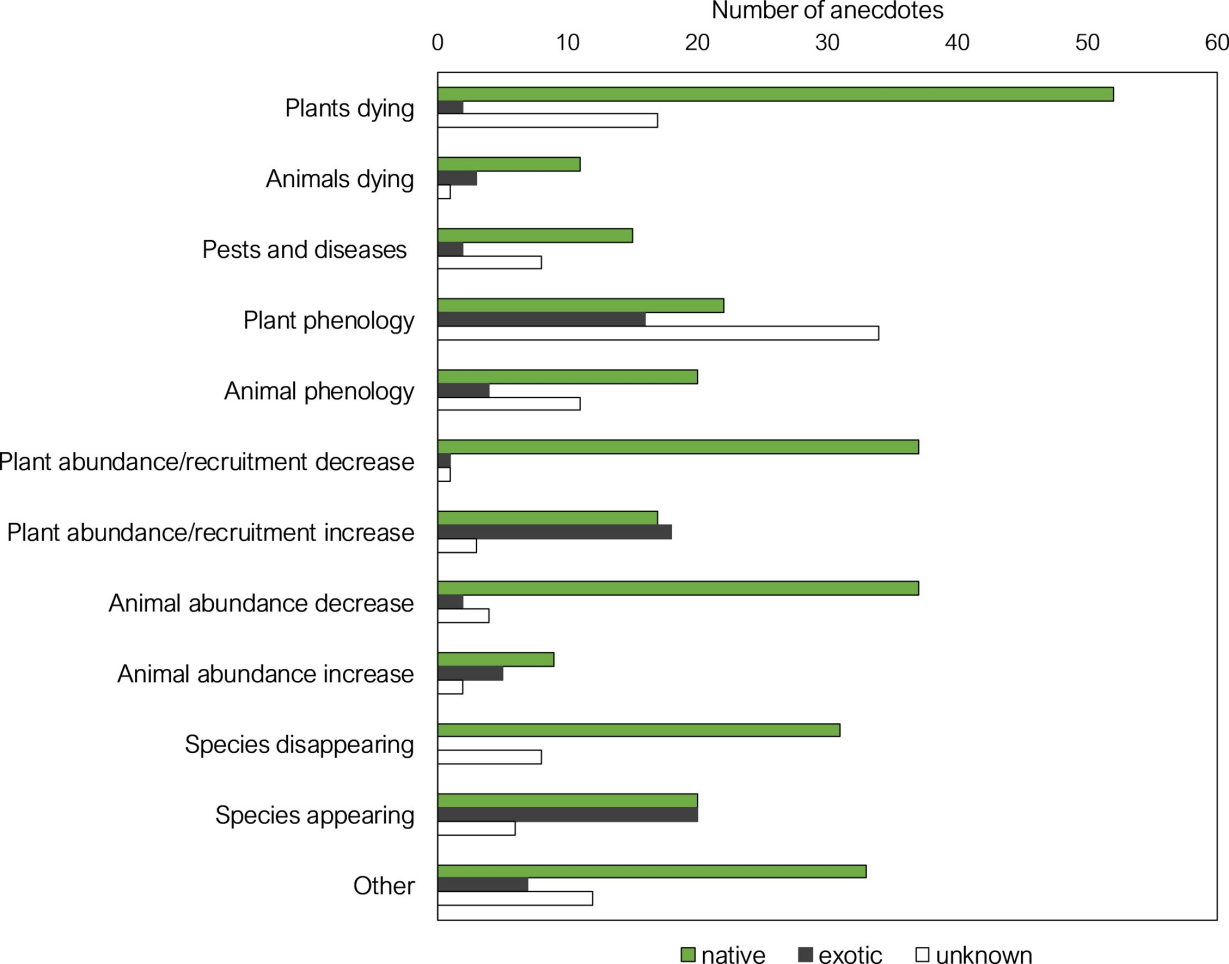
Number of anecdotes reporting climate change impacts on native versus exotic species. Shown against the different primary change classes, with changing plant and animal abundance partitioned to increasing and decreasing abundance. Nearly all cases for pests and diseases involved increases; native and exotic scored for the host species.

Consistent with Part 1 multiple-choice survey results, anecdotes describing ecological changes comprised numerous examples representing each of the primary change questions (Fig 4, Table 6), as well as additional elements of the ecological impact framework (Table 1). In particular, the latter involved anecdotes highlighting higher-level changes to communities and ecosystems, such as changes in ecological interactions, community structure, composition and productivity, as well as a single example of perceived genetic change involving hybridization on the North Coast of New South Wales: ‘*Regenerati[ng] species are often hybrids with species coming south* …*e*.*g*. *wild (not planted* …*) Bangalow Palms* [Archontophoenix cunninghamiana] *are often a Bangalow/Alexander palm* [Archontophoenix alexandrae] *hybrid indicating warming*’ (Anon.).

**Table 6:** Number of text anecdotes for each primary change type with respondent attributions to different drivers, highlighting interactions among climate and land use drivers.

	Plants dying	Animals dying	Pests & diseases	Plant phenology	Animal phenology	Change in plant abundance & recruitment	Change in animal abundance	Species disappearing	Species appearing	Other
**Likely climate change driver**	**45**	**13**	**19**	**48**	**26**	**51**	**44**	**25**	**35**	**41**
Likely land use driver	12	2	6	2	1	21	15	8	11	12
No attribution to land use	33	11	13	46	25	30	29	17	24	29
**Possible climate change driver**^**1**^	**25**	**3**	**5**	**17**	**7**	**27**	**12**	**14**	**6**	**19**
Likely land use driver	5	1	1	0	2	8	4	5	1	4
No attribution to land use	20	2	4	17	5	18	8	9	5	15
**Climate-only driver**^**2**^	**14**	**0**	**0**	**2**	**1**	**18**	**15**	**9**	**9**	**12**
Likely land use driver	4	0	0	1	0	9	8	6	4	2
No attribution to land use	10	0	0	1	1	7	7	3	5	10
**No attribution to climate**^**3**^	**13**	**5**	**8**	**1**	**1**	**49**	**59**	**13**	**19**	**21**
Likely land use driver	6	2	4	1	1	31	41	9	15	14
No attribution to land use	7	3	3	0	0	6	8	4	3	3
**Total with climate change drivers**	**70**	**16**	**24**	**65**	**33**	**78**	**56**	**39**	**41**	**60**
**Total with land use drivers**	**27**	**5**	**11**	**4**	**4**	**69**	**68**	**28**	**31**	**32**
**Total with invasion drivers**	**14**	**4**	**7**	**2**	**0**	**34**	**25**	**14**	**13**	**12**
**Grand total**	**97**	**21**	**32**	**68**	**35**	**145**	**130**	**61**	**69**	**93**

^**1**^includes one case interacting with ecological restoration drivers

^**2**^includes two cases interacting with ecological restoration drivers

^**3**^includes 28 cases involving ecological restoration drivers, most associated with plant or animal recruitment or abundance.

An average of 70% of anecdotes in each of the primary change classes included a likely or possible climate change driver (noting that in Part 2 we requested anecdotes involving climate change when available). On average, 25% of these also included a likely land use driver. This was somewhat less than for Part 1 multiple-choice scores, but consistent with these, anecdotes reporting interactions with land use drivers were rare for phenological change (Table 6). Reported land use drivers included impacts of agricultural development (e.g. habitat loss, fragmentation, livestock grazing, nutrient enrichment, grain spills, chemicals), clearing for linear infrastructure, hydrological interventions (water extraction, dams), forestry (logging, regrowth impacts on water), recreational activities, poaching, prescribed burning and urbanization (S1 Table).

In most cases, anecdotes where land use was perceived to act in concert with climate change involved an additive or synergistic effect of climate and land use resulting in greater ecological pressure to change. For example, fragmentation, livestock grazing, recreational impact, water flow regulation and urbanization were all mentioned as contributing along with climate change to increased tree mortality. Interactions also led to positive outcomes for some species, such as increased grain spillage or urbanization facilitating bird and Flying fox migrations. A less common type of interaction involved impacts of cross-sectoral responses to climate change on biodiversity, such as climate-driven increases in cropping resulting in loss of paddock trees and fence-line remnants.

A number of anecdotes also reported benefits of ecological restoration activities, but only one included an interaction with climate change impacts (Table 6). Biological invasion drivers were occasionally articulated, especially for plant and animal abundance and recruitment (Table 6); about a third of these cases included a climate change driver as well. For example, increases in extreme heat or rainfall events, fire suppression and cat predation were perceived to contribute to the decline of *Pachycephala rufogularis* (Red-lored whistler) in the Riverland region, South Australia.

Notably, anecdotes attributed to potential climate change drivers related far more commonly to native than exotic species across all primary change types (Fig 4). However, there was a greater proportion of examples pertaining to exotic species for new species arriving and increases in plant recruitment or abundance (relating mostly to exotic plant invasions), and for plant phenology (garden or orchard examples were common).

## A synopsis of perceived climate-driven ecological change across a continent

Detailed descriptions and quotations of reported climate change-attributed ecological change are given against each primary change type in S3 Appendix. This is supported by a comprehensive tabulation of results organised by species/ecosystem and change type, and indicating for each example whether the attribution to climate change scored as ‘likely’ or ‘possible’ (Tables A-J in S1 Table). In this section we provide a briefer synopsis of climate change-attributed changes people are perceiving, as viewed from over 150 locations and for hundreds of species and ecosystems across the continent. While anecdotal information has significantly greater uncertainty than evidence from long-term monitoring, some of the anecdotes were consistent with other published Australian studies founded on monitoring or investigation. We thus summarize the anecdotes in this broader context, to highlight consistencies as well as potentially novel reports that are worthy of further investigation.

As evident from Fig 4, the most common climate change-attributed anecdotes respondents reported observing in their local environments involved native species and ecosystems that appeared to be disadvantaged by climate change. There were also varied reports of species’ apparent advantage or adaptation (or sometimes maladaptation) through observations of new arrivals, increases in species’ recruitment or abundance, range shifts or changes in phenology. These changes were often perceived as direct outcomes of climate change (with or without land use interactions). In addition, a wide range of changes were described as secondary outcomes of ecological cascades, including over 50 changes in ecological interactions perceived to be triggered by changes in climate (Table 7).

**Table 7:** Frequency of different types of changes to ecological interactions evident from ecological cascades reported in anecdotes.

Interaction or cascade reported	# reported
Changed plant-herbivore interactions	19
Changed host-pathogen interactions	11
Changed vegetation affecting fauna	9
Changed competitive interactions	6
Changed predator-prey interactions	6
Loss of synchronization	6
Uncoupling of mutualisms	4
Changed host-parasite interactions	1

### A predominance of decline: Plants

Plant species that appeared to be disadvantaged by climate change in the respondents’ locations were typically native, with decline observed either as direct mortality (Tables A, B in S1 Table), increases in pest and disease loads (Table C in S1 Table), reduced recruitment or abundance (Tables F, G in S1 Table), or complete disappearance of plant or animal species from a location (Table H in S1 Table). For plants, such reports included numerous direct observations of individuals dying, often over large areas. These involved at least 55 native species (including 27 *Eucalyptus*, five *Banksia*, five *Melaleuca*, and three *Acacia* species) and only two exotic plant species (*Lantana camara*, *Pinus radiata*). A number of these were consistent with potential climate-related mortality or dieback reported elsewhere, including in *Corymbia calophylla* and *Banksia menziesii* in south-western Australia [30, 31], *E*. *moluccana* in the Sydney basin, *E*. *delegatensis* (due to fire, Hoffmann, Rymer [18] and references therein) and *E*. *camaldulensis* in south-eastern mainland Australia [2], *E*. *gunnii* and *E*. *viminalis* in Tasmania [32, 33], and mangrove species in the Gulf region, Northern Territory/Queensland [34]. Along with the considerable list of other plant species respondents had observed dying, major mortality events such as unprecedented ‘*death of* Eucalyptus tetrodonta *in extensive areas of the Gulf woodlands/open-forests’* (Anon.), ‘*tens of thousands of dead* [Melaleuca] *trees’* at Princess Charlotte Bay (north-eastern Queensland; Simon Thompson and the Lama Lama Land Trust), ‘*large areas of Mulga* [Acacia aneura] *and Yapunya* [Eucalyptus ochrophloia]’ reported to have died in south-western Queensland (Anon.), and fire related mortality in *Hakea lorea* (Long-leaf corkwood) in north-western Queensland, may be less well-known reports of substantial mortality worthy of further investigation (Table A in S1 Table).

More generally in relation to previous studies of tree mortality, an Australia-wide review analysed 15 reported die-off events between 1891–2013 that involved 10 species in common to the 55 species reported in our study (sometimes in similar locations), as well as an additional 28 woody species. Through analysis of weather data in association with these events, Mitchell, O'Grady [35] showed that they occurred when water deficits and maximum temperatures were beyond 98% of the observed range of drought intensity. Notably, many of the events reported in Mitchell, O'Grady [35] occurred before 1990 (particularly the 1960s), highlighting the challenge of distinguishing climate change-related ecological change from change driven by normal drought- and heat- cycles. For example, *E*. *macroryncha* mortality events were reported in three separate anecdotes in our survey (ACT and Victoria). The ACT event was reported as occurring from 2000–2017, whereas Mitchell, O'Grady [35] reported mortality events for this species in the ACT in 1965 and 1982–3. Mitchell, O'Grady [35] estimate that climate change is expected to increase the frequency of such events from 1 in 24 to 1 in 15 years by 2050, hence to better enable attribution to climate change, we suggest future monitoring should aim to capture frequency and extent as well as location of occurrence and species involved in mortality events.

Increased disadvantage to plant species due to climate change was also reported in our survey as increases in pathogens and invertebrate herbivory, declining regeneration and abundance and species disappearing. Reports of plant decline associated with increased pathogens or herbivory involved at least seven *Eucalyptus*, two *Corymbia*, and two *Banksia* species, and various rainforest plant species, affected by diseases such as *Quambalaria coyrecup* T. Paap. (*Corymbia* canker), *Phellinus sublamaensis* (Lloyd) Ryvarden (White rot) or unnamed viruses, and invertebrates including psyllids, scale, termites, weevils and aphids (Table C in S1 Table). These reports are consistent with Mitchell, O'Grady [35] who found that tree mortality events often included an interaction with invertebrate stressors, and Paap, Brouwers [31] who reported that although increasing pathogens such as *Quambalaria coyrecup* in *Corymbia calophylla* were more prevalent in cool, wet environments, climate change may influence susceptibility by increasing tree stress.

Climate change-attributed declines in plant regeneration or abundance were reported in nearly 40 anecdotes pertaining to native species, particularly trees such as *Eucalyptus*, *Allocasuarina*, *Banksia*, *Callitris* and *Melaleuca* spp. (e.g. Table F in S1 Table), but in few anecdotes pertaining to exotic plants. Other reports referred to forbs and shrubs, for example ‘*ground cover plants*, *including common heath* [Epacris impressa], *blue pincushions* [Brunonia australis], *pink bells* [Tetratheca ciliata] *have disappeared’* in south-western Victoria particularly due to drying conditions (in turn leading to ‘*local extinction of the rufous bristlebird and the long-nosed bandicoot*’; *Dasyornis broadbenti* and *Perameles nasuta* respectively, Table H in S1 Table, Anon.). We found only occasional examples of potentially climate change-related declines in plant species recruitment or abundance reported elsewhere that matched our anecdotal reports, including fire-driven declines in *E*. *delegatensis* [18] (and references therein), and drought driven decline in *E*. *camaldulensis* [2]. Potentially important anecdotes worthy of further investigation include drought and heat-associated declines in abundance and regeneration of *Banksia marginata* near Adelaide in South Australia, mistletoes near Bendigo, Victoria, and *Eucalyptus populnea* in south-eastern Queensland.

### A predominance of decline: Animals

Consistent with Part 1 multiple-choice results, animal deaths were less frequently described than plant deaths in anecdotes, potentially due to lower apparency (Fig 4, Table B in S1 Table). Two of the major animal mortality events reported as potentially climate change-related have been documented elsewhere, in particular, multiple mass die-offs in *Pteropus* spp. (Flying foxes, e.g. Welbergen, Klose [36]), and observations of mass death of *Emydura macquarii* (Murray River turtle) at Lake Numalla in south-western Queensland [37]. Anecdotes we have not seen reported elsewhere included sudden death of tadpoles (likely *Lymnodynastes dumerilii* Banjo frogs and *Litoria ewingii* Brown tree frogs) due to salinization of groundwater associated with sea level rise near the Derwent estuary in Tasmania, and two independent anecdotes of mortality of *Stagonopleura guttata* (Diamond firetails) due to heatwave conditions, in Tharwa, ACT and near Lake Burrendong, NSW (e.g. ‘*whole families died overnight*’ Anon.). More gradual decline was also reported, for aquatic species (crustaceans, Hardyhead fish) with warming and drying of wetlands; *Litorea cyclorhyncha* (Spot-thighed frog), *L*. *moorei* (Motor bike frog), young owls and *Rusa unicolor* (Sambar deer) due to starvation; and possums and *Phascolarctos cinereus* (Koala) due to increased disease susceptibility (see below).

These perceived drivers of animal mortality were further reflected in a wider range of anecdotes describing local decline of at least 36 and local disappearance of at least 34 animal species (Tables A1g,h). A number of cases matched similar records from the literature, although they were typically for different locations, including declines in frogs in south-eastern Australia (e.g. Mac Nally, Bennett [38]), decline in *Phascolarctos cinereus* (Koala); our anecdote in south-eastern rather than south-western Queensland, Seabrook, McAlpine [39], and decline in fish species in South Australia [40]. Additional reports of animal decline highlighted in our survey involved insects, *Cherax quinquecarinatus* (Gilgie) and turtles in the Perth region, trout in north-eastern Victorian mountain streams, *Gallinula tenebrosa* (Dusky moorhen) in Boondall wetlands, south-eastern Queensland and *Hydromys chrysogaster* (Water rat) in dams of central NSW, all purported to be declining due to warming and drying of aquatic or wetland habitat.

Disadvantage to a large suite of terrestrial native animals was also attributed to warming and/or drying more generally. These included declines in invertebrate populations such as *Heteronympha merope* (Common brown butterfly) in north central Victoria, mosquitoes in south-western Australia, leeches on the NSW north coast, and reptiles such as *Pogona* sp. (Bearded dragon) and *Tiliqua rugosa* (Bobtail/Shingleback) in Perth and central Victoria. At least 21 native bird species (and only one exotic bird) were involved, as well as mammals such as possums, *Antechinus* sp. (Antechinus) and *Tachyglossus aculeatus* (Echidna) in central NSW, *Isodon* (Short-nosed bandicoot) near Adelaide, South Australia, and ‘*catastropic local extinction*’ of *Thylogale stigmatica* (Red-legged pademelon) and *Notamacropus dorsalis* (Black-striped wallaby) in the Forty Mile Scrub, south-eastern Queensland [Pers. comm. Mark Weaver (retired—formerly Queensland Parks and Wildlife Service)].

Notably, a large proportion of anecdotes about animal decline were associated with purported climate-induced ecological cascades that altered ecological interactions. These included declines associated with perceived climate change-related increases in common parasite and disease loads, including *Batrachochytrium dendrobatidis* Longcore, Pessier & D.K.Nichols (Chytrid fungus) in frogs (e.g. [41], *Chlamydophila pecorum* (Fukishi and Hirai 1992) (Chlamydia) and other diseases in *Phascolarctos cinereus* (Koala) [42], Hendra virus in bats and horses [43], *Sarcoptes scabiei* (Mange) in *Vombatus ursinus* (Common wombat) [44], and *Haematobia exigua* (Buffalo fly) in livestock. Although these diseases and parasites are well-known burdens to these species, impacts of climate change on parasites and diseases are generally not well documented [13]. Nevertheless, *Haematobia exigua* (Buffalo fly) have been reported in the media to be moving south due to milder winters [45], and general increases in pests and diseases are expected due to increases in animal stress [42]. Our survey also highlighted potentially climate change-related increases in *Ixodes holocyclus* (Paralysis tick) burdens in European foxes in south-eastern Queensland, and stress dermatitis in *Trichosurus arnhemensis* (Northern brushtail possum) in Darwin, NT, that are worthy of further investigation.

Plant-animal cascades resulting in local disappearance or decline of animals involved climate change-attributed vegetation (habitat) change affecting numerous small bird species (scrubwrens, fairywrens, thornbills and robins), *Macropus rufogriseus* (Red-necked wallaby) and *Wallabia bicolor* (Swamp wallaby) in south-eastern Queensland; and *Dasyornis broadbenti* (Rufous bristlebird) and *Perameles nasuta* (Long-nosed bandicoot) in south-western Victoria (see also discussion under plant decline). Altered quality or abundance of plant food sources was reported to cause declines in thynine wasps near Melbourne, Victoria; *Phascolarctos cinereus* (Koala) and marsupial gliders (Petauridae) in central Victoria; and small possums and birds in Wallum heaths of south-eastern Queensland.

Other types of cascades resulting in animal declines included uncoupling of mutualisms and competitive displacement. An example of the former was for *Westralunio carteri* (Freshwater mussel), which *‘relies on host native fish to reproduce and disperse [46]. Drought may mean that host fish cannot reach populations of adult mussels, so the significant range decline observed in this species [47] may have occurred in part because some mussel populations in perennial pools have become isolated by drought and died of old age with no reproduction’* (Swan Coastal Plain, WA, Assoc. Prof. Belinda Robson, Murdoch University). Another example of uncoupling was for *Dicaeum hirundinaceum* (Mistletoebird), with reports such as *‘marked decrease in Mistletoes and so marked decrease in Mistletoebirds’* (Glenise Moors) (reported independently in north central Victoria and south-eastern Queensland). An example of competitive displacement was the replacement of *Artamus cyanopterus* (Dusky woodswallow) by newly appearing *A*. *personatus* (Masked woodswallow) and *A*. *superciliosus* (White browed woodswallow), near Lake Burrendong, central NSW.

### A predominance of decline: Change in ecosystem processes

In addition to these species-focused anecdotes, local declines in biodiversity were also reported in relation to changes in ecosystem processes (Table J in S1 Table). These included at least 12 anecdotes describing changes in fire regimes, typically pertaining to one of two previously reported types: increase in large, intense fires in mountainous landscapes of south-eastern Australia, and strengthening of rain-growth-fire cycles in arid Australia. The former included ‘*dry lightning over 2 days burned* …*thousands of hectares of climax rainforest* …*12*,*000 years of peat accumulation destroyed* (in NW Tasmania, Deborah Hunter, Mole Creek Caving Club] and loss of *Eucalyptus delegatensis* (Alpine ash) forest in Victoria with ‘*litter burnt to mineral earth* …*lizards* …*gone* …*rivers* …*so warm the trout wouldn’t bite* …*[trees] having difficulty regenerating*’, Jim Blackney, similar to Bradstock, Penman [48] and Harris, Beaumont [2]. A number of anecdotes reported changed rain-grass-fire cycles in arid Australia, comparable with reports in Harris, Beaumont [2], and were linked in one anecdote from north-western Queensland with decline in local long-lived native trees such as *Hakea lorea* (Long-leaf corkwood).

Increased erosion was reported in nine cases, associated with flooding, sea level rise or reduced ground cover, and with outcomes such as invasions and loss of native understorey species. Another change in ecosystem processes involved several reports of increasing salinization, resulting for example in mass *Melaleuca* mortality (as mentioned in our discussion on plant mortality) and shifts in vegetation type in north-eastern Queensland (potentially comparable with reports of contractions in *Melaleuca* forest in the Northern Territory due to penetration of saltwater [49]). On the other hand, one anecdote from the Brisbane River (south-eastern Queensland) reported decreasing salinity levels due to increased freshwater influx, resulting in mangrove die-off with minimal recovery.

### Apparent advantage or adaptation: Increasing abundance and range shifts

By contrast with anecdotes reporting disadvantage to native species and ecosystems, there were fewer anecdotes of native species locally increasing in recruitment, health or abundance, or apparently adapting through local or regional range shifts (Fig 4). Nevertheless, our survey included at least 20 climate change-related anecdotes of native species newly arriving to an area (including 14 native bird species), and 26 anecdotes involving native species increasing in abundance (including 13 native plant species, nine native bird species, six invertebrate species) (Tables F, G, I in S1 Table). There were similar numbers of anecdotes for exotic species in these categories, but most were associated with exotic plants rather than animals.

Arrivals and increases in animals included a number of records documented in earlier studies, although in different locations. These include increases in *Eolophus roseicapillus* (Galah), *Trichoglossus moluccanus* (Rainbow lorikeet) and *Cacatua galerita* (Sulphur crested cockatoos), documented by Davis, Taylor [50] for the Sydney region, compared with NSW southern highlands in our anecdotes, but both partly attributed to periods of decreased rainfall inland; and arrival of Flying foxes (‘*recent white box flowering has attracted a bat colony to Murrurundi* …*not seen before’*; Anon.). The latter is potentially consistent with observations of >1000 km range shifts observed in *Pteropus alecto* (Black flying fox), but Roberts, Catterall [51] found climate change alone was inadequate to explain the degree of southward movement. In addition, survey respondents reported observations of upslope migration of *Phascolarctos cinereus* (Koala) at Tambourine Mountain, south-eastern Queensland, away ‘*from the increasingly hotter and habitat-depleted lowlands below*’ (Anon.); fireflies in north-coastal New South Wales; *Ixodes holocyclus* (Paralysis tick) moving up to the NSW Southern Tablelands; and *Artamus personatus* (Masked Woodswallow) and *A*. *superciliosus* (White Browed Woodswallow) ‘*seeking water and habitat in a cooler environment*’ in Central New South Wales (long term observations by Neville Mattick / Hargraves NSW). Exotic *Rusa unicolor* (Sambar deer) were also reported to be increasing in activity at higher elevations on the Bogong High Plains, Victoria. Southward rangeshifts were described for several species in relation to warming and drying, including ‘*warmer*, *drier conditions bringing crested pigeons* [Ocyphaps lophotes] *and choughs* [Corcorax melanorhamphos] *south’* in Victoria (M.T. Casanova), and *Trichoglossus moluccanus* (Eastern koel) successfully establishing near Bungendore. Locally new appearances of *Litoria caerulea* (Green tree frog) at Willandra Lakes, New South Wales, and another nine native (and only one exotic) bird species at various locations, were also reported, although direction of these shifts were not indicated.

For native plant species, potential examples of advantage or adaptation included recruitment and inland spread of mangroves in north-east Queensland and the Northern Territory in association with sea level rise. An earlier report [52] similarly describes mangrove encroachment in the Northern Territory; although they found that mangrove expansion was more prevalent in upper tidal zones than coastal environments referred to in our anecdote. Other potential newly reported cases of plants increasing include perceived recruitment and expansion of species such as *Eucalyptus gomphocephala* (at the expense of wetland species such as *Melaleuca rhaphiophylla*) in drying wetlands of the Swan Coastal Plain, Western Australia, ‘*Thickening of native acacias on largely open Mitchell Grass Downs country*’ (Anon.), recruitment of *Melaleuca leucadendra* further inland as the sea encroaches mature stands on the Northern Territory coast, and bracken replacing rainforest understorey in the Bunya Mountains (south-eastern Queensland). Observations of increase also extended to non-local native species, including *Acacia longifolia* ‘overtaking’ burnt areas in the Wimmera, Victoria, *Rhagodia candolleana* establishing ‘*at a remarkable rate in the last 20 years’* (Anon.) near Beachport, South Australia, and dry rainforest species establishing in wetlands of south-eastern Queensland.

By contrast with only a single report of a newly appearing native plant species (‘*Fig trees* [Ficus sp.] *are appearing where none were before*’ Anon.), 13 exotic plant species were reported as newly appearing or migrating altitudinally in response to climate drivers (e.g. altitudinal spread of exotic plants such as *Lantana camara*, *Ageratina* spp. and *Chrysanthemoides monilifera* in New South Wales and South Australia), and another 12 exotic plant species were reported as increasing in abundance. Further consequences of these invasions were commonly reported, including reduced native plant regeneration or abundance, increased fire risk, facilitation of arrival of a locally new (possibly exotic) moth species, and increased abundance of native Bell miners (in turn resulting in tree dieback).

At the community level there were also observations of vegetation structural and compositional shifts and changes in ecosystem productivity. The former represent local losses of prior species and vegetation types, but also indicate that adaptation is occurring at the community level. In drying environments these observations included wetlands transitioning to dryland communities (Perth region, WA), coastward shifts in south-west Australian wheatbelt and forest species, and ‘*drier forests* …*reverting to a more woodland form*’ (Dandenong Ranges, Victoria; Dr David Jones). Another example involved *‘a suite of fire resistant*, *suckering rainforest species* …*taking over a large proportion of the wet eucalypt communities’* (Anon.) on the Atherton Tableland, Queensland, purportedly in association with rising atmospheric CO_2,_ consistent with earlier reports of rainforest expansion [53]. Observations of increasing ecosystem productivity with beneficial outcomes for biota included perceived shorter winters in southern Tasmania, resulting in longer plant growing seasons and subsequent increases in small marsupials, reptiles and small birds, in turn leading to an increase in raptors (that eat the small fauna); and greater frequency of wet conditions supporting more herbs, grasses, *Atriplex* and *Maireana* species and reducing bare ground on the Nullarbor Plain, Western Australia. In an agricultural context, *Pennisetum clandestinum* (Kikuyu) pasture was reported to be ‘*now growing all year round rather than being frosted off and unproductive in winter (May to November)’* in the Kangaroo Valley, New South Wales, eliminating the need for winter fodder supplementation (Greg Thompson, Brogers Creek Landcare).

### Apparent advantage or (mal)adaptation: Phenological change

Apart from these observations pointing to changing species abundances and distributions, organism and population-level impacts of climate change were also commonly reported by survey respondents as phenological change, often attributed to climatic changes associated with warming (e.g. shorter winters, hotter heatwaves, lack of cold stratification). Phenological changes involving significant advancement or change in flowering time in plants, and changes in migratory bird arrivals and departures, are some of the few well-demonstrated climate change impacts in Australasia [13, 14, 17, 54]. However, the number of plant species for which phenological data are available is relatively small in Australia [55], with 70% of plant records in Chambers, Altwegg [14] applying to grapevines or apples, and a limited number of studies reporting responses in eucalypts, woodland forbs, coastal and alpine native species [18, 54, 56]. Our anecdotes are consistent with frequent reports of phenological change in planted woody deciduous species [14], including observations of early or altered flowering from Queensland, New South Wales, South Australia and Western Australia, as well as other types of change (e.g. deciduous trees retaining their leaves ‘*right into winter*’ (Wheatbelt, WA Anon.). In addition, respondents reported changes in flowering in native plant species (*Eucalyptus*, *Corymbia*, *Acacia*, orchids, *Banksia menziesii*, and *Stenocarpus sinuatus*) across all Australian states. Other types of phenological change reported included ‘*the small leafed privet* [Ligustrum sinense] …*producing viable seed up to 3 months earlier than 12 years ago’* (Maggie Wheeler), unseasonal fruiting in *Archontophoenix cunninghamiana* and shorter growing seasons in orchids (e.g. *Caladenia amoena* in Melbourne), annuals and native forbs.

On the other hand, evidence for migrational phenology change in birds is available for a diverse suite of Australian species: Chambers, Beaumont [17] tested long-term data sets for 52 bird species from 10 sites in southern Australia, and found trends in at least one aspect of migrational phenology for 38 of these. Our data shared observations for earlier arrival in *Eudynamys scolopaceae* (Common koel), *Scythrops novaehollandiae* (Channel-billed Cuckoo) and *Chrysococcyx basalis* (Horsefield's bronze-cuckoo, not significant for Chambers, Beaumont [17], but otherwise our reports for birds were substantially less comprehensive. Nevertheless, our data included some additional observations, including *Acanthagenys rufogularis* (Spiny cheeked honeyeater) ‘*more likely to over-winter at Lake George* [SA] *than previously*’ (Anon.), earlier winter arrival of *Eopsaltria australis* (Yellow robin) in south-eastern Queensland, and altered migration habits of *Strepera graculina* (Pied currawong) in south-western and north-eastern New South Wales.

Chambers, Altwegg [14] emphasize more generally that terrestrial animal phenological data in Australia have poor taxonomic coverage, including only three studies for reptiles and none for mammals, amphibians or invertebrates other than butterflies (for which one anecdote in our study concurred with earlier emergence of *Heteronympha merope* (Common brown butterfly) by Kearney, Briscoe [57]. Our respondents occasionally described altered timing of events relating to these less-well reported animal groups or to different types of phenological change. For invertebrates these included earlier swarming of bees in south-western Victoria (‘*Bees used always to swarm in the first week of November*, *now it can occur in late September or October’*, M.T. Casanova), earlier emergence of moths in the Hunter Valley, New South Wales, and lengthened seasons for *Ixodes holocyclus* (Paralysis tick), aphids and psyllids in south-eastern Queensland, on the New South Wales Southern Tablelands and in the Australian Capital Territory respectively. Other types of phenological change reported included earlier hatching of reptiles in south-eastern Queensland, earlier nesting of birds across four states and territories, and earlier breeding in *Petaurus breviceps* (Sugar glider) in north-coastal New South Wales.

These phenological changes demonstrate phenotypic plasticity, which could indicate early responses in individuals and populations that may be adaptive (i.e. enhancing performance or persistence) or mal-adaptive (i.e. reducing performance or persistence) [14]. Ultimate outcomes of phenological change for species and ecosystems depend on flow-on effects to population demography and interspecific relationships, but such reports are sparse in the literature [14]. Flow-on effects reported within species demonstrating phenological change in our survey included multiple successful breeding events per season in birds; breeding failure in moths due to cool weather returning after early emergence; plants being frosted after early growth flushes; reduced reproductive success and survival in orchids due to shortened growing seasons; and decline in butterflies, reptiles and bird chicks due to temporal disconnection from food supplies. Across species, early or strong flowering events benefited some bats and birds, and ‘*increased season for paralysis tick* …*resulted in a marked decline in fox* [Vulpes Vulpes] *numbers*’ (Anon.). On the other hand, reports suggested that earlier seeding in the exotic shrub *Ligustrum sinense* led to lower competitiveness of regenerating native plants, and earlier breeding in Sugar gliders led to starvation of owl chicks. At the ecosystem level, changed growing season lengths were observed to result in both increases and decreases in plant and animal productivity, and increases in plant productivity sometimes flowed on to increased fire frequencies, as well as positive outcomes mentioned earlier.

## Discussion

Our survey showed that people are already perceiving a multitude of simple and complex climate change impacts on Australia’s biodiversity and ecosystems, consistent with expectations from models projecting moderate to high degrees of change in biodiversity across Australia by 2050 (e.g. [58]). Compared with more formal or intensive approaches to identifying climate change impacts [26, 59], our methodology provided a large volume of mostly unsubstantiated anecdotes, capturing a broad spectrum of potential climate change-driven changes as viewed from local, observational perspectives. Our approach targeting people with close connections to the environment permitted many perceived changes to be identified to species or ecosystem level, and our mapping tool enabled linking of anecdotes with location information. While it is beyond the scope of this study to confirm each anecdote, the suite of examples substantially extends the detail previously reported for the continent (e.g. [2, 13, 14, 17, 18]), and offers a strong foundation for further investigation.

### A collective view of change across a continent

Collectively, the anecdotes create a picture of widespread, often subtle or gradual changes (phenological shifts, changing abundances, range expansions and contractions) across the continent, punctuated by extreme events such as fires, unprecedented droughts and other causes of mass mortality. Compared with other studies that describe selected case examples of climate change impacts (e.g. [2, 18]), our systematic methodology helped to convey an overarching view of the perceived array of climate change impact types on biodiversity and ecosystems across Australia. In particular, the overarching pattern indicated by the anecdotes suggests that people are more often noticing climate change losers than winners in their local areas, but with potential early evidence of ‘adaptation in action’ through arrivals and range shifts (particularly for native birds and exotic plant species), and compositional and phenological change. It is notable that the ‘losers’ observed were typically native species, suggesting that decline or disappearance of exotic species is less notable to observers, and/or that the ruderal nature of exotic species makes them less vulnerable to environmental change. Conversely, there was a higher proportion of exotic species among observed new arrivals; possibly these stand out to observers more than familiar native species arriving, or again are ruderal species more able to take advantage of unfilled niches as conditions change [60].

Anecdotes also described higher-level ecosystem changes including altered ecosystem productivity and catastrophic impacts of changing fire regimes, as well as more than 50 examples of ecological cascades. The latter can be extremely difficult to predict, in contrast with lower levels of complexity that are the focus of most data streams [6, 61]. Scheffers, De Meester [4] noted for example that changes in competitive interactions have been poorly reported, and Chambers, Altwegg [14] noted the lack of Southern Hemisphere evidence for consequences of phenological change on species’ demography or synchrony with interacting species; a suite of which were reported in our survey. Across the Australian continent, reported ecological cascades most commonly involved animal decline associated with increased parasite and disease loads, vegetation (habitat) change, and altered quality or abundance of plant food sources (and occasionally uncoupling of mutualisms); or plant decline due to altered plant-herbivore interactions.

While our study raised many potentially new examples of climate change impacts on biodiversity and ecosystems across Australia, there is also a range of striking impacts reported in the literature that were not reported in our study. A sample of these includes declines in water pythons [62], platypus [63], and rainforest possum species [18]; heatwave driven shrub mortality in Tasmania [64]; Sphagnum moss decline in south-eastern Australia and Macquarie Island [65]; and changes in Alpine dynamics [18, 66]. Given this, we suggest that the anecdotes revealed in our survey, while substantially extending the suite of potential climate change impacts on biodiversity and ecosystems in Australia, is likely to represent only a small subset of the amount of change being perceived across the continent.

### Methodological biases

Notwithstanding the apparent emphasis on species’ and ecosystem decline by survey respondents, it is important to recognize that other types of biological change are likely to be occurring at small scales that are difficult even for keen observers to see, potentially biasing conclusions. This pertains particularly to morphological or genetic change that is potentially adaptive. Emerging studies measuring plant or animal specimens from the wild, in collections or in common gardens, for example, show that such adaptations have already begun to occur. These include genetic changes in *Drosophila* (e.g. [67]), shifts in body size for numerous bird species [68], sex change in reptiles [69] and shifts in leaf traits such as width, area and thickness [70, 71]. Such changes may be facilitating persistence of species and resilience of communities and ecosystems, which would appear as ‘lack of change’ to an observer at the population to ecosystem scale, rather than resulting in a report of change.

Other types of bias were also evident from the survey results. First, anecdotes were skewed towards forests and woodlands, potentially resulting from the spatial bias reflecting human population densities (Fig 1), and towards more dominant or apparent species such as trees and birds. These trends are consistent with the focus of climate change impact studies worldwide, although with lower representation of aquatic ecosystems, and lower representation of invertebrates which are convenient experimental subjects due to their short life cycles [61].

Second, our respondents were predominantly (non-Indigenous) people with long-term connections to Australian environments and who believe that climate change is happening. A surprisingly high proportion of respondents (78%) attributed perceived changes to climate change, which may reflect both their deep local knowledge and/or their environmental beliefs. On this second point, the psychological concept of motivated reasoning [72] posits that people are motivated to reach desired conclusions, which may bias their evaluation of new evidence; that is, people may selectively search their existing memories and beliefs to support a conclusion about a new, but relevant, topic. In the current case, people who believe climate change is predominantly caused by human activity (86% of our sample–a substantially larger proportion than the general Australian population) may more readily attribute observed ecological changes to climate change, as this attribution fits neatly with pre-existing beliefs. Consequently, we would expect the proportion of people attributing ecological changes to climate change to be lower among the general population. Notably though, ecological researchers were more conservative than other respondents in attributing observations to climate change, which may ameliorate potential bias associated with environmental beliefs.

### Veracity of anecdotal evidence

The picture of ecological change revealed by our survey includes significant lack of certainty about both the purported ecological changes and of their drivers, as expected with a methodology that focuses on anecdotes rather than data [20]. While it is not yet possible to evaluate the veracity of our anecdotal reports compared with that of long-term ecological data, several lines of evidence suggest it may be a valuable source of information to complement and guide collection of (but not replace) ecological data. First, prior studies suggest that anecdotal records of changes in climate parameters were often supported by climatic records [9, 21, 22]. Second, people’s observations were generally compatible with changes demonstrated globally and locally. At global scales, the anecdotes conformed with expectations according to global climate change impact frameworks. This includes examples across the spectrum from organisms, populations and species to ecosystems and landscapes ([1, 4], S3 Appendix) although we accept some potential influence of our survey questions in eliciting these [20, 21]. At the local level, respondents described at least 35 examples that had close parallels in the literature. Finally, more than 20 sets of similar anecdotes, involving the same species or close relatives, were reported separately by different respondents (S1 Table).

### Other drivers of ecological change

In a review of combined effects of climate change and land use on biodiversity, Oliver and Morecroft [11] concluded that few studies explicitly acknowledge and account for such effects, and that further research is needed to inform biodiversity projections and conservation decisions. The high proportion of cumulative or interactive effects between climate change and land use reported in our study similarly emphasizes the need for greater consideration of interacting drivers, to better match management responses. Towards this, the anecdotes in our survey provide potential case studies for further examination, for example, interactions among fire suppression, cat predation and climate drivers in the decline of *Pachycephala rufogularis* (Red-lored whistler). This example also emphasizes interactions with biological invasions, although these were less commonly reported.

Two key types of interaction between land use and climate change were identified in our survey: additive or synergistic effects leading to direct impacts on ecosystems and biodiversity, and indirect effects of cross-sectoral responses to climate change impacting on biodiversity. Management approaches to the latter would require stronger integration of social, economic and ecological perspectives in climate adaptation responses, seeking land use changes that result in positive rather than negative impacts on other sectors [8].

### Future directions

A key goal of this study was to explore the value of local ecological knowledge for revealing impacts of climate change. Our survey methodology proved effective in providing a compendium of anecdotes of climate change impacts on biodiversity and ecosystems across Australia, and we have suggested that several design elements (although untested) contributed to this effectiveness. These include (1) a focus on ‘selected areas that respondents knew well’ to elicit anecdotes drawn from deep familiarity and extended temporal observation; (2) use of a custom-designed mapping tool to provide explicit spatial information; and (3) a targeted respondent set with expected close connections with their environment, likely drawing more heavily on expert knowledge than may have been achieved by less-targeted citizen science approaches [73].

Nevertheless, a number of limitations to our approach were evident. While a suite of anecdotes were consistent with existing data, a larger number of the trends and attributions reported remain unsubstantiated. It will be important that follow-up studies of selected anecdotes (e.g. similar to Harris, Beaumont [2] and Hoffmann, Rymer [18]), and comparisons with model predictions (see below), collectively enable further evaluation of local knowledge approaches. Additionally, it could be valuable to validate the local knowledge approach by undertaking targeted surveys in locations that have already had strong ecosystem or biodiversity monitoring programs in place for >20 years.

A second limitation of our survey was the spatial and ecological bias in responses, probably driven by population density, and response from only seven people who identified as Indigenous. Complementary methodologies better suited to remote environments (e.g. phone surveys) and Indigenous people (e.g. workshops on country) may help to overcome this bias, recognising in particular that Indigenous people hold significant knowledge of ecological responses to climate and weather through their seasonal calendars and phenological indicators [74]. These learnings regarding the advantages and disadvantages of our approach can not only be applied to the design of future local knowledge surveys, but could be extended to citizen science platforms, including web-platforms and hand-held applications (e.g. for use by Indigenous rangers). The latter would facilitate ongoing recording of observations of ecological change and attribution of change drivers, and may be particularly valuable in regions where ecological data are sparse.

While a key goal of this paper was to generate a continental-scale picture of current perceptions of climate-driven ecological change across the continent, the detailed anecdotal information collated offers potential further applications. These include: (1) follow-up of selected novel anecdotes to inform climate adaptation management, prioritized on the basis of potential impact on biodiversity, potential for amelioration through management, and relevance to land managers’ regional and social contexts [75]; (2) comparison of anecdotal data with modelled projections of climate change impacts to provide an independent line of evidence for model validation (e.g. using whole-of-biodiversity compositional turnover methods [76]); (3) further exploration of interactions between climate and land use change [11]; and (4) use of the anecdotal records to contribute to designing the focus and location of climate change monitoring networks [77].

Finally, our survey showed that many respondents are observing ecological decline in their local environments, raising the question of how these perceptions influence people’s behavior and well-being. On the one hand, observations of impact are known to heighten perceptions of climate change risk and hence propensity to take action [9, 21, 78]. On the other hand, people value nature not only for functional uses, but also to meet psychological and ethical needs such as sense of place and inter-generational equity [79, 80]. Consequently, it is likely that perceptions of wild areas undergoing catastrophic change, landscape-scale tree mortality, disappearance of species, and even subtle changes, are already affecting people’s psychological well-being. Lost sense of place for example, can lead to ‘solastalgia’ (grief arising from disturbance of familiar environments [81–83]). Indeed, a sense of distress and helplessness was evident in some of the anecdotes. With regard to mortality of *Eucalyptus gunnii* (Miena cider gum) for example, one respondent related: ‘*I have recently spoken to a landholder who sold their property which had Miena Cider Gum because they felt too saddened and unable to effect any change which would save this species*’ (Central Highlands, Tasmania, Anon.). A key future challenge then is to understand and manage the impacts of environmental change on both humans and ecosystems.

## Supporting information

S1 Appendix Print version of the online recent ecological change in australia survey. (PDF)

S2 Appendix Supplementary tables and figures. (PDF)

S3 Appendix Detailed summary of the anecdotes within the climate change ecological impact framework. (PDF)

S1 Table **(A-J). Tabulated summary of the anecdotes within the climate change ecological impact framework**.
(XLSX)
