# Supplementary material for: Recent climate-driven ecological change across a continent as perceived through local ecological knowledge
Source: PLoS One. 2019 Nov 22;14(11):e0224625. doi: 10.1371/journal.pone.0224625 (PMC6874335; doi:10.1371/journal.pone.0224625)
Supplement: S1 Appendix — (PDF) [file pone.0224625.s001.pdf]

# Recent climate-driven ecological change across a continent as perceived through local ecological knowledge

Suzanne M. Prober, Nat Raisbeck-Brown, Natasha B. Porter, Kristen J. Williams, Zoe Leviston, Fiona Dickson

*Ecological Monographs*

**S1 Appendix. Print version of the online Recent Ecological Change in Australia survey**

## Welcome to the Recent Ecological Change in Australia Survey

### Project Overview

You are invited to take part in a research study about recent ecological change in Australia and how climate (and other factors) may have contributed to those ecological changes. You will be asked to choose an area you have known well for greater than 10 years, and to describe the types and potential causes of ecological changes you have seen there. For example, you may have observed trees dying during a prolonged drought.

The study is being carried out by Dr. Suzanne Prober and others from CSIRO Land and Water and the Department of Environment and Energy.

### What does participation involve?

Participation will involve doing a survey that will take about 30 minutes. The survey is in two main parts that cover the following topics:

1. Your views about recent change (or the lack of it) in an area you know well. We will ask you to add information to an on-line map to describe the location of your area. Then we will ask about the kinds of changes you have observed (if any).

2. Your detailed observations of a particular event or 'anecdote', including the species or bushland types involved, the time frames over which you observed particular events and whether you would like to upload some photos.

Finally, to help us understand how well the participants in this survey represent the general population, we will ask you a few (optional) questions about yourself (e.g. gender, occupation).

NOTE: This survey can be completed in a single session or you can return to the survey by the end of December 2017[1] to complete or add information. Incomplete surveys will be saved after 3 months of inactivity, or at the end of December 2017.

>>

[1] Originally planned to finish end of August 2017.

# Recent Ecological Change in Australia Project

## Risks and benefits

This study has received ethics approval from the CSIRO Human Research Ethics Committee. There are no foreseeable risks associated with doing this survey and participation is completely voluntary. Your decision whether to participate will not affect your current or future relationship with the researchers or anyone else at CSIRO.

Your information is automatically saved as you progress through the survey. You are free to withdraw from the survey at any stage. If you do not want your information stored please contact Nat.Raisbeck-Brown@csiro.au before the 31st August 2017 and your survey data (and contact details) will be destroyed. If you have any concerns or complaints about the conduct of the research please contact the CSIRO Ethics Office at csshrec@csiro.au or (07) 3833 5693.

## Confidentiality

All information you provide will be treated confidentially. Personal information will be stripped from the survey data (i.e. data will be de-identified) so that extraction of an individual's survey response will not be possible, unless you have agreed to further follow up or have chosen to be acknowledged if your anecdote is published. Data collected in this survey will be reported in such a way that responses cannot be linked to any individuals (unless requested). Any data collected as part of this study will be securely stored as per CSIRO's Record Keeping Procedure.

## How will my information be used?

It is anticipated that the information obtained through this survey will be published and/or presented in a variety of ways. This includes the production of a general report on community perspectives about recent ecological change in Australia as well as scientific journal publications and conference presentations. De-identified, non-sensitive data collected in the survey may be shared with other researchers for advancing research on this topic.

**Do you consent to taking this survey?**

| Agree | Disagree[2] |
|---|---|

[2] Survey will stop if respondent selects 'Disagree'.  Thanking them for their time.

## You are now beginning Part 1 of the 2 survey parts:

Part 1. Your selected area - an overview of changes that have happened in an area you know well.

Part 2. An anecdote from your selected area - a detailed description of a change you observed in your selected area. You will have the opportunity to provide photos and to provide more than one anecdote.

We are just as interested in areas where you did not observe a change as we are in areas where you did observe change. Please continue with the survey even if you did not observe any changes in your selected area

NOTE: You can take a break at any time during this survey. The survey saves your answers automatically. To get back to your survey click on your link again and you will return to the same place you left off, if you are using the same computer you started on.

## Your selected area

Please use the map below to select a natural area that you have known well for some time (at least 10 years) and are still familiar with.

Examples of areas you might select include: a family farm or other part of the countryside that you live in; a nature reserve that you travel or walk through frequently; a bushland area that you help manage or regularly visit, e.g. for bird watching or camping; or a long term research site.

Your area can be large (e.g. the Atherton Tablelands or the Flinders Rangers) or small (e.g. a bush cemetery or an urban bush block), so long as you know it well. Choose only one area, unless there are a number of areas to which similar observations of change (if any) apply. You can repeat the survey for a different area after completing this survey, by clicking on the original link again.

NOTE: If you return to this page after you have moved on, you will not be able to see your area mapped. Your map will have been recorded so no need to draw it again.

**Step 1 – Zoom in and locate your known area on the map using + / - buttons**

**Step 2 – Draw your known area by clicking on the draw your area button**

| If the map is not working please click here |
|---|

## Your selected area

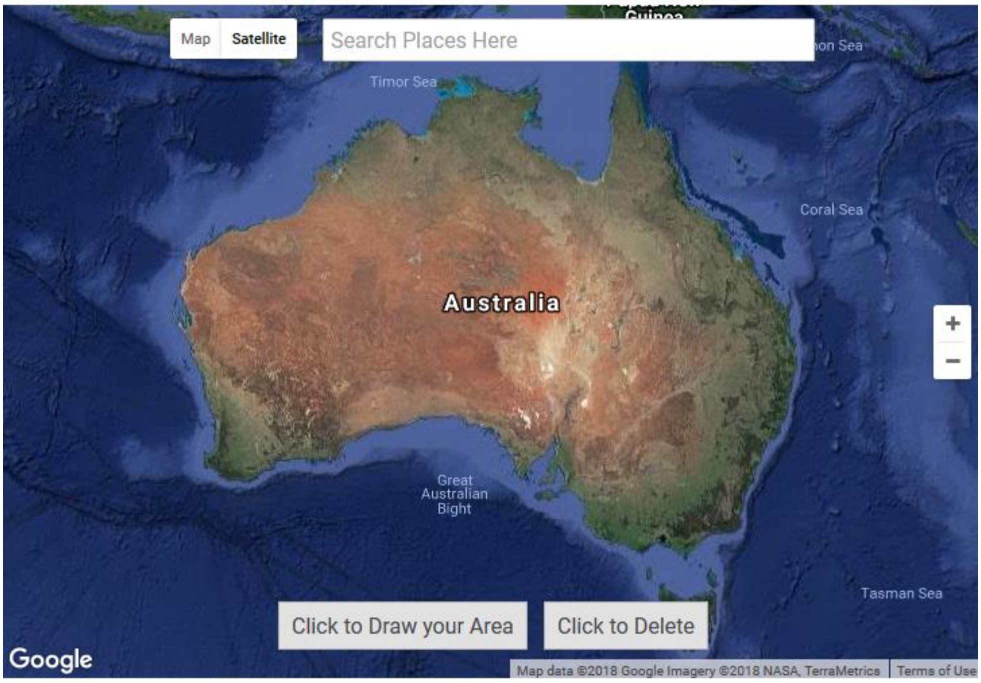


If you have any problems with the mapping tool watch [this video] for a simple tutorial.

Or click here ( [Help me!] ) for more detailed instructions.

>>

*** If respondent does not add an area on the map, they will see this screen when they try to proceed ***

You haven't yet drawn an area on the map. The location of your area is an important part of this survey.

If you would like to return to the map, please use the back arrow at the bottom left.

<< >>

## Your selected area

How would you describe the boundary of this area or place if you had to refer to it?

For example, Karijini National Park; Box-Gum woodlands on the South West Slopes; Mossman Gorge; distance from Orbost on the Bonang Road?

NOTE: You need to add a description of your area here to move onto the rest of the survey.

| |
|---|
| *** Text box will increase in size to accommodate the response recorded *** |

How long have you been familiar with this area?

Please enter number of years as a number in box below.

| |
|---|
| |

Next we are going to ask you a series of questions about recent ecological change in your selected area, based on the list shown below.

NOTE: If you tick 'yes', you will be asked for more information about possible causes and their importance. If you tick 'no', the survey will skip to the next question.

- Birds, butterflies, other insects or migrating animals appearing earlier or later than usual
- Unusual increases or decreases in animal abundance
- Animals dying
- Plants flowering, fruiting, germinating or having growth flushes at unusual times of year
- Unusually high or low levels of shrubs or trees establishing
- Plants dying
- Species disappearing from the area
- Different species arriving in the area
- Unusually high levels of pests or diseases in plants or animals
- Other - please specify ....

## Your selected area:
## birds, butterflies or other animals appearing earlier or later

Q1a[3]. Over the last 10-20 years have you seen: birds, butterflies, other insects or migrating animals appearing earlier or later than you would normally expect?

| Yes | → *Go to Q1b* | No | → *Go to Q2a* |
|---|---|---|---|

Q1b. Did the birds, butterflies, other insects or migrating animals appeared earlier or later than normal?

| Earlier | Later | Both |
|---|---|---|

Q1c. How important do you think the following factors were in causing birds, butterflies, other insects or migrating animals to appear earlier or later than normal? Please select all factors you think are important, and leave blank if you do not think they are important, or you do not know.

| | **Important** | |
|---|---|---|
| Changing timing of flowers or food supply | ☐ | → *If any options selected, go to Q1d* |
| Drought | ☐ | → *If no options selected, go to Q1f* |
| Heatwaves | ☐ | |
| Cold snaps or frosts | ☐ | |
| Warming conditions | ☐ | |
| Other climate factors: ____________________ | ☐ | |

None of the above are important

☐ → *Go to Q1f*

---

[3] Question numbers were not used for the online survey. They are used here to show the skip logic used for the questionnaire.

## Your selected area:
## Birds, butterflies or other animals appearing earlier or later

Q1d. For the climatic or other factor(s) you selected as the cause of birds, butterflies, other insects or migrating animals to appear earlier or later than normal, do you think the factors were part of a normal cycle (e.g. drought years often recorded over the last century), unprecedented, and/or related to a change in climate?

| | **Normal cycle**[4] | **Unprecedented event** | **Related to climate change** |
|---|---|---|---|
| Changing timing of flowers or food supply[5] | ☐ | ☐ | ☐ |
| Drought | ☐ | ☐ | ☐ |
| Heatwaves | ☐ | ☐ | ☐ |
| Cold snaps or frosts | ☐ | ☐ | ☐ |
| Warming conditions | ☐ | ☐ | ☐ |
| Other climate factors: ________________ | ☐ | ☐ | ☐ |

*** If Normal cycle selected for all available options → Go to Q1f; if Unprecedented event or Related to climate change selected for any available options → Go to Q1e ***

Q1e. For any items you checked as Unprecedented or Related to Climate Change, please add a brief comment here if you are able to indicate more about the nature/direction of the cause (e.g. more droughts; fewer droughts; more hot fires; fewer hot fires).

| |
|---|
| *** Text box will increase in size to accommodate the response recorded *** |

Q1f. Please tell us more about these observations of change.

How severe or notable was this change?

| | | |
|---|---|---|
| Mild | Moderate | Major |

Q1g. To what extent are the birds, butterflies, other insects or migrating animals still appearing earlier or later than usual?

| | | |
|---|---|---|
| Not at all | A little bit | A lot |

---

[4] More than one response can be selected for each available option.
[5] Only options selected in Q1c will show as available in this question.

## Your selected area:
## unusual increases or decreases in animal abundance

Q2a. Over the last 10-20 years have you seen: unusual increases or decreases in the abundance of animals?

| Yes | → *Go to Q2b* | No | → *Go to Q3a* |
|---|---|---|---|

Q2b. Did the animals increase or decrease in abundance?

| Increase | Decrease | Both |
|---|---|---|

*** If Increase selected → Go to Q2c (Q2d will not be shown); if Decrease selected → Go to Q2d; if Both selected → Go to Q2c (Q2d will also show below Q2c) ***

Q2c. Are the animals that are increasing in abundance native or non-native to Australia?

| Native | Non-native | Both | Don't know |
|---|---|---|---|

Q2d. Are the animals that are decreasing in abundance native or non-native to Australia?

| Native | Non-native | Both | Don't know |
|---|---|---|---|

## Your selected area:
## unusual increases or decreases in animal abundance

Q2e. How important do you think the following land use factors were in causing the change in animal abundance? Please select all factors you think are important, and leave blank if you do not think they are important, or you do not know.

| | **Important** |
|---|---|
| Legacies of past clearing or other land use, such as:<br>- Habitat fragmentation<br>- Rural/paddock tree decline/dieback | ☐ |
| Recent development such as:<br>- Urbanisation<br>- Road building or widening<br>- Mining or gas developments | ☐ |
| Recent INCREASE in the intensity of agriculture or forestry such as:<br>- Clearing for new crops or pastures<br>- Introduction of irrigation<br>- Increased livestock grazing intensity<br>- Fertilization<br>- Logging | ☐ |
| Recent DECREASE in the intensity of agriculture or forestry such as:<br>- Abandoned croplands<br>- Decreased grazing by livestock | ☐ |
| Other land use factors: ________________ | ☐ |

None of the above are important

☐

## Your selected area:
## unusual increases or decreases in animal abundance

Q2f. How important do you think the following climate and other factors were in causing the change in animal abundance? Please select all factors you think are important, and leave blank if you do not think they are important, or you do not know.

| | **Important** | |
|---|---|---|
| Recent invasion or outbreaks of weeds, pests or diseases | ☐ | *→ If any options selected, go to Q2g* |
| Hot fires | ☐ | *→ If no options selected, go to Q2i* |
| Drought | ☐ | |
| Heatwaves | ☐ | |
| Cold snaps or frosts | ☐ | |
| Storms or cyclones | ☐ | |
| Flooding or heavy rain | ☐ | |
| Warming conditions | ☐ | |
| Other climate factors: ________________ | ☐ | |

None of the above are important

☐ *→ Go to Q2i*

## Your selected area:
## unusual increases or decreases in animal abundance

Q2g. For the climatic or other factor(s) you selected as the cause of changes in animal abundance, do you think the factors were part of a normal cycle (e.g. drought years often recorded over the last century), unprecedented, and/or related to a change in climate?

| | Normal cycle[6] | Unprecedented event | Related to climate change |
|---|---|---|---|
| Recent invasion or outbreaks of weeds, pests or diseases[7] | ☐ | ☐ | ☐ |
| Hot fires | ☐ | ☐ | ☐ |
| Drought | ☐ | ☐ | ☐ |
| Heatwaves | ☐ | ☐ | ☐ |
| Cold snaps or frosts | ☐ | ☐ | ☐ |
| Storms or cyclones | ☐ | ☐ | ☐ |
| Flooding or heavy rain | ☐ | ☐ | ☐ |
| Warming conditions | ☐ | ☐ | ☐ |
| Other climate factors: ________________ | ☐ | ☐ | ☐ |

*** If Normal cycle selected for all available options → Go to Q2i; if Unprecedented event or Related to climate change selected for any available options → Go to Q2h ***

Q2h. For any items you checked as Unprecedented or Related to Climate Change, please add a brief comment here if you are able to indicate more about the nature/direction of the cause (e.g. more droughts; fewer droughts; more hot fires; fewer hot fires).

| |
|---|
| *** Text box will increase in size to accommodate the response recorded *** |

[6] More than one response can be selected for each available option.
[7] Only options selected in Q2f will show as available in this question.

## Your selected area:
## unusual increases or decreases in animal abundance

Q2i. Please tell us more about these observations of change.

How severe or notable was this change?

| Mild | Moderate | Major |
|---|---|---|

Q2j. Are you observing any return to the pre-change condition?

| Not at all | A little bit | A lot |
|---|---|---|

## Your selected area:
## animals dying

Q3a. Over the last 10-20 years have you seen animals dying more than you'd normally expect?

| Yes | → *Go to Q3b* | No | → *Go to Q4a* |
|---|---|---|---|

Q3b. Are the animals that are dying native or non-native to Australia?

| Native | Non-native | Both | Don't know |
|---|---|---|---|

Q3c. How important do you think the following land use factors were in causing animal deaths? Please select all factors you think are important, and leave blank if you do not think they are important, or you do not know.

| | **Important** |
|---|---|
| Legacies of past clearing or other land use, such as:<br>- Habitat fragmentation<br>- Rural/paddock tree decline/ dieback | ☐ |
| Recent development such as:<br>- Urbanisation<br>- Road building or widening<br>- Mining or gas developments | ☐ |
| Recent INCREASE in the intensity of agriculture or forestry such as:<br>- Clearing for new crops or pastures<br>- Introduction of irrigation<br>- Increased livestock grazing intensity<br>- Logging | ☐ |
| Other land use factors: ____________________ | ☐ |

None of the above are important

☐

## Your selected area:
## animals dying

Q3d. How important do you think the following climate and other factors were in causing these animal deaths? Please select all factors you think are important, and leave blank if you do not think they are important, or you do not know.

| | **Important** | |
|---|---|---|
| Recent invasion or outbreaks of weeds, pests or diseases | ☐ | → *If any options selected, go to Q3e* |
| Hot fires | ☐ | → *If no options selected, go to Q3g* |
| Drought | ☐ | |
| Heatwaves | ☐ | |
| Cold snaps or frosts | ☐ | |
| Storms or cyclones | ☐ | |
| Flooding or heavy rain | ☐ | |
| Warming conditions | ☐ | |
| Other climate factors: ____________________ | ☐ | |

None of the above are important

☐ → *Go to Q3g*

## Your selected area:
## animals dying

Q3e. For the climatic or other factor(s) you selected as generating animal deaths, do you think the factors were part of a normal cycle (e.g. drought years often recorded over the last century), unprecedented, and/or related to a change in climate?

| | Normal cycle[8] | Unprecedented event | Related to climate change |
|---|---|---|---|
| Recent invasion or outbreaks of weeds, pests or diseases[9] | ☐ | ☐ | ☐ |
| Hot fires | ☐ | ☐ | ☐ |
| Drought | ☐ | ☐ | ☐ |
| Heatwaves | ☐ | ☐ | ☐ |
| Cold snaps or frosts | ☐ | ☐ | ☐ |
| Storms or cyclones | ☐ | ☐ | ☐ |
| Flooding or heavy rain | ☐ | ☐ | ☐ |
| Warming conditions | ☐ | ☐ | ☐ |
| Other climate factors: ________________ | ☐ | ☐ | ☐ |

*** If Normal cycle selected for all available options → Go to Q3g; if Unprecedented event or Related to climate change selected for any available options → Go to Q3f ***

Q3f. For any items you checked as Unprecedented or Related to Climate Change, please add a brief comment here if you are able to indicate more about the nature/direction of the cause (e.g. more droughts; fewer droughts; more hot fires; fewer hot fires).

| |
|---|
| *** Text box will increase in size to accommodate the response recorded *** |

[8] More than one response can be selected for each available option.
[9] Only options selected in Q3d will show as available in this question.

## Your selected area:
## animals dying

Q3g. Please tell us more about these observations of change.

How severe or notable was this change?

| Mild | Moderate | Major |
|---|---|---|

Q3h. To what extent are these animal deaths still occurring?

| Not at all | A little bit | A lot |
|---|---|---|

Q3i. Have you observed any natural recovery of the animal populations?

| No | A little bit | A lot |
|---|---|---|

## Your selected area:
## plants flowering, fruiting or growing at unusual times

Q4a. Over the last 10-20 years have you seen: plants flowering, fruiting, germinating or having growth flushes at unusual or different times of year?

| | | | |
|---|---|---|---|
| Yes | → *Go to Q4b* | No | → *Go to Q5a* |

Q4b. How important do you think the following climatic or other factors were in causing plants to flower, fruit, germinate or have growth flushes at different or unusual times of year? Please select all factors you think are important, and leave blank if you do not think they are important, or you do not know.

| | **Important** | |
|---|---|---|
| Recent invasion or outbreaks of weeds, pests or diseases | ☐ | → *If any options selected, go to Q4c* |
| Hot fires | ☐ | → *If no options selected, go to Q4e* |
| Drought | ☐ | |
| Heatwaves | ☐ | |
| Cold snaps or frosts | ☐ | |
| Storms or cyclones | ☐ | |
| Flooding or heavy rain | ☐ | |
| Warming conditions | ☐ | |
| Other climate factors: ________________ | ☐ | |

None of the above are important

☐ → *Go to Q4e*

## Your selected area:
## plants flowering, fruiting or growing at unusual times

Q4c. For the climatic or other factor(s) you selected as generating ecological change, do you think the factors were part of a normal cycle (e.g. drought years often recorded over the last century), unprecedented, and/or related to a change in climate?

| | | Normal cycle[10] | Unprecedented event | Related to climate change |
|---|---|---|---|---|
| Recent invasion or outbreaks of weeds, pests or diseases[11] | | ☐ | ☐ | ☐ |
| Hot fires | | ☐ | ☐ | ☐ |
| Drought | | ☐ | ☐ | ☐ |
| Heatwaves | | ☐ | ☐ | ☐ |
| Cold snaps or frosts | | ☐ | ☐ | ☐ |
| Storms or cyclones | | ☐ | ☐ | ☐ |
| Flooding or heavy rain | | ☐ | ☐ | ☐ |
| Warming conditions | | ☐ | ☐ | ☐ |
| Other climate factors: __ | ☐ | ☐ | ☐ | |

*** If Normal cycle selected for all available options → Go to Q4e; if Unprecedented event or Related to climate change selected for any available options → Go to Q4d ***

Q4d. For any items you checked as Unprecedented or Related to Climate Change, please add a brief comment here if you are able to indicate more about the nature/direction of the cause (e.g. more droughts; fewer droughts; more hot fires; fewer hot fires).

| |
|---|
| *** Text box will increase in size to accommodate the response recorded *** |

[10] More than one response can be selected for each available option.
[11] Only options selected in Q4b will show as available in this question.

## Your selected area:
## plants flowering, fruiting or growing at unusual times

Q4e. Please tell us more about these observations of change.

How severe or notable was this change?

| Mild | Moderate | Major |
|---|---|---|

Q4f. To what extent are plants still flowering, fruiting, germinating or having growth flushes at unusual or different times of year?

| Not at all | A little bit | A lot |
|---|---|---|

## Your selected area:
## unusually high or low levels of shrubs or trees establishing

Q5a. Over the last 10-20 years have you seen: unusually high or low levels of shrubs or trees establishing (e.g. woody thickening or lack of tree recruitment)?

| Yes | → *Go to Q5b* | No | → *Go to Q6a* |
|---|---|---|---|

Q5b. Was the level of shrubs or trees establishing unusually high or low?

| High | Low | Both |
|---|---|---|

*** If High selected → Go to Q5c (Q5d will not be shown); if Low selected → Go to Q5d; if Both selected → Go to Q5c (Q5d will also show below Q5c) ***

Q5c. Are the shrubs or trees with high levels of establishment native or non-native to Australia?

| Native | Non-native | Both | Don't know |
|---|---|---|---|

Q5d. Are the shrubs or trees with low levels of establishment native or non-native to Australia?

| Native | Non-native | Both | Don't know |
|---|---|---|---|

## Your selected area:
## unusually high or low levels of shrubs or trees establishing

Q5e. How important do you think the following land use factors were in causing unusually higher or lower levels of shrubs or trees establishing? Please select all factors you think are important, and leave blank if you do not think they are important, or you do not know.

| | **Important** |
| --- | --- |
| Legacies of past clearing or other land use such as:<br>- Soil degradation<br>- Rural/paddock tree decline/dieback | ☐ |
| Recent DECREASE in the intensity of agriculture or forestry such as:<br>- Abandoned croplands<br>- Decreased grazing by livestock | ☐ |
| Other land use factors: ___________________ | ☐ |

None of the above are important

☐

## Your selected area:
## unusually high or low levels of shrubs or trees establishing

Q5f. How important do you think the following climatic or other factors were in causing unusually higher or lower levels of shrubs or trees establishing? Please select all factors you think are important, and leave blank if you do not think they are important, or you do not know.

| | **Important** | |
|---|---|---|
| Recent invasion or outbreaks of weeds, pests or diseases | ☐ | → *If any options selected, go to Q5g* |
| Hot fires | ☐ | → *If no options selected, go to Q5i* |
| Drought | ☐ | |
| Heatwaves | ☐ | |
| Cold snaps or frosts | ☐ | |
| Storms or cyclones | ☐ | |
| Flooding or heavy rain | ☐ | |
| Warming conditions | ☐ | |
| Other climate factors: ____________ | ☐ | |

None of the above are important

☐ → *Go to Q5i*

## Your selected area:
## unusually high or low levels of shrubs or trees establishing

Q5g. For the climatic or other factor(s) you selected as causing unusually higher or lower levels of shrubs or trees establishing, do you think the factors were part of a normal cycle (e.g. drought years often recorded over the last century), unprecedented, and/or related to a change in climate?

| | **Normal cycle**[12] | **Unprecedented event** | **Related to climate change** |
|---|---|---|---|
| Recent invasion or outbreaks of weeds, pests or diseases[13] | ☐ | ☐ | ☐ |
| Hot fires | ☐ | ☐ | ☐ |
| Drought | ☐ | ☐ | ☐ |
| Heatwaves | ☐ | ☐ | ☐ |
| Cold snaps or frosts | ☐ | ☐ | ☐ |
| Storms or cyclones | ☐ | ☐ | ☐ |
| Flooding or heavy rain | ☐ | ☐ | ☐ |
| Warming conditions | ☐ | ☐ | ☐ |
| Other climate factors: ____________ | ☐ | ☐ | ☐ |

*** If Normal cycle selected for all available options → Go to Q5i; if Unprecedented event or Related to climate change selected for any available options → Go to Q5h ***

Q5h. For any items you checked as Unprecedented or Related to Climate Change, please add a brief comment here if you are able to indicate more about the nature/direction of the cause (e.g. more droughts; fewer droughts; more hot fires; fewer hot fires).

| |
|---|
| |

[12] More than one response can be selected for each available option.
[13] Only options selected in Q5f will show as available in this question.

## Your selected area:
## unusually high or low levels of shrubs or trees establishing

Q5i. Please tell us more about these observations of change.

How severe or notable was this change?

| Mild | Moderate | Major |
|---|---|---|

Q5j. To what extent are these higher or lower levels of shrub and tree establishment still happening?

| Not at all | A little bit | A lot |
|---|---|---|

Q5k. Are you observing ongoing survival of the newly established shrubs or trees?

| No most have died | Some have survived | A lot have survived | Don’t know |
|---|---|---|---|

## Your selected area:
## plants dying

Q6a. Over the last 10-20 years have you seen: plants dying more than you'd normally expect?

| Yes | → *Go to Q6b* | No | → *Go to Q7a* |
|---|---|---|---|

Q6b. Are the dying plants native or non-native to Australia?

| Native | Non-native | Both | Don't know |
|---|---|---|---|

Q6c. How important do you think the following land use factors were in causing these plant deaths? Please select all factors you think are important, and leave blank if you do not think they are important, or you do not know.

| | **Important** |
|---|---|
| Legacies of past clearing or other land use, such as:<br>- Soil degradation<br>- Rural/paddock tree decline/ dieback | ☐ |
| Recent development such as:<br>- Urbanisation<br>- Road building or widening<br>- Mining or gas developments | ☐ |
| Recent INCREASE in the intensity of agriculture or forestry such as:<br>- Clearing for new crops or pastures<br>- Introduction of irrigation<br>- Increased livestock grazing intensity in recent decades<br>- Fertilisation<br>- Logging | ☐ |
| Other land use factors: ____________________ | ☐ |

None of the above are important

☐

## Your selected area:
## plants dying

Q6d. How important do you think the following climatic or other factors were in causing these plant deaths? Please select all factors you think are important, and leave blank if you do not think they are important, or you do not know.

| | **Important** | |
|---|---|---|
| Recent invasion or outbreaks of weeds, pests or diseases | ☐ | → *If any options selected, go to Q6e* |
| Hot fires | ☐ | → *If no options selected, go to Q6g* |
| Drought | ☐ | |
| Heatwaves | ☐ | |
| Cold snaps or frosts | ☐ | |
| Storms or cyclones | ☐ | |
| Flooding or heavy rain | ☐ | |
| Warming conditions | ☐ | |
| Other climate factors: ________________ | ☐ | |

None of the above are important

☐ → *Go to Q6g*

## Your selected area: plants dying

Q6e. For the climatic or other factor(s) you selected as generating plant deaths, do you think the factors were part of a normal cycle (e.g. drought years often recorded over the last century), unprecedented, and/or related to a change in climate?

| | Normal cycle[14] | Unprecedented event | Related to climate change |
|---|---|---|---|
| Recent invasion or outbreaks of weeds, pests or diseases[15] | ☐ | ☐ | ☐ |
| Hot fires | ☐ | ☐ | ☐ |
| Drought | ☐ | ☐ | ☐ |
| Heatwaves | ☐ | ☐ | ☐ |
| Cold snaps or frosts | ☐ | ☐ | ☐ |
| Storms or cyclones | ☐ | ☐ | ☐ |
| Flooding or heavy rain | ☐ | ☐ | ☐ |
| Warming conditions | ☐ | ☐ | ☐ |
| Other climate factors: ________________ | ☐ | ☐ | ☐ |

*** If Normal cycle selected for all available options → Go to Q6g; if Unprecedented event or Related to climate change selected for any available options → Go to Q6f ***

Q6f. For any items you checked as Unprecedented or Related to Climate Change, please add a brief comment here if you are able to indicate more about the nature/direction of the cause (e.g. more droughts; fewer droughts; more hot fires; fewer hot fires).

| |
|---|
| *** Text box will increase in size to accommodate the response recorded *** |

[14] More than one response can be selected for each available option.
[15] Only options selected in Q6d will show as available in this question.

## Your selected area:
## plants dying

Q6g. Please tell us more about these observations of change.

How severe or notable was this change?

| Mild | Moderate | Major |
|---|---|---|

Q6h. To what extent are these plant deaths still occurring?

| Not at all | A little bit | A lot |
|---|---|---|

Q6i. Are you observing any regeneration of the plants?

| No | A little bit | A lot |
|---|---|---|

## Your selected area:
## species disappearing from the area

Q7a. Over the last 10-20 years have you seen: species disappearing from the area?

| Yes | → *Go to Q7b* | No | → *Go to Q8a* |
|---|---|---|---|

Q7b. Are the disappearing species native or non-native to Australia?

| Native | Non-native | Both | Don't know |
|---|---|---|---|

Q7c. How important do you think the following land use factors were in causing species to disappear from the area? Please select all factors you think are important, and leave blank if you do not think they are important, or you do not know.

| | **Important** |
|---|---|
| Legacies of past clearing or other land use, such as:<br>- Habitat fragmentation<br>- Soil degradation<br>- Rural/paddock tree decline/dieback | ☐ |
| Recent development such as:<br>- Urbanisation<br>- Road building or widening<br>- Mining or gas developments | ☐ |
| Recent INCREASE in the intensity of agriculture or forestry such as:<br>- Clearing for new crops or pastures<br>- Introduction of irrigation<br>- Increased livestock grazing intensity<br>- Fertilization<br>- Logging | ☐ |
| Recent DECREASE in the intensity of agriculture or forestry such as:<br>- Abandoned croplands<br>- Decreased grazing by livestock | ☐ |
| Other land use factors: ________________ | ☐ |

None of the above are important

☐

## Your selected area:
## species disappearing from the area

Q7d. How important <u>do you think</u> the following climatic or other factors were in causing species to disappear from the area? Please select all factors you think are important, and leave blank if you do not think they are important, or you do not know.

| | **Important** | |
|---|---|---|
| Recent invasion or outbreaks of weeds, pests or diseases | ☐ | → *If any options selected, go to Q7e* |
| Hot fires | ☐ | → *If no options selected, go to Q7g* |
| Drought | ☐ | |
| Heatwaves | ☐ | |
| Cold snaps or frosts | ☐ | |
| Storms or cyclones | ☐ | |
| Flooding or heavy rain | ☐ | |
| Warming conditions | ☐ | |
| Other climate factors: ________________ | ☐ | |

None of the above are important

☐ → *Go to Q7g*

## Your selected area:
## species disappearing from the area

Q7e. For the climatic or other factor(s) you selected as the cause of species disappearing from the area, do you think the factors were part of a normal cycle (e.g. drought years often recorded over the last century), unprecedented, and/or related to a change in climate?

| | Normal cycle[16] | Unprecedented event | Related to climate change |
|---|---|---|---|
| Recent invasion or outbreaks of weeds, pests or diseases[17] | ☐ | ☐ | ☐ |
| Hot fires | ☐ | ☐ | ☐ |
| Drought | ☐ | ☐ | ☐ |
| Heatwaves | ☐ | ☐ | ☐ |
| Cold snaps or frosts | ☐ | ☐ | ☐ |
| Storms or cyclones | ☐ | ☐ | ☐ |
| Flooding or heavy rain | ☐ | ☐ | ☐ |
| Warming conditions | ☐ | ☐ | ☐ |
| Other climate factors: ________________ | ☐ | ☐ | ☐ |

*** If Normal cycle selected for all available options → Go to Q7g; if Unprecedented event or Related to climate change selected for any available options → Go to Q7f ***

Q7f. For any items you checked as Unprecedented or Related to Climate Change, please add a brief comment here if you are able to indicate more about the nature/direction of the cause (e.g. more droughts; fewer droughts; more hot fires; fewer hot fires).

| |
|---|
| *** Text box will increase in size to accommodate the response recorded *** |

[16] More than one response can be selected for each available option.
[17] Only options selected in Q7d will show as available in this question.

## Your selected area:
## species disappearing from the area

Q7g. Please tell us more about these observations of change.

How severe or notable was this change?

| Mild | Moderate | Major |
|---|---|---|

Q7h. Are you observing any return of these species to the area?

| No | A little bit | A lot |
|---|---|---|

## Your selected area:
## different species arriving in the area

Q8a. Over the last 10-20 years have you seen: different species arriving in the area?

| Yes | → *Go to Q8b* | No | → *Go to Q9a* |
|---|---|---|---|

Q8b. Are the newly arriving species native or non-native to Australia?

| Native | Non-native | Both | Don't know |
|---|---|---|---|

Q8c. How important do you think the following land use factors were in causing the arrival of different species in the area? Please select all factors you think are important, and leave blank if you do not think they are important, or you do not know.

| | **Important** |
|---|---|
| Recent development such as:<br>- New roads (access roads)<br>- Mining or gas developments including exploration and access roading<br>- Urbanisation | ☐ |
| Recent INCREASE in the intensity of agriculture or forestry creating new habitats, such as:<br>- Introduction of irrigation<br>- Logging<br>- Increased livestock grazing intensity | ☐ |
| Recent DECREASE in the intensity of agriculture or forestry such as:<br>- Abandoned croplands<br>- Decreased grazing by livestock | ☐ |
| Spread of invasive exotic species (e.g. weeds, ferals) into the area | ☐ |
| Other land use factors: ____________ | ☐ |

None of the above are important

☐

## Your selected area:
## different species arriving in the area

Q8d. How important do you think the following climatic or other factors were in causing the arrival of different species in the area (e.g. through habitats becoming more amenable, or less suitable for other species)?  Please select all factors you think are important, and leave blank if you do not think they are important, or you do not know.

| | **Important** | |
|---|---|---|
| Recent invasion or outbreaks of weeds, pests or diseases | ☐ | *→ If any options selected, go to Q8e* |
| Hot fires | ☐ | *→ If no options selected, go to Q8g* |
| Drought | ☐ | |
| Heatwaves | ☐ | |
| Cold snaps or frosts | ☐ | |
| Storms or cyclones | ☐ | |
| Flooding or heavy rain | ☐ | |
| Warming conditions | ☐ | |
| Other climate factors: ________________ | ☐ | |

None of the above are important

☐ *→ Go to Q8g*

## Your selected area:
## different species arriving in the area

Q8e. For the climatic or other factor(s) you selected as the cause of different species arriving in the area, do you think the factors were part of a normal cycle (e.g. drought years often recorded over the last century), unprecedented, and/or related to a change in climate?

| | Normal cycle[18] | Unprecedented event | Related to climate change |
|---|---|---|---|
| Recent invasion or outbreaks of weeds, pests or diseases[19] | ☐ | ☐ | ☐ |
| Hot fires | ☐ | ☐ | ☐ |
| Drought | ☐ | ☐ | ☐ |
| Heatwaves | ☐ | ☐ | ☐ |
| Cold snaps or frosts | ☐ | ☐ | ☐ |
| Storms or cyclones | ☐ | ☐ | ☐ |
| Flooding or heavy rain | ☐ | ☐ | ☐ |
| Warming conditions | ☐ | ☐ | ☐ |
| Other climate factors: ________________ | ☐ | ☐ | ☐ |

*** If Normal cycle selected for all available options → Go to Q8g; if Unprecedented event or Related to climate change selected for any available options → Go to Q8f ***

Q8f. For any items you checked as Unprecedented or Related to Climate Change, please add a brief comment here if you are able to indicate more about the nature/direction of the cause (e.g. more droughts; fewer droughts; more hot fires; fewer hot fires).

| |
|---|
| *** Text box will increase in size to accommodate the response recorded *** |

[18] More than one response can be selected for each available option.
[19] Only options selected in Q8d will show as available in this question.

## Your selected area:
## different species arriving in the area

Q8g. Please tell us more about these observations of change.

How severe or notable was this change?

| | | |
|---|---|---|
| Mild | Moderate | Major |

Q8h. To what extent have these species remained in the area?

| | | |
|---|---|---|
| Not at all | A little bit | A lot |

## Your selected area:
## unusually high or low levels of pests or diseases

Q9a. Over the last 10-20 years have you seen: unusually higher or lower levels of pests or diseases in plants or animals?

| Yes | → *Go to Q9b* | No | → *Go to Q10a* |
|---|---|---|---|

Q9b. Were the levels of pests or diseases unusually high or low?

| High | Low | Both |
|---|---|---|

*** If High selected → Go to Q9c (Q9d will not be shown); if Low selected → Go to Q9d; if Both selected → Go to Q9c (Q9d will also show below Q9c) ***

Q9c. Are the pests or diseases occurring at unusually high levels native or non-native to Australia?

| Native | Non-native | Both | Don't know |
|---|---|---|---|

*** All respondents from Q9c → Go to A9e ***

Q9d. Are the pests or diseases occurring at unusually low levels native or non-native to Australia?

| Native | Non-native | Both | Don't know |
|---|---|---|---|

*** All respondents from Q9d → Go to Q9f ***

Q9e. Are the plants or animals affected by the high levels of pests or diseases native or non-native to Australia?

| Native | Non-native | Both | Don't know |
|---|---|---|---|

## Your selected area:
## unusually high or low levels of pests or diseases

Q9f. Are the plants or animals affected by the low levels of pests or diseases native or non-native to Australia?

| Native | Non-native | Both | Don't know |
|---|---|---|---|

Q9g. How important do you think the following land use factors were in causing unusually higher or lower levels of pests or diseases? Please select all factors you think are important, and leave blank if you do not think they are important, or you do not know.

| | **Important** |
|---|---|
| Legacies of past clearing or other land use, such as:<br>- Habitat fragmentation<br>- Soil degradation<br>- Rural/paddock tree decline/dieback | ☐ |
| Recent development such as:<br>- Urbanisation<br>- Road building or widening<br>- Mining or gas developments | ☐ |
| Recent INCREASE in the intensity of agriculture or forestry such as:<br>- Introduction of irrigation<br>- Increased livestock grazing intensity<br>- Fertilisation<br>- Logging | ☐ |
| Recent DECREASE in the intensity of agriculture or forestry such as:<br>- Abandoned croplands<br>- Decreased grazing by livestock | ☐ |
| Other land use factors: ___________________ | ☐ |

None of the above are important

☐

## Your selected area:
## unusually high or low levels of pests or diseases

Q9h. How important do you think the following climatic or other factors were in causing unusually higher or lower levels of pests or diseases? Please select all factors you think are important, and leave blank if you do not think they are important, or you do not know.

| | Important | |
|---|---|---|
| Hot fires | ☐ | → *If any options selected, go to Q9i* |
| Drought | ☐ | → *If no options selected, go to Q9k* |
| Heatwaves | ☐ | |
| Cold snaps or frosts | ☐ | |
| Storms or cyclones | ☐ | |
| Flooding or heavy rain | ☐ | |
| Warming conditions | ☐ | |
| Other climate factors: ________________ | ☐ | |

None of the above are important

☐ → *Go to Q9k*

## Your selected area:
## unusually high or low levels of pests or diseases

Q9i. For the climatic or other factor(s) you selected as the cause of unusually higher or lower levels of pests or diseases, do you think the factors were part of a normal cycle (e.g. drought years often recorded over the last century), unprecedented, and/or related to a change in climate?

| | **Normal cycle**[20] | **Unprecedented event** | **Related to climate change** |
|---|---|---|---|
| Hot fires[21] | ☐ | ☐ | ☐ |
| Drought | ☐ | ☐ | ☐ |
| Heatwaves | ☐ | ☐ | ☐ |
| Cold snaps or frosts | ☐ | ☐ | ☐ |
| Storms or cyclones | ☐ | ☐ | ☐ |
| Flooding or heavy rain | ☐ | ☐ | ☐ |
| Warming conditions | ☐ | ☐ | ☐ |
| Other climate factors: ____________ | ☐ | ☐ | ☐ |

*** If Normal cycle selected for all available options → Go to Q9k; if Unprecedented event or Related to climate change selected for any available options → Go to Q9j ***

Q9j. For any items you checked as Unprecedented or Related to Climate Change, please add a brief comment here if you are able to indicate more about the nature/direction of the cause (e.g. more droughts; fewer droughts; more hot fires; fewer hot fires).

| |
|---|
| *** Text box will increase in size to accommodate the response recorded *** |

[20] More than one response can be selected for each available option.
[21] Only options selected in Q9h will show as available in this question.

## Your selected area:
## unusually high or low levels of pests or diseases

Q9k. Please tell us more about these observations of change.

How severe or notable was this change?

Mild | Moderate | Major

Q9l. To what extent are these unusually high or low levels of pests or diseases still occurring?

Not at all | A little bit | A lot

## Your selected area:
## other

Q10a. Over the last 10-20 years have you seen: other ecological changes in the area (e.g. vegetation getting more open and grassy)?

| Yes | → *Go to Q10b* | No | → *Go to Q11a* |
|---|---|---|---|

Q10b. Please specify those other changes:

*** Text box will increase in size to accommodate the response recorded ***

Q10c. How important do you think the following land use factors were in causing the other changes in the area? Please select all factors you think are important, and leave blank if you do not think they are important, or you do not know.

| | **Important** |
|---|---|
| Legacies of past clearing or other land use, such as:<br>- Habitat fragmentation<br>- Soil degradation<br>- Rural/paddock tree decline/dieback | ☐ |
| Recent development such as:<br>- Urbanisation<br>- Road building or widening<br>- Mining or gas developments | ☐ |
| Recent INCREASE in the intensity of agriculture or forestry such as:<br>- Clearing for new crops or pastures<br>- Introduction of irrigation<br>- Increased livestock grazing intensity<br>- Fertilisation<br>- Logging | ☐ |
| Recent DECREASE in the intensity of agriculture or forestry such as:<br>- Abandoned croplands<br>- Decreased grazing by livestock | ☐ |
| Other land use factors: ____________________ | ☐ |

None of the above are important

☐

## Your selected area:
## other

Q10d. How important do you think the following climatic or other factors were in causing the other changes in the area? Please select all factors you think are important, and leave blank if you do not think they are important, or you do not know.

| | Important | |
|---|---|---|
| Recent invasion or outbreaks of weeds, pests or diseases | ☐ | → *If any options selected, go to Q10e* |
| Hot fires | ☐ | → *If no options selected, go to Q10g* |
| Drought | ☐ | |
| Heatwaves | ☐ | |
| Cold snaps or frosts | ☐ | |
| Storms or cyclones | ☐ | |
| Flooding or heavy rain | ☐ | |
| Warming conditions | ☐ | |
| Other climate factors: ________________ | ☐ | |

None of the above are important

☐ → *Go to Q10g*

## Your selected area:
## other

Q10e. For the climatic or other factor(s) you selected as the cause of the other changes in the area, do you think the factors were part of a normal cycle (e.g. drought years often recorded over the last century), unprecedented, and/or related to a change in climate?

| | **Normal cycle**[22] | **Unprecedented event** | **Related to climate change** |
|---|---|---|---|
| Recent invasion or outbreaks of weeds, pests or diseases[23] | ☐ | ☐ | ☐ |
| Hot fires | ☐ | ☐ | ☐ |
| Drought | ☐ | ☐ | ☐ |
| Heatwaves | ☐ | ☐ | ☐ |
| Cold snaps or frosts | ☐ | ☐ | ☐ |
| Storms or cyclones | ☐ | ☐ | ☐ |
| Flooding or heavy rain | ☐ | ☐ | ☐ |
| Warming conditions | ☐ | ☐ | ☐ |
| Other climate factors: ________________ | ☐ | ☐ | ☐ |

*** If Normal cycle selected for all available options → Go to Q10g; if Unprecedented event or Related to climate change selected for any available options → Go to Q10f ***

Q10f. For any items you checked as Unprecedented or Related to Climate Change, please add a brief comment here if you are able to indicate more about the nature/direction of the cause (e.g. more droughts; fewer droughts; more hot fires; fewer hot fires).

| |
|---|
| *** Text box will increase in size to accommodate the response recorded *** |

[22] More than one response can be selected for each available option.
[23] Only options selected in Q10c will show as available in this question.

## Your selected area:
## other

Q10g. Please tell us more about these observations of change.

How severe or notable was this change?

| Mild | Moderate | Major |
|---|---|---|

Q10h. To what extent are these other changes still occurring?

| Not at all | A little bit | A lot |
|---|---|---|

Q10i. Are you observing any return to the pre-change condition?

| No | A little bit | A lot |
|---|---|---|

## An anecdote from your selected area

Q11a. In this section you can describe a detailed anecdote about your selected area.

We are very keen to collect your story towards building a picture of recent ecological change in Australia.

Would you like to share a story about your area?

| Yes | → *Go to Q11b* | No | → *Go to Q14* |
| --- | --- | --- | --- |

Q11b. From the changes you indicated for your area, please choose one change that you considered most strongly linked to climate change.

OR, if you did not observe a change that you believe is linked to climate change, please choose an example from your area related to a different cause (but preferably still climate- or weather-related).

In the text box below, please tell us as much as you can about this change (e.g. detailed location, what happened), as well as what you think is causing this change.

| ***Text box will increase in size to accommodate the size of anecdote recorded*** |
| --- |

For example: *'In about 2008, I noticed the Jarrah trees (Eucalyptus) starting to die in the woodland patch around the granite outcrop on the shallower soils up in my back paddock (32.9 S, 117.0 E). This was after several years of quite severe drought. We've had lots of droughts before, but I haven't seen a drought that long and severe in the 40 years I've been farming here. Most of the trees began to reshoot in subsequent years but eventually the new shoots died off too. Not all of the Jarrah trees have died – just perhaps 30% of them are now dead within the patch of about 5 ha, some big trees and some small. I've noticed similar deaths around my local area, although it is difficult to see a pattern that explains why some Jarrahs have died and others not. I haven't noticed any of the other plants dying off, but I noticed there are wattles coming up around the dead Jarrahs rather than new Jarrah seedlings.'*

## An anecdote from your selected area

Q11c. Please provide a latitude and longitude for a location where you observed this change, as accurately as you can.

Latitude

Longitude

Q11d. Over what period of time did you observe this change (be specific a possible)?

From date:

To date (write ongoing if the process is still happening):

Q11e1. If your anecdote is about a particular species, please tell us the name(s) you use for them.

If you know the Latin name please add below otherwise common name is fine.

Add species individually (up to 20) by clicking on the "Add another species – Yes" button below.

Latin name (Genus species)

Common name

## An anecdote from your selected area

Add another species?

| Yes | → *Go to Q11e2* | No | → *Go to Q11f* |
|---|---|---|---|

*** Q11e2-Q11e20 are repeats of the above question – allowing for up to 20 species to be added by respondents ***

Q11f. If your observation applies more generally to an ecosystem or vegetation type, please describe the ecosystem, including some key species if possible.

What name do you call the affected ecosystem or vegetation type?

(e.g. forest, Jarrah forest, rainforest, cool temperate rainforest, tropical savanna, Eucalypt woodland, Salmon Gum (*Eucalyptus salmonophloia*) woodland, Banksia woodland, Mulga woodland, tussock grassland, spinifex grassland or heathland)

| *** Text box will increase in size to accommodate the anecdote recorded *** |
|---|

Q11g. Do you have any photos of this area that you would like to share? Time series or before-after photos would be especially valuable.

Please attach up to 5 photos here (please use small file sizes – less than 500 kb):

| Yes – I would like to add photos | → *Go to Q11g1* | No – I have no photos | → *Go to Q11h* |
|---|---|---|---|

## An anecdote from your selected area

Q11g1. Photo 1 name or description:

NOTE: Please enter a photo description. This will show you the upload button.

*** Text box will increase in size to accommodate the description recorded ***

Photo 1:

Drop files or click here to upload

Do you want to add another photo?

Yes → *Go to Q11g2* No → *Go to Q11h*

*** Q11g2-Q11g5 are repeats of the above question – allowing for up to 5 photos to be added by respondents ***

Q11h. Other than what you have already mentioned are there other climate or non-climate conditions driving this change?

For example: *'The trees that died are in a healthy woodland, backing on to a nature reserve. They may get some fertiliser blown onto them in some years but otherwise I can't see any other reasons why they have died.'*

*** Text box will increase in size to accommodate the response recorded ***

## An anecdote from your selected area

Q11i. To the best of your knowledge, how many other people do you know who have observed this change?

| 1-3 people | 4-7 people | More than 7 people | No others |
|---|---|---|---|

Q11j. If the change is not ongoing are you observing any return to pre-change conditions?

| No | A little bit | A lot | Not applicable |
|---|---|---|---|

Q11k. Do you think this area has the potential to return to its previous condition?

| No | Yes, without active restoration | Yes, with active restoration |
|---|---|---|

That is all the questions we have about your anecdote for your selected area.

If you would like to review your answers please do so now as beyond this point you cannot get back to this section.

Q12a. Would you like to tell us another story for this area?

Yes → *Go to Q12b*  No → *Go to Q14*

*** Respondents were able to provide a second and third anecdote about their area in the next two sections of the questionnaire. These next two sections are duplications of the previous section "An anecdote from your selected area". Q12b-Q12k relate to a second anecdote, and Q13b-Q13k relate to a third anecdote. Both sections were entitled "Another anecdote from your selected area" ***

## Climate change questions[24]

Thank-you for telling us about your climate change observations. You have nearly completed the survey. We would like to finish with two general questions on your thoughts about ecological change in Australia, followed by a short set of questions about yourself more generally.

Q14. When you think about the overall quality of the natural environment in Australia just now, do you think it is:

| Generally improving | Staying pretty much the same | Generally worsening |
|---|---|---|

Q15. Which of the following statements best describes your thoughts about climate change?

| |
|---|
| I don't think that climate change is happening |
| I have no idea whether climate change is happening |
| I think climate change is happening but it's just a natural fluctuation in Earth's temperature |
| I think climate change is happening/ I think humans are largely causing it |

[24] This heading was not shown in the online survey – it is here to describe this section of questions.

## About you

To help us understand how well the participants in this survey represent the general population, we would like to ask you a few questions about yourself.

Please note all of these questions are voluntary and all information gathered is confidential.

Q16. What are your professional or other interests [e.g. volunteer or recreational] in land management and/or Australian ecosystems, plants, animals or other organisms (tick as many as applicable)?

| | |
|---|---|
| Farmer – add type of farming (optional)[25] ______________________ | Fire manager |
| Grazier – add type of livestock (optional) ______________________ | Forester |
| Viticulturist | Weeds or pests manager |
| Bee keeper/Apiarist | Catchment group/Landcare member |
| Agricultural officer | Indigenous owner |
| Other agricultural worker | Tourist industry |
| Agricultural researcher | Recreational visitor |
| Ecological or biological researcher | Recreational fisher |
| Ecological or biological consultant | Naturalist/observer |
| Commonwealth/State government officer in natural resource management | Bird watcher |
| Local government officer in natural resource management | Photographer |
| Natural resource management agency officer | Driver/traveller |
| Park ranger | Other ______________________ |
| Indigenous land manager (including rangers) | |

[25] This list of options was presented in one column for the online survey (two columns presented here to reduce length of survey).

## About you

Q17. What is your postcode?

Q18. What is your year of birth?

Q19.What is your place of birth?

(Town or region if inside Australia, country if not Australia)

Q20. What is your first language?

Q21. What is your gender?

| Female | Male | Other |
|---|---|---|

Q22. What is your household's gross annual income before tax?

| |
|---|
| Less than $30,000 |
| $30,000 - $59,999 |
| $60,000 - $89,999 |
| $90,000 - $119,999 |
| $120,000 - $149,999 |
| More than $150,000 |

## About you

Q23. What is the highest level of education you have attained?

| |
|---|
| Completed primary school |
| Completed high school Year 10 |
| Completed high school |
| Completed trade/TAFE qualification |
| Completed undergraduate degree |
| Completed postgraduate qualification |

Q24. Would you be willing to participate in further phases of this research, in particular a follow-up telephone call to discuss your anecdotes?

| Yes | → *Go to Q24a* | No | → *Go to Q25* |
|---|---|---|---|

Q24a. Please provide email and contact details below. These details will remain confidential and will not be passed on or made public (unless you choose to be acknowledged if your anecdote is published).

Please enter your telephone number including the area code if a land line

Please enter your email address:

Please provide your name:

## About you

Q25. If you provided photos do you provide permission for them to be published?

| Yes | → *Go to Q25a* | No | → *Go to Q26* | Not applicable | → *Go to Q26* |
|---|---|---|---|---|---|

Q25a. If so, how would you like to be acknowledged for the photo(s)?

| |
|---|
| |

Q26. If your anecdote is published how would you like to be acknowledged?

| |
|---|
| Not at all |
| As previously stated |
| Please add your preferred acknowledgement: ____________________ |

Thank you for your time. You have now completed the survey. Please click the submit button to store your information and exit the survey.

**Submit**
