# Supplementary material for: Recent climate-driven ecological change across a continent as perceived through local ecological knowledge
Source: PLoS One. 2019 Nov 22;14(11):e0224625. doi: 10.1371/journal.pone.0224625 (PMC6874335; doi:10.1371/journal.pone.0224625)
Supplement: S2 Appendix — (PDF) [file pone.0224625.s002.pdf]

Recent climate-driven ecological change across a continent as perceived through local ecological knowledge

Suzanne M. Prober, Nat Raisbeck-Brown, Natasha B. Porter, Kristen J. Williams, Zoe Leviston, Fiona Dickson

S2 Appendix. Supplementary tables and figures

S2 Appendix Table A Household income of respondents

Household Income	# respondents
Less than $30,000	29
$30,000 - $59,999	39
$60,000 - $89,999	34
$90,000 - $119,999	37
$120,000 - $149,999	21
More than $150,000	45
Not indicated	121
Total	326

S2 Appendix Table B Address of respondents

State/Territory	# respondents
ACT	8
NSW	49
NT	6
Qld	50
SA	19
Tas	10
Vic	50
WA	31
Not indicated	103
Total	326

S2 Appendix Table C. Significance (P) of binomial general linear models for individual explanatory variables potentially explaining the proportion of questions for which respondents had observed an ecological change (Yes) and associated this with any driver or a land use driver, climate driver or climate change driver.

	Yes	Yes with Land Use Driver	Yes with Climate Driver	Yes with Climate Change Driver
Climate change belief	**0.035**	0.233	**0.018**	**<0.001**
Environmental belief	**<0.001**	**0.026**	**<0.001**	**<0.001**
Ecological Researcher	**0.002**	0.179	**0.004**	**0.009**
Farmer	0.217	0.518	0.275	0.469
Natural resource manager	0.116	0.337	0.225	0.497
Gender	0.130	0.340	0.085	0.078
Household income	0.194	0.657	0.259	0.495
Year of birth	0.543	0.997	0.068	0.260
Education level	0.181	0.279	0.288	0.629

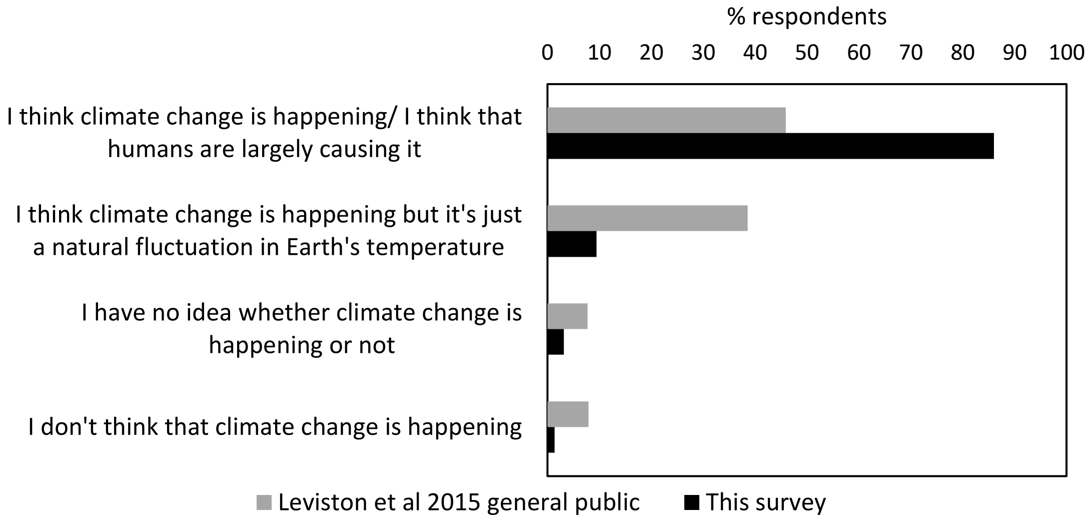

S2 Appendix Fig A Views of respondents in this survey, compared with random members of the general public who were asked the same question in Leviston et al. (2015).

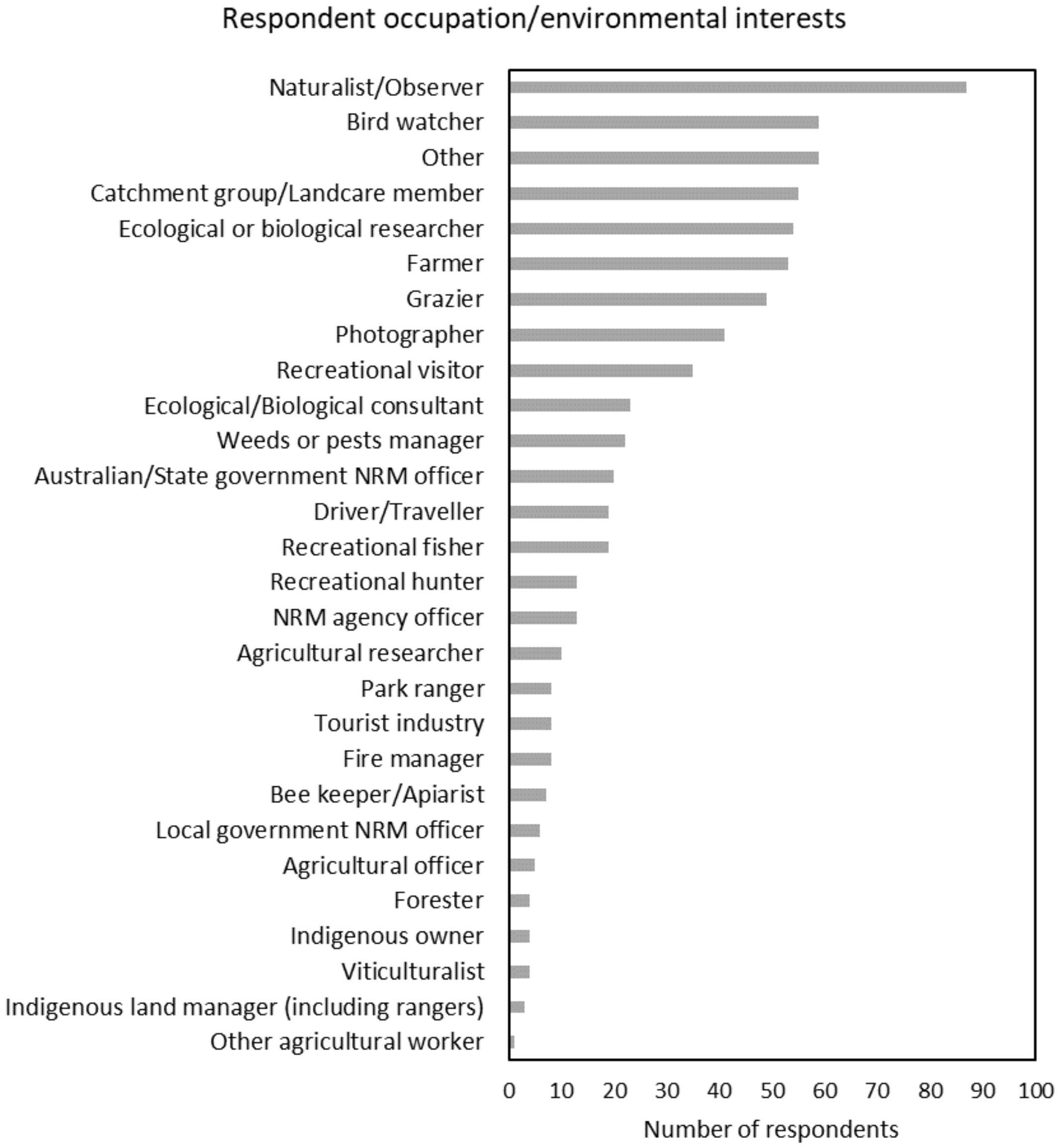

S2 Appendix Fig B. Occupations or environmental interests of the 200 survey respondents who answered this question. Note that respondents on average selected 3.1 categories. NRM, Natural Resource Management.

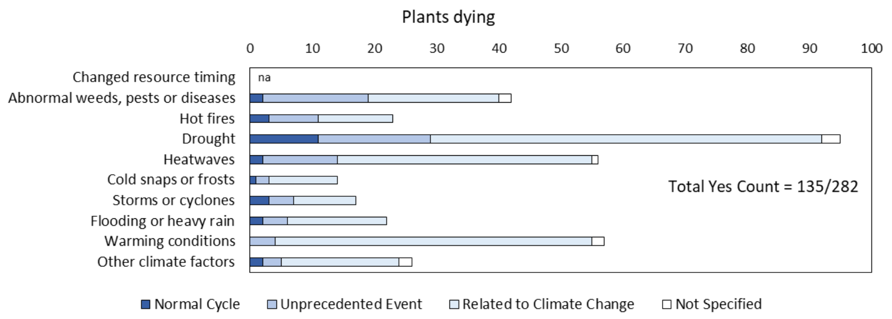
Plants dying
0 10 20 30 40 50 60 70 80 90 100
Changed resource timing
Abnormal weeds, pests or diseases
Hot fires
Drought
Heatwaves
Cold snaps or frosts
Storms or cyclones
Flooding or heavy rain
Warming conditions
Other climate factors
na
Total Yes Count = 135/282
Normal Cycle
Unprecedented Event
Related to Climate Change
Not Specified

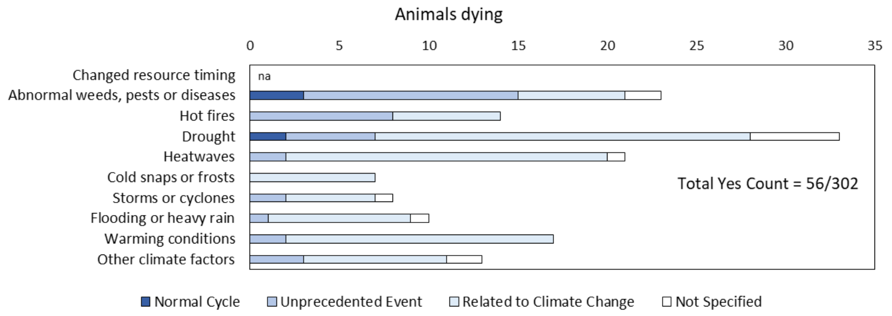
Animals dying
0 5 10 15 20 25 30 35
Changed resource timing
Abnormal weeds, pests or diseases
Hot fires
Drought
Heatwaves
Cold snaps or frosts
Storms or cyclones
Flooding or heavy rain
Warming conditions
Other climate factors
na
Total Yes Count = 56/302
Normal Cycle
Unprecedented Event
Related to Climate Change
Not Specified

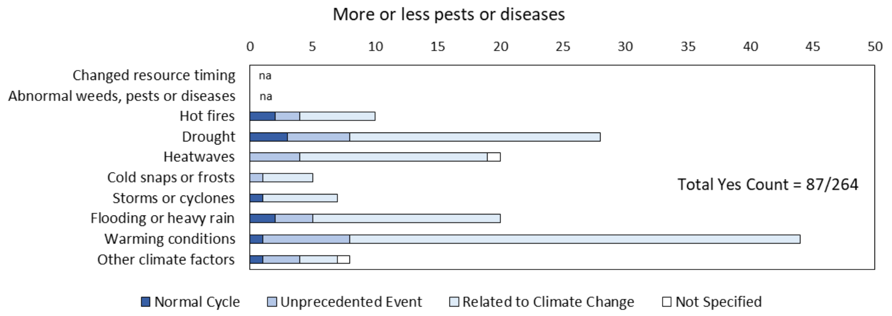
More or less pests or diseases
0 5 10 15 20 25 30 35 40 45 50
Changed resource timing
Abnormal weeds, pests or diseases
Hot fires
Drought
Heatwaves
Cold snaps or frosts
Storms or cyclones
Flooding or heavy rain
Warming conditions
Other climate factors
na
na
Total Yes Count = 87/264
Normal Cycle
Unprecedented Event
Related to Climate Change
Not Specified

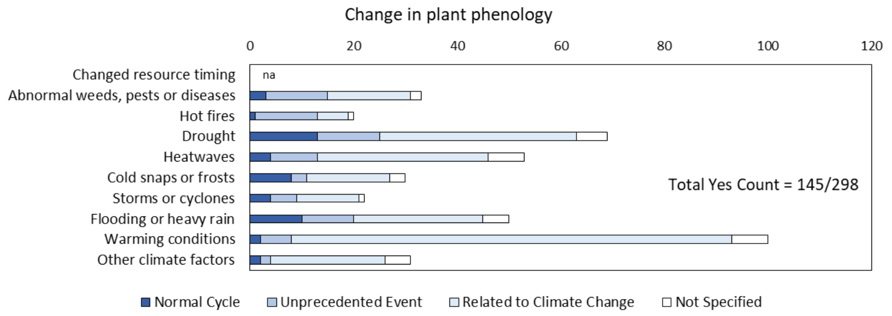
Change in plant phenology
0 20 40 60 80 100 120
Changed resource timing
Abnormal weeds, pests or diseases
Hot fires
Drought
Heatwaves
Cold snaps or frosts
Storms or cyclones
Flooding or heavy rain
Warming conditions
Other climate factors
na
Total Yes Count = 145/298
Normal Cycle
Unprecedented Event
Related to Climate Change
Not Specified

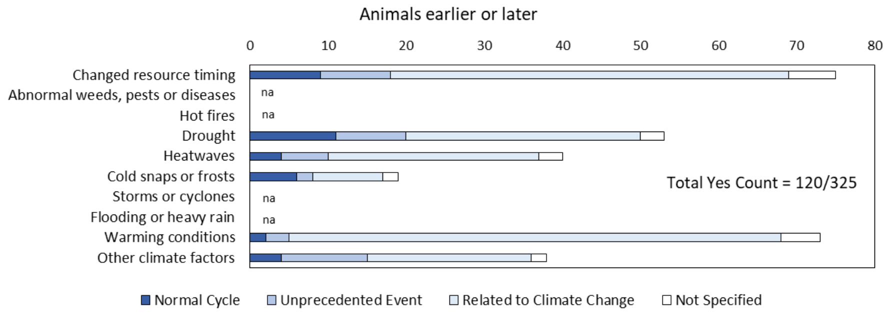
Animals earlier or later
0 10 20 30 40 50 60 70 80
Changed resource timing
Abnormal weeds, pests or diseases
Hot fires
Drought
Heatwaves
Cold snaps or frosts
Storms or cyclones
Flooding or heavy rain
Warming conditions
Other climate factors
na
na
na
na
Total Yes Count = 120/325
Normal Cycle
Unprecedented Event
Related to Climate Change
Not Specified

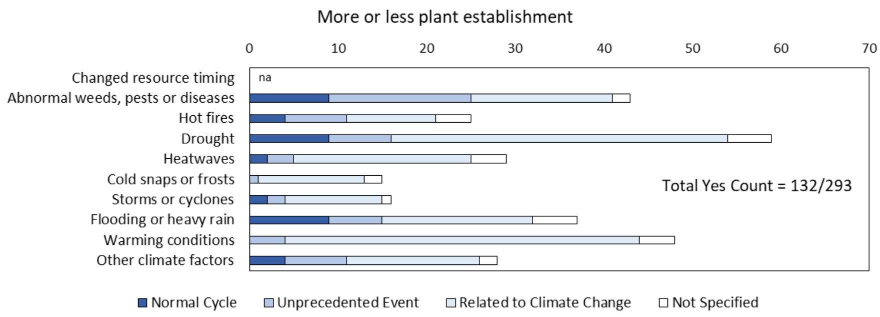
More or less plant establishment
0
10
20
30
40
50
60
70
Changed resource timing
Abnormal weeds, pests or diseases
Hot fires
Drought
Heatwaves
Cold snaps or frosts
Storms or cyclones
Flooding or heavy rain
Warming conditions
Other climate factors
na
Total Yes Count = 132/293
Normal Cycle
Unprecedented Event
Related to Climate Change
Not Specified

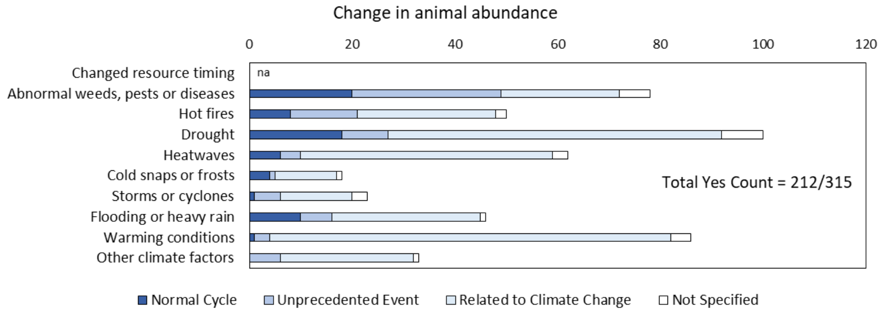
Change in animal abundance
0
20
40
60
80
100
120
Changed resource timing
Abnormal weeds, pests or diseases
Hot fires
Drought
Heatwaves
Cold snaps or frosts
Storms or cyclones
Flooding or heavy rain
Warming conditions
Other climate factors
na
Total Yes Count = 212/315
Normal Cycle
Unprecedented Event
Related to Climate Change
Not Specified

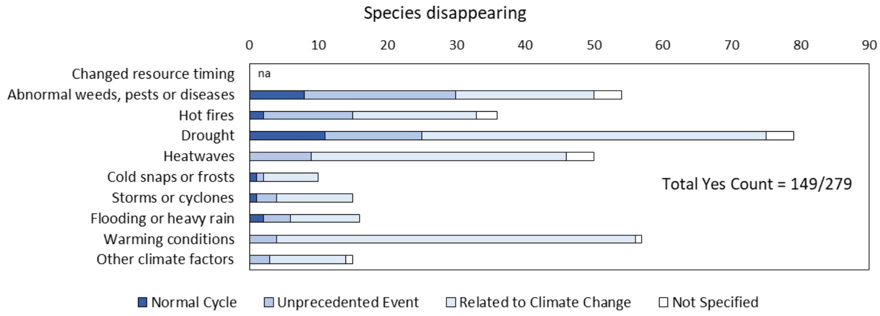
Species disappearing
0
10
20
30
40
50
60
70
80
90
Changed resource timing
Abnormal weeds, pests or diseases
Hot fires
Drought
Heatwaves
Cold snaps or frosts
Storms or cyclones
Flooding or heavy rain
Warming conditions
Other climate factors
na
Total Yes Count = 149/279
Normal Cycle
Unprecedented Event
Related to Climate Change
Not Specified

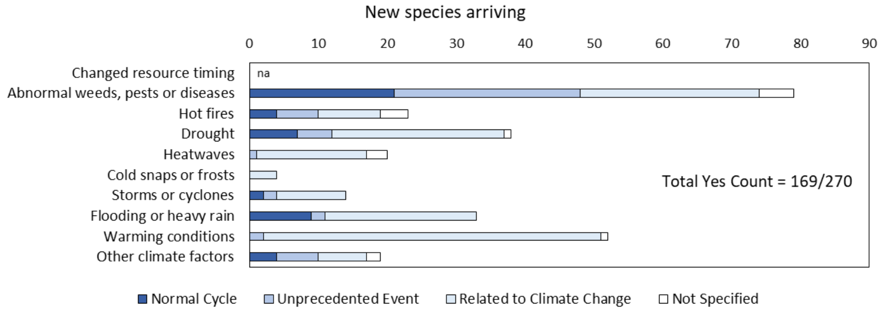
New species arriving
0
10
20
30
40
50
60
70
80
90
Changed resource timing
Abnormal weeds, pests or diseases
Hot fires
Drought
Heatwaves
Cold snaps or frosts
Storms or cyclones
Flooding or heavy rain
Warming conditions
Other climate factors
na
Total Yes Count = 169/270
Normal Cycle
Unprecedented Event
Related to Climate Change
Not Specified

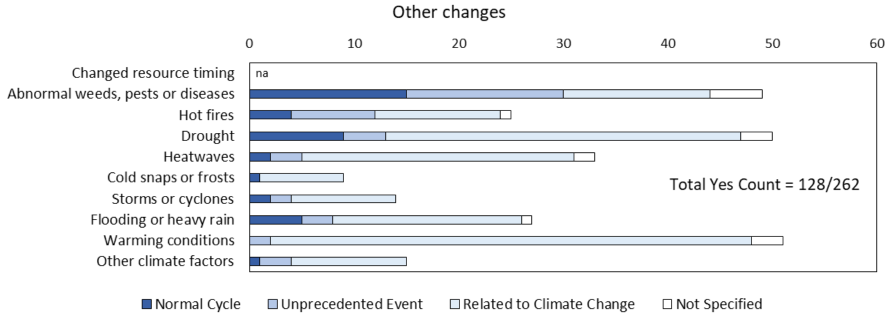
Other changes
0
10
20
30
40
50
60
Changed resource timing
Abnormal weeds, pests or diseases
Hot fires
Drought
Heatwaves
Cold snaps or frosts
Storms or cyclones
Flooding or heavy rain
Warming conditions
Other climate factors
na
Total Yes Count = 128/262
Normal Cycle
Unprecedented Event
Related to Climate Change
Not Specified

S2 Appendix Fig C. Frequency (number out of total yes count) of attribution of different types of weather or climate-related conditions as drivers of each primary change type, and whether these conditions were considered normal, unprecedented or related to climate change. Yes count=number of respondents observing the change (as a subset of total number of respondents for that primary question).

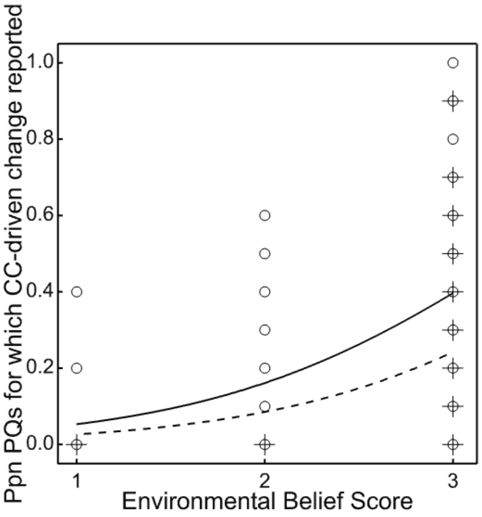

S2 Appendix Fig D. A combination of Environmental Belief and whether the respondent was an ecological researcher (+=ecological researcher, dashed line; o=non-ecological researcher, solid line) resulted in the best model to explain the total number of the ten primary questions (PQs) to which each respondent answered yes, in this case for climate change (CC) drivers only (Adjusted R^2 =0.176, see Table 3 for other drivers).
