# Supplementary material for: Recent climate-driven ecological change across a continent as perceived through local ecological knowledge
Source: PLoS One. 2019 Nov 22;14(11):e0224625. doi: 10.1371/journal.pone.0224625 (PMC6874335; doi:10.1371/journal.pone.0224625)
Supplement: S3 Appendix — (PDF) [file pone.0224625.s003.pdf]

Recent climate-driven ecological change across a continent as perceived through local ecological knowledge

Suzanne M. Prober, Nat Raisbeck-Brown, Natasha B. Porter, Kristen J. Williams, Zoe Leviston, Fiona Dickson

S3 Appendix. Detailed summary of the anecdotes within the climate change ecological impact framework

Introduction

Here we summarize in detail the content of the anecdotes from the Recent Ecological Change in Australia survey. The text is structured from organism to landscape scales (Sections 1-5) according to the ecological impact framework of Bellard et al. (2012) and Scheffers et al. (2016) (adapted for this study in Table 1 main text, Tables A-J in S1 Table). Codes against anecdotes (e.g. 316e) cross reference to Tables A-J in S1 Table (see separate excel file) and to data on the CSIRO Data Access Portal. Attribution of quoted anecdotes is provided using wording requested by the relevant respondents.

1 Organisms

1.1 Organisms: genetics

While genetic adaptation is expected to be an important response of species to climate change (e.g. Sgro et al. 2011), such changes are not readily observable. Consistent with this, only one anecdote described a case of potential genetic change associated with climate change. This case involved plant hybridization in Mullumbimby, north coast New South Wales: *'Regenerati[ng] species are often hybrids with species coming south...e.g. wild (not planted...) Bangalow Palms* [Archontophoenix cunninghamiana] *are often a Bangalow/Alexander palm* [Archontophoenix alexandrae] *hybrid indicating warming'* (316e, Anon.). This anecdote is consistent with observations reported globally of new hybridization zones enabled by changes in species ranges, although most examples to date are for fauna (Scheffers et al. 2016).

1.2 Organisms: physiology: mortality

Individual organisms may respond to climate stressors through plastic responses in phenotypic traits, including both changes in morphology and physiology. For example, plants can adjust to hotter or drier conditions by altering stomatal conductance, water use efficiency or specific leaf area (Nicotra et al. 2010), and animals may acclimatize to higher thermal optima or adjust their body size (Gardner et al. 2014). While morphological change would potentially have been observable by respondents, none of the anecdotes reported this type of change. Rather, effects of physiological stress on individuals were most evident where physiological thresholds had apparently been breached, resulting in die-back, mortality or increased pests and diseases in individuals. Consistent with this, a range of respondents reported mortality events and changing pest and disease loads in plants and animals, purportedly arising from direct or indirect climate stressors.

Plant mortality. Plant species reported to have been dying as a likely or possible consequence of climate change included 31 species of eucalypts (*Eucalyptus* and *Corymbia* spp.) and various species

of *Banksia, (Allo)Casuarina, Avicennia, Acacia, Melaleuca, Hakea,* and *Araucaria*, as well as tree ferns (*Dicksonia/Cyathea* sp.) and (often unspecified) herbaceous plants (Table A in S1 Table). This mortality was sometimes reported as being followed by failed recruitment, for example after the death of *Avicennia marina* (Grey mangrove , 117a), *Banksia marginata* (Silver banksia, 12a) and *Acacia carneorum* (Purple-wood wattle, 124a), although replacement *'by young trees of the same species'* (174a, Peter Haselgrove) was reported in conjunction with (unattributed) death of red gums and *Eucalyptus caliginosa* (Broad-leaved stringybark) in otherwise healthy forests of Storm King in the Darling Downs area, Queensland.

Some of the anecdotes for tree mortality were indicative of deaths at substantial scales. For example a respondent reporting from Harcourt in the Central Highlands of Victoria observed that after many years of drought, *'This past year we have lost a whole VALLEY of large eucalypts and box trees for no apparent reason'* (49b, Anon.), and monitoring in *Melaleuca* woodland of the Princess Charlotte Bay area (of far north Queensland) revealed that by 2016 *'There were tens of thousands of dead trees and in some areas the salt edge had moved inland over 500m'* (27a, Simon Thompson and the Lama Lama Land Trust). Similarly, an anecdote for the Gulf of Carpentaria region of the Northern Territory described that the already well-reported death of mangroves (Duke et al. 2017) was accompanied by unreported *'death of* Eucalyptus tetrodonta [*Darwin Stringybark*] *in extensive areas of the Gulf woodlands/open-forests',* noting that *'the scale of this particular event was unprecedented'* (242a, Anon.). Mortality was not always 100%, for example for mixed eucalypt species at a property in the Strathbogie Ranges, central Victoria, a respondent estimated *'a 30% decline over 30 years, based on a recent survey of standing and fallen trees...I rather feel that this is also a function of long droughts, heat shock and very ferocious wind storms (attributes that are intensified through climate change)* (107a, Garry McDonald).

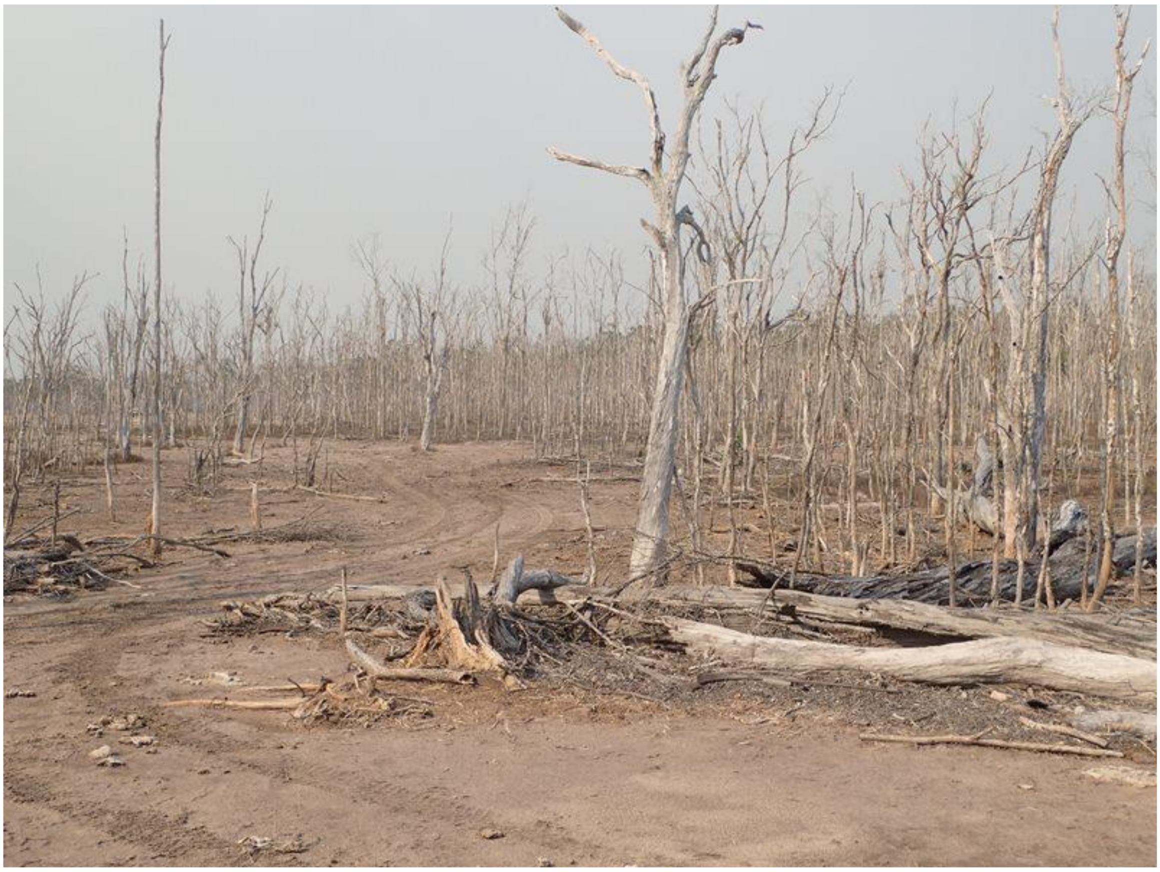

Plate 1 Monitoring in *Melaleuca* woodland of the Princess Charlotte Bay, far north Queensland area revealed that by 2016 '*There were tens of thousands of dead trees and in some areas the salt edge had moved inland over 500m'* (27a). Image credits: Simon Thompson and the Lama Lama Land Trust.

The most common purported drivers in anecdotes describing plant deaths were warming (especially heatwaves) or drying (water stress), and also included salinization associated with sea-level rise (particularly affecting *Melaleuca* spp.) and other extreme weather events (Table A in S1 Table). The latter included floods, storms, heavy snow, severe frosts and rainfall-driven grass-fire cycles. For example, death of emergent old *Hakea lorea* subsp. *lorea* (Long-leaf corkwood) trees was reported as resulting from hot fires on dry hills invaded by *Triodia* spp. (Spinifex) and *Cenchrus pennisetiformis* (Cloncurry buffel grass) after unusually wet years (associated with variation in the northern monsoon, 25a, north-west Queensland).

A number of respondents also reported that climate stressors increased mortality via pests or diseases, including *Banksia integrifolia* (Coast banksia) succumbing to a virus (89a, Hampton/Sandringham, Melbourne area, Victoria), *Banksia robur* (Swamp banksia) succumbing to insect pests after long heatwaves, droughts and heavy storms (326a, Cooloola Cove, Wide Bay-Burnett area, Queensland), fungal parasites (*Phellinus sublamaensis,* White rot) killing stressed saplings in subtropical riverine rainforest (316a, Mullumbimby, north coast New South Wales), and invertebrate herbivory, gingering, canker and *Phytophthora cinnamomi* (Phytophthora root rot) in eucalypts. Other respondents reported interactions between climate and land use drivers, including fragmentation, urbanization, livestock grazing, recreational impact, water flow regulation and lowered water tables (Table A in S1 Table).

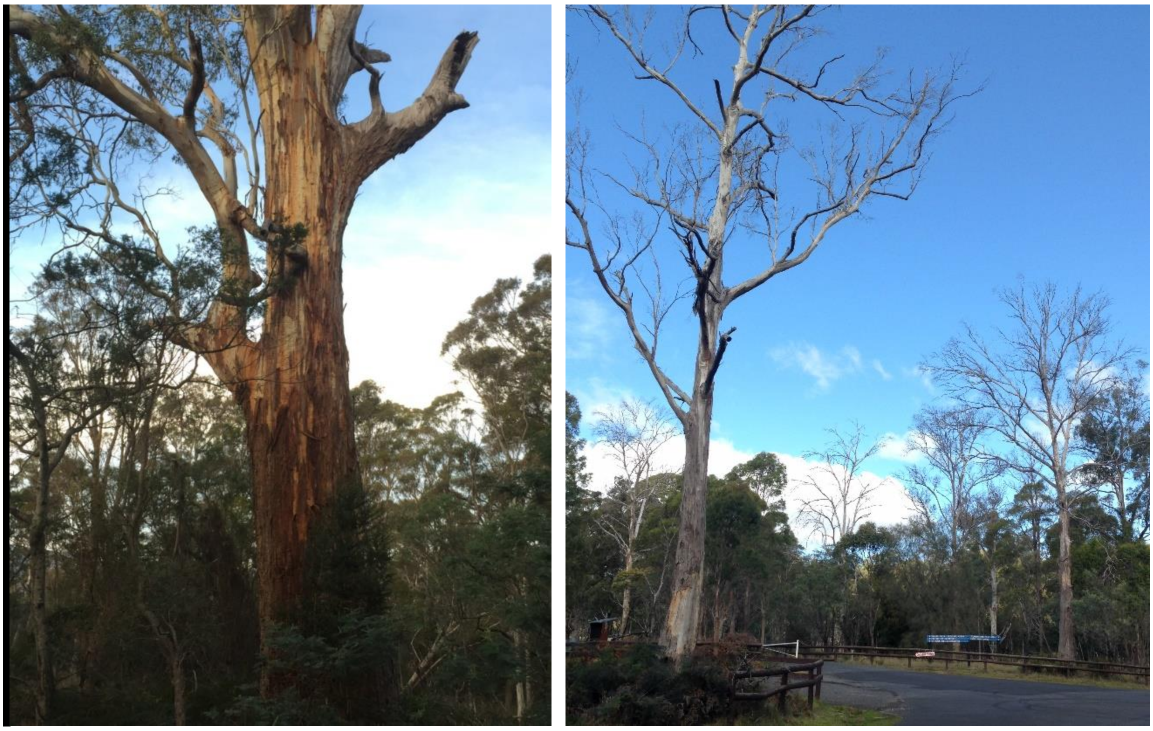

Plate 2 White gum (*Eucalyptus viminalis*) gingering and dying at accelerating rates in northern Tasmania (173a). Image credits: Anna Povey.

The most common consequence reported as resulting from plant deaths was habitat loss for animals. For example, observed death of 90% of the woodland mid-storey species *Melaleuca decussata* (Cross-leaf honey myrtle) was purported to have resulted in declines in small nesting birds such as blue wrens and thornbills, and less food for honeyeaters (243b, Junortoun, central Victoria). Mortality of *Banksia robur* (Swamp banksia) was perceived as a driver of the disappearance of small possums and birds in Wallum heath (326a, Cooloola Cove, Wide Bay-Burnett area, Queensland), and canopy thinning due to tree mortality was the perceived cause of the disappearance of small birds such as *Malurus lamberti* and *M. melanocephalus* (Variegated and Red-backed fairy-wrens), *Acanthiza pusilla* (Thornbill), *Sericornis* sp. (Scrubwrens) and robins (Family Petroicidae) (151f, Gunderlong, Clear Mountain, south-east Queensland). One anecdote described a cascade of hydrological and biotic consequences resulting from the unprecedented drought- and heatwave-driven fires killing the keystone tree *Eucalyptus delegatensis* (Alpine ash), including loss of litter and associated lizards, loss of tree hollows, and warming of alpine streams leading to decline in trout (Family Salmonidae) (see also 3.2.9 'Landscape processes', 186a, North East Catchment Management Region, Victoria).

Animal mortality. Anecdotes of animals dying attributed to climate change-related drivers involved all the major animal groups, including invertebrates, fish, amphibians, reptiles, birds and mammals, with drivers of mortality including drying out of lakes and wetlands, salinization, extreme heatwaves, firestorms, and changed conditions leading to food shortages or increased disease (Table B in S1 Table). For example, warming and increased drying of lakes and wetlands were reported as driving increases in death of fish (Hardyheads, Family Atherinidae) in warm, deoxygenated water of Lake George (75c, Beachport, south-east South Australia); and up to 10,000 *Emydura macquarii* (Murray River turtle) died after Lake Numalla in western Queensland dried out twice within a 10 year period (193a). Extreme heatwave events were the perceived drivers of mass death of *Pteropus poliocephalus* and *P. alecto* (Grey-headed and Black flying fox) in the Hunter and North Coast areas,

New South Wales (88b), and two independent reports of mortality in *Stagonopleura guttata* (Firetail finch) (161f, Lake Burrendong area, central west New South Wales; 239d, Tharwa, Australian Capital Territory). Lack of moisture in eucalypt leaves was a suggested direct driver of death of *Phascolarctos cinereus* (Koala) (107d, Strathbogie Ranges/Merton, central Victoria, see also impacts on koala disease in 3.2.5), and salinization of ponds and groundwater associated with sea-level rise was reported as driving mass death of tadpoles (e.g. *Limnodynastes dumerilii,* Banjo frog) in the Derwent River estuary in Tasmania (180a).

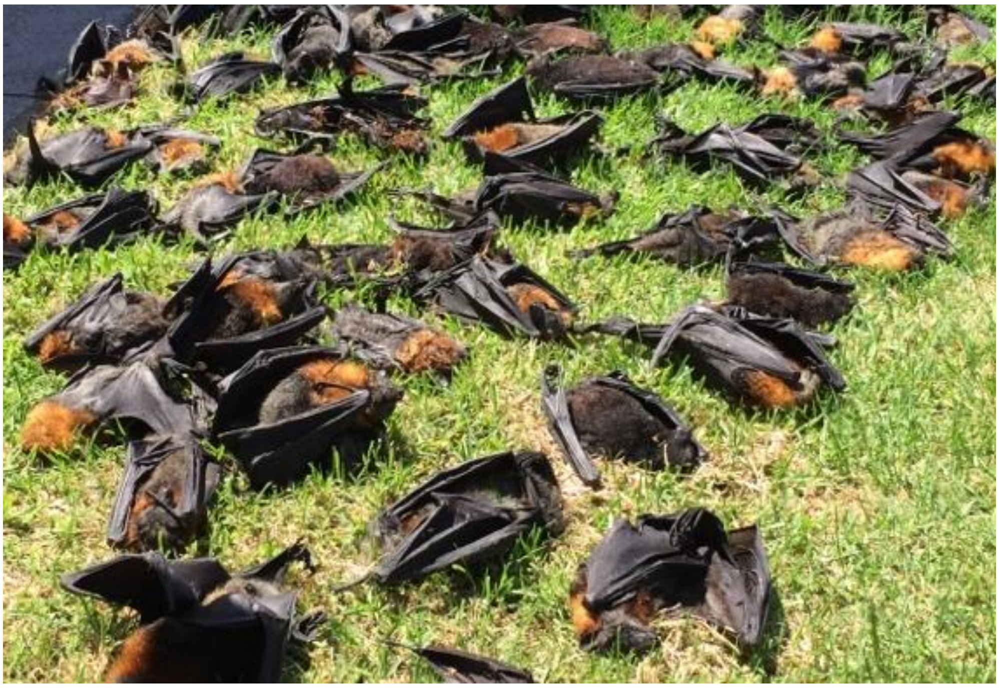

Plate 3 Mass mortality of *Pteropus poliocephalus* and *P. alecto* (Flying foxes) due to heat stress in the Singleton area, New South Wales, February 2017 (88a, b). Image credit: Von Naftel.

Mortality from lack of food was perceived to arise through a number of mechanisms. For example, *Litoria cyclorhyncha* (Spot-thighed frog) mortality in Ewlyamartup, Great Southern Western Australia, was attributed to reduced supply of mosquitos and bugs due to lack of rain (113e), starvation of *Rusa unicolor* (Sambar deer) was reported due to lack of sufficient food (forest understorey was severely browsed) in combination with extremely cold nights (85a, Yarra Ranges National Park, Central Highlands, Victoria), and starvation of young owls (Order Strigiformes) in Mullumbimby, north coast New South Wales (316b) and of juvenile *Pteropus poliocephalus* and *P. alecto* (Flying foxes) in the Hunter and North Coast areas, New South Wales (88a) were independently reported as resulting from changed flowering times. In the case of *Pteropus poliocephalus* and *P. alecto*, flowers and fruits were the direct food source, whereas for owls (Order Strigiformes), mortality was attributed to an ecological cascade where earlier flowering times led to earlier breeding of *Petaurus breviceps* (Sugar glider), with consequent mistimed supply of prey for young owls (316b, Mullumbimby, north coast New South Wales). *Trichosurus arnhemensis* (Northern brushtail possum) were reported as dying at an unprecedented rate from stress dermatitis in association with warmer conditions (97a, Darwin, Northern Territory).

Importantly, flow-on effects of these animal deaths were also occasionally described. For example, death of Hardyheads (Family Atherinidae) at Lake George (Beachport, south-east South Australia) was reported to lead to a steady decline in migratory wading birds (75c). From a social perspective, starvation of *Pteropus poliocephalus* and *P. alecto* (Flying foxes) was proposed as potentially driving

their increased colonization of urban areas (88b, Hunter and North Coast areas, New South Wales), but there were otherwise few reported interactions with land use drivers (Table B in S1 Table).

1.3 Organisms: physiology: pests and diseases

Anecdotal examples of pests and diseases in plants thought to be facilitated by climate change pertained to viruses (in *Banksia integrifolia*, Coast banksia), fungal parasites (*Phellinus sublamaensis,* White rot; *Phytophthora cinnamomic,* Phytophthora root rot), *Quambalaria coyrecup* (canker, in *Corymbia calophylla,* Marri), and various invertebrate outbreaks, including psyllids (Family Psyllidae), weevils (Superfamily Curculionoidea), scale (Superfamily Coccoidea), aphids (Superfamily Aphidoidea), borers (Class Insecta) and termites (Infraorder Isoptera). In most cases these were considered to result from climate- (sometimes in conjunction with non-climate-) induced stressors on the host plants, and as described in 3.2.3, and often resulted in excessive plant mortality. In some examples, changes in climate were purported to directly influence invertebrate populations. In particular, warming and more humid conditions were thought to facilitate overwintering of green and grey aphids (Superfamily Aphidoidea) in Mongarlowe (Southern Tablelands, New South Wales), and hence increases in aphid numbers (296a). Changes in bird populations were also thought to contribute to increases in pest insects. Examples included increases in psyllids (Family Psyllidae), scale and aphids (Superfamily Aphidoidea) associated with decline of smaller birds (due to arrival of larger, more aggressive birds such as *Anthochaera* sp. (Wattle bird) and *Manorina melanocephala* (Noisy miner) 296a, Mongarlowe, Southern Tablelands, New South Wales), psyllids (Family Psyllidae) outbreaks associated with arrival of bell miners (in turn attributed to *Lantana camara,* Lantana, invasion resulting from reduced frosts, 300b, Bulga, Hunter Valley area, New South Wales), and increases in pest species associated with decline in small predatory birds driven by drought-driven habitat loss (164f, Stoney Creek Nature Conservation Reserve/Kara Kara National Park, Redbank, south-west Victoria).

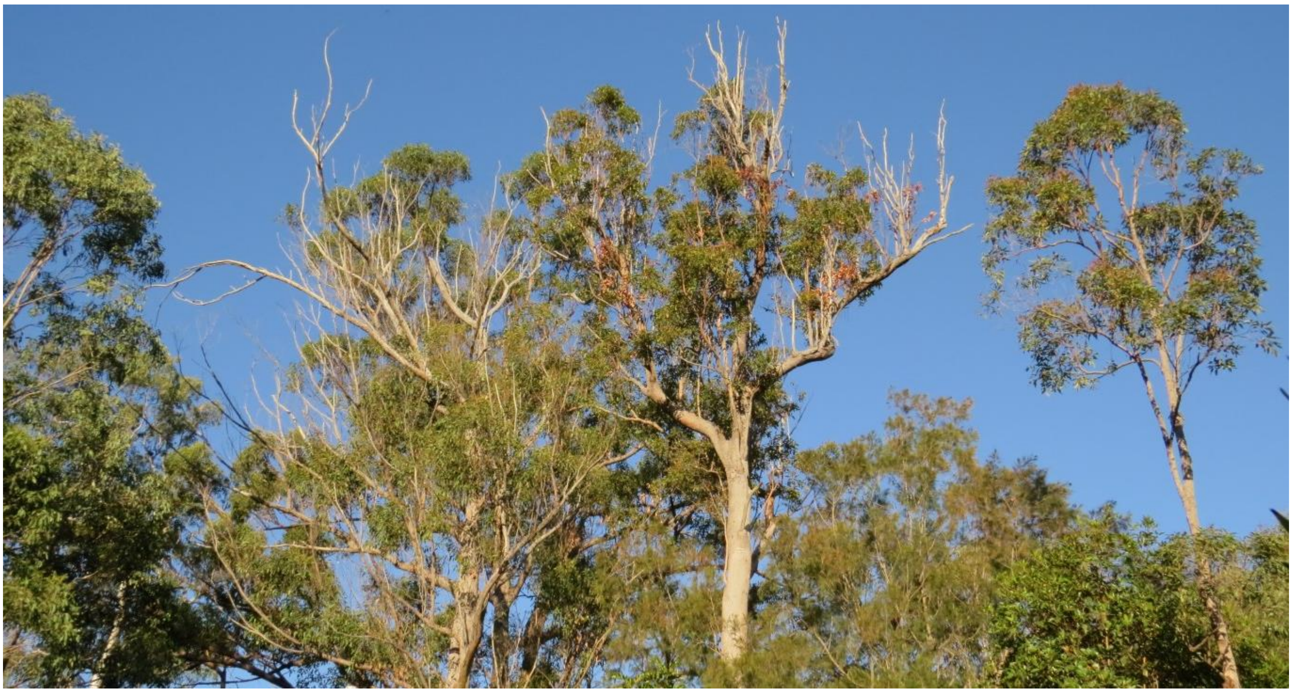

Plate 4 Increased tree stress and greater attack by psyllids and borers observed in eucalypts (119a) in south-east Queensland (reproduced with permission).

Although no clear driver was indicated, another severe case of insect outbreak noted in mallee (*Eucalyptus* spp.) in the Riverland Biosphere Reserve, Murray-Darling Basin area, South Australia, is worthy of further investigation: *'Each year since 2012, between Nov-April large areas (1000s of hectares) of mallee in this area is affected by a particular insect that strips the leaves off the mallee trees. Some years the outbreak is worse than others. It is not sustainable in the long run for these mallee trees to be virtually leafless during the summer months for many years in a row. Some have now died and I fear more will die if this outbreak keeps returning'* (201c, Peter Waanders).

Increases in pests and diseases affecting native animals were also reported as likely or potentially climate change-related. These increases were thought to arise directly through increasing climate suitability for the pest or disease (e.g. increase in *Haematobia irritans exigua*, Buffalo fly, affecting cattle in Caparra, north coast New South Wales due to warmer conditions, 266c; *Ixodes holocyclus*, Paralysis tick, arriving in Mongarlowe in the Southern Tablelands, New South Wales, with warmer, more humid summers, 296c; and increase in *Batrachochytrium dendrobatidis*, Chytrid fungus, in frogs (Order Anura), associated with warmer winters in Winter Swamp, Cardigan, in the Central Highlands of Victoria, 66a), or were attributed to greater climate-driven nutritional or moisture stress in the affected mammals. The latter include nutritional and moisture stress in *Phascolarctos cinereus* (Koala) contributing to *Chlamydophila pecorum* (Chlamydia) and bladder tumour (158a, Cashmere, south-east Queensland; 192a, Oakey, Darling Downs/south-west Queensland), and nutritional stress in *Pteropus poliocephalus, P. alecto, P. scapulatu* (Flying foxes) due to *'seasonal changes affect[ing] flying fox feed'*, in turn promoting *Henipavirus* sp. (Hendra virus) and spillover into *Equus ferus* (horses, 87a, Logan, south-east Queensland).

Outcomes of increases in pests and diseases are further discussed under 3.2.10 (Interspecific Relationships).

2 Populations

2.1 Populations: phenology

Life history processes of many species, including migration, breeding, leaf emergence and flowering, are closely linked with seasonal and inter-annual patterns in temperature and precipitation. Changes in such phenologies are some of the most commonly reported impacts of climate change worldwide, with evidence derived particularly from agricultural records and documentation of biotic events indicating the arrival of spring in the northern hemisphere **(Parmesan 2006)**. While there are fewer data recorded from Australia, phenological changes were widely reported in our survey.

Plant phenology. Anecdotal information referred to a range of plant phenological changes, including aberrant or early timing of flowering, failed or weakened flowering or fruiting, altered growing season lengths, and other altered seasonal responses. Drivers were most commonly warmer temperatures - expressed variously as shorter winters, hotter days, earlier arrival of spring, warmer wet periods, earlier hot days, less clear triggers for stratification (unpredictable frosts), hotter heatwaves - and changes in rainfall patterns, particularly lack of spring rains and later autumn breaks.

Earlier flowering was reported most commonly for garden or orchard plants, for example, perennial crops such as *Prunus dulcis* (Almond) and *Vitis vinifera* (Grape) flowering earlier (47c, Naracoorte Caves National Park, south-east South Australia), *Prunus* sp. (Stone fruit) starting 'spring' flowering in July (155b, Amamoor, Wide Bay-Burnett area, Queensland), and a *Pyrus* sp. (Pear) flowering in autumn: *'There's an old pear tree in the village that has been flowering in autumn for the past several years...I think it might be less clear triggers for stratification - unpredictable frosts, and earlier warming'* (296c, Mongarlowe, Southern Tablelands, New South Wales, Anon.). Earlier flowering was also reported for *Eucalyptus macrocarpa* and *E. polyanthemos* (eucalypts) (53a, Cumberland Plain, Sydney Basin, New South Wales; 206c, Kangiara, south-east New South Wales), *Stenocarpus sinuatus* (Queensland firewheel tree) (316b, Mullumbimby, north coast New South Wales), terrestrial orchids (279a, Rocky Cape National Park, north-west Tasmania), *Banksia menziesii* (Firewood banksia, by 2-4 weeks) (246a, Neerabup, Perth area, Western Australia), and in four independent instances for *Acacia* species. For example, one respondent noted *'*Acacia genistifolia *could be relied on to flower in July. It now flowers earlier and this year flowered in May'* (7d, Bungendore, south-east New South Wales, Anon.) and another noted *'Wattle Day on September 1 is almost too late this year as most wattles are well into flower now'* (August 2017, 292a, Mt Lofty Ranges, South Australia, Anon.).

While most changes to flowering time involved earlier flowering, it sometimes involved other shifts. For example, *Corymbia calophylla* (Marri) in the Wheatbelt, Western Australia, was observed flowering in autumn/winter (compared with records indicating December-May, 319c, Florabase), gums (*Eucalyptus* spp.) were reported to flower *'at odd times of year'* (47c, Naracoorte Caves National Park, south-east South Australia), and flowering time in *Acacia* spp. (Wattles) and native peas (Family Leguminosae) in Eppalock, north central Victoria, was described as *'confused'* (170d). Observations in exotic species included unpredictable and longer flowering in *Baccharis halimifolia* (Groundsel bush, 135b, Bald Hills, south-east Queensland), and flowering up to 3 months late in *Pyrostegia venusta* (Orange trumpetvine, *'perhaps needs cooler temperature due to warmer summer'*, 131a, Gillen, Alice Springs, Northern Territory, Margaret Friedel). Sometimes, the degree or amount of flowering was affected rather than the timing, for example buds but not flowers were observed to develop in *Eucalyptus tereticornis* and *E. tindaliae* (Forest red gum and Tindale's

stringybark, 119b, Tamborine Mountain, south-east Queensland), summer flowering was perceived to be weaker in *Eucalyptus moluccana* (Grey Box) on the Cumberland Plain Sydney Basin, New South Wales, (53a), and repeated flowering was observed in trees in Ewlyamartup, Great Southern Western Australia: *'eucalypts have flowered twice plum trees flowered twice'* (113d, Anon.).

Changes in fruiting time were less commonly detailed. In one case from Upper Coopers Creek, north coast New South Wales, earlier seeding was proposed to increase exotic invasions: *'the small leafed privet* [Ligustrum sinense] *is producing viable seed up to 3 months earlier than 12 years ago. This gives this exotic weed an added advantage in preventing the germination of native seedlings'* (153a, Maggie Wheeler). The remaining examples involved later seeding in grasses due to lack of spring rains (192c, Oakey, Darling Downs/south-west Queensland), unseasonal fruiting of *Archontophoenix cunninghamiana* (Bangalow palms, 316b, Mullumbimby, north coast New South Wales), and *Prunus* sp. (Plum trees) setting fruit in winter (June) in Ewlyamartup, Great Southern Western Australia (113d). A suite of anecdotes reported failed or reduced fruiting (and sometimes mortality) due to shortened growing seasons. These included annuals dying due to lack of rain before they could set seed (313b, Stratton, Perth area, Western Australia), and failed fruiting in sedges, orchids and other herbs due to inadequate season length before drought sets in (119b, Tamborine Mountain, south-east Queensland; 49a, Harcourt, Central Highlands Victoria; 32a, Plenty Gorge Park, Bundoora, Melbourne area, Victoria). One respondent described the consequences of shortening at both ends of the growing season as follows:

'Caladenia amoena *(Charming Spider-orchid) is re-emerging later in the year, on average by two weeks, and the length of time above ground has reduced with the season also finishing between two to four weeks early consistently. Other plants also showing this pattern, late emerging plant[s] rarely re-emerge the following year (ie they die). The shortening seasons appear to be impacting on the ability of the plants to replace their tubers, has resulted in reduced flowering, reduced ability to produce seed and a greatly reduced ability to recruit seedlings into the population'* (32a, Plenty Gorge Park, Bundoora, Melbourne area, Victoria, Garry French).

Other flow on consequences of changes to flowering or fruiting to animals included food shortages causing starvation in *Pteropus poliocephalus* and *P. alecto* (Flying Foxes) and young owls (Order Strigeformes), as described under 3.2.4 'Animal mortality'.

Decreases or increases in growing season length were also occasionally reported as driving decreases or increases in ecosystem productivity. For example, reduced growth of grasses due to shorter cool-season growth periods was reported for the Lake Burrendong area, central west New South Wales (161c), and a notable increase in pasture productivity was reported in the Kangaroo Valley (south coast New South Wales):

'In our area, when we arrived in 1988, we were advised by our neighbour, local farmer (farming since mid 1950s) that kikuyu grass dies back in May and does not commence regrowing until November. This was our experience. We had cattle on our property from this time, and needed to feed them throughout the winter. ....Kikuyu now grows all year and throughout the winter. We hardly supplement feed, mostly to get them to come into the yards. We have not had a bad frost for years. I cannot remember when the last one was. Kangaroo Valley is now predominately green throughout the winter.' (303a, Greg Thompson, Brogers Creek Landcare).

Consequences of longer plant growing seasons were described by a respondent writing about the raptors of Gardners Bay, southern Tasmania as:

'The local raptors: sea eagles, wedge tailed eagles, peregrine falcons and goshawks of both colours are more abundant. We attribute the increased numbers of raptors to the abundance of small birds, mammals, and reptiles in the area. Small marsupials are particularly prevalent. This, in turn, is most likely due to the flourishing of plant species. Warmer temperatures and increased levels of atmospheric CO_2 mean longer growing seasons and more abundant growth.' (240a, Anon.).

Other disruptions to normal growth patterns included deciduous trees retaining their leaves *'right into winter'* (319c, Bakers Hill, Wheatbelt, Western Australia, Anon.), unseasonally early flushes of plant growth triggered by early spring warmth, leading later to die-off of the new growth due to frost (288a, Barrington Tops, Hunter Valley, New South Wales), and in Amamoor, Queensland, being *'able to grow summer crops in winter...(e.g. harvesting marketable sweet corn in July, winter too warm for peas'*, 155b, Steve Burgess).

Animal phenology. Of the >30 text anecdotes pertaining to animal phenology, all but two were perceived to be potentially related to climate change. Most of these applied to birds or invertebrates of native or unspecified origin, with only three cases involving exotic animals (*Gallus gallus*, domestic chicken; *Apis mellifera*, European honey bee; *Danaus plexippus*, Monarch butterfly; Table E in S1 Table).

The anecdotes referred to three main types of phenological change in animals: earlier arrival of migratory species, longer or shorter seasons of presence, and (typically) earlier observation of activities such as breeding, hatching or swarming. Animals observed arriving earlier included 'pollinators and other insects', sea birds, and terrestrial migratory birds such as *Eudynamys orientalis* (Eastern koel), *Dicrurus bracteatus* (Spangled drongo), *Sphecotheres vieilloti* (Figbird), *Chrysococcyx basalis* (Horsefield's bronze cuckoo) and *Eopsaltria australis* (Eastern yellow robin) (Table E in S1 Table).

Species arriving less reliably included altitudinal migrant birds in Stoney Creek Nature Conservation Reserve/Kara Kara National Park, Redbank, south-west Victoria (164d), and *Strepera graculina* (Pied currawong) in Berremangra, Riverina, New South Wales (*'normal winter arrival of currawongs is becoming rare'* (265a, Anon.). Conversely, *Strepera graculina* in Mullumbimby, north coast New South Wales, were noted as remaining present for longer than normal: *'Unusual and unseasonal fruiting of Bangalow Palms keeps birds like Currawongs in the area instead of them migrating as they used to do ten years ago'* (316b, Anon.). Other birds showing extended periods of presence included the *Acanthagenys rufogularis* (Spiny cheeked honeyeater, *'Warming conditions have made the season favourable for the feeding of the Spiny Cheeked Honeyeater longer, and these birds are more likely to over-winter at Lake George than previously'*, 75a, Anon.), and *Scythrops novaehollandiae* (Channel-billed cuckoo, now wintering in Mullumbimby, north coast New South Wales, rather than migrating to New Guinea, 316d). Flying foxes (Family Pteropodidae, 239f, Tharwa, Canberra, Australian Capital Territory) and invertebrates such as green and grey aphids (Family Aphidoidae, 296a, Mongarlowe, Southern Tablelands, New South Wales) and *Danaus plexippus* (Monarch butterfly, 300c, Bulga, Hunter Valley area, New South Wales) were similarly observed to be overwintering or more active in winter.

Earlier breeding activity was a frequent observation consistently associated with warming environments, with six cases of earlier nesting in birds (sometimes leading to two broods in one season e.g. for *Gallus gallus*, domestic chicken, 240c, Gardners Bay, southern Tasmania), and one indicating earlier breeding in mammals (*Petaurus breviceps*, Sugar glider, 316b, Mullumbimby, north coast New South Wales). Mosquitoes and midges on the other hand, were noted as 'breeding longer into winter' (151c, Gunderlong, Clear Mountain, south-east Queensland). Other phenological traits

observed to occur earlier were the emergence of moths in Bulga, Hunter Valley area, New South Wales (300a), appearance of *Heteronympha merope* (Common brown butterfly) in the Strathbogie Ranges/Merton, central Victoria (107c), hatching of (unspecified) reptiles in Curramore, south-east Queensland (152c) and swarming of *Apis mellifera* (European honey bee), for example: *'Bees used always to swarm in the first week of November, now it can occur in late September or October. I remember because they used always to swarm on my sister's birthday'* (157e, Westmere, south-west Victoria, M. T. Casanova).

2.2 Populations: age structure, recruitment and abundance

Climate stressors can influence plant and animal population dynamics directly or via effects on mortality, disease or phenology as described above, to result in observable effects on recruitment, population age structure, abundances and sex ratios (Scheffers et al. 2016). Anecdotes revealed numerous examples of increasing and decreasing levels of recruitment and abundance. Although only one anecdote referred explicitly to population age structure (mass mortality in *Emydura macquarii*, Murray River turtle, at Lake Numulla, south-west Queensland, led to significant change in the age demographics, 193a), and none referred to sex ratios; examples regarding recruitment and abundance may inherently involve such changes.

Plant recruitment and abundance. Anecdotal information included numerous cases of unusual levels of plant establishment or changes in plant abundance that respondents attributed as potentially due to climate change (Table F in S1 Table). These anecdotes were commonly associated with other changes, including plants dying or new species arriving in the area. Notably, anecdotes describing declining plant establishment pertained solely to native species, particularly trees such as *Eucalyptus delegatensis* (Alpine ash), *Eucalyptus populneus* (Poplar box), *Eucalyptus camaldulensis* (assumed, Red gum), mangroves, *Acacia carneorum* (Purple-wood wattle), *Banksia marginata* (Silver banksia), *Callitris collumellaris* (Murray pine) and *Allocasuarina luehmannii* (Buloke). The native grasses *Imperata cylindrica* (Blady grass) and *Triodia* sp. (Spinifex) and unnamed shrub species were also observed to be regenerating poorly. Drier and/or hotter conditions, sometimes combined with land use drivers or exotic invasions, were the most common stated causes of declining plant recruitment. Mistletoe (and with it *Dicaeum hirundinaceum*, the Mistletoebird) was also seen to be declining in association with the millennium drought; as for a range of cases nominating the millennium drought, it is uncertain whether this was exacerbated by climate change, 188d, Bendigo, north central Victoria).

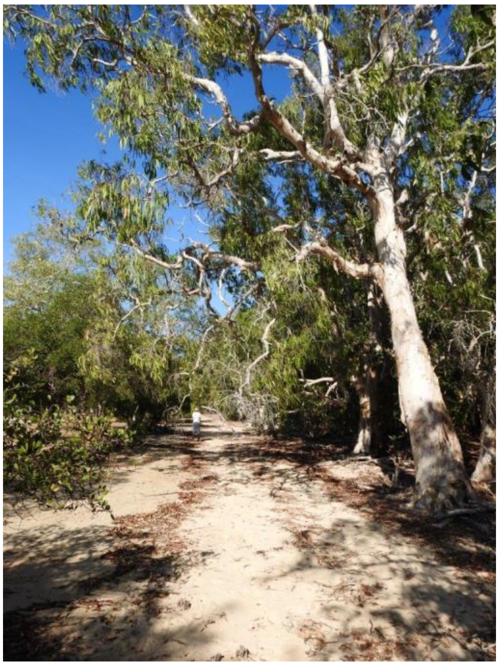

Image 5. ***Melaleuca leucadendra*** **(Broad-leaved paperbark) has been observed spreading inland, at Sandfly Beach, Northern Territory, as mature trees are affected by rising sea levels. Image with permission: Kim McLachlan.**

Cases of increasing plant recruitment or abundance included both native and exotic species. Notable examples involved thickening or localized movement of local native species, including *'Thickening of native acacias on largely open Mitchell Grass Downs country'* (13a, Augathella, south-west Queensland, Anon., associated with climate-induced reduction in hot fires combined with reduced sheep grazing), *Rhizophora stylosa* (Red mangrove) establishing in high density on a part of Sandfly Beach in the Northern Territory that was once sandy (198b), regeneration of *Melaleuca leucadendra* (Broad-leaved paperbark) 20-30 m behind the line of tree mortality associated with rising salinity levels at the same beach (198a), *Triodia* spp. (Spinifex) expanding into more marginal habitats of the far north Queensland area in unusually wet years (25a), and for a prior freshwater lake of Joondalup, Western Australia, trees *'taking hold where the wetlands shrunk and the lake dried up'* (246b, Anon.). Other observations of increases in establishment of native species involved species apparently from further afield, such as *Rhagodia candolleana* (Red seaberry *,* a coastal species) establishing *'at a remarkable rate in the last 20 years'* in regenerating bushland near Beachport, south-east South Australia, as conditions became hotter and drier (75b, Anon.), non-local *Acacia longiflora* (Sallow wattle) 'overtaking' in the Wimmera Region, south-west Victoria, after 20 years of extreme conditions (drought and hot fires, 73a), and plants from drier regions establishing in south-east Queensland after mortality of the local *Casuarina* (e.g. *Jagera pseudorhus,* Foam Bark; *Acacia fimbriata,* Brisbane Wattle; *Ficus* sp., Figs; and *Sterculia quadrifida,* Peanut trees, 132c).

Increased establishment or abundance of exotic species involved mostly herbaceous species, including increasing exotic annuals in south-eastern Australia (e.g. *'annuals such as chickweed and* Briza *resulting from increased warming and less effective rainfall,* 170a, north central Victoria, Anon.; thistles and *Echium plantagineum* (Paterson's Curse) during wet periods after drought or fire; 14c, Tasmania, 207a, Callum Brae Nature Reserve, Symonston, Australian Capital Territory), increased establishment of perennial grasses such as *Cenchrus pennisetiformis* and *C. ciliaris* (Buffel grass species) into drier habitats of north-west Queensland and central Australia due to *'more large rainfall events'* (90a, Simpsons Gap National Park, Alice Springs, Northern Territory, Ashley Sparrow) or *'variation in the northern monsoon'* (25a, north-west Queensland, Dan Kelman, Qld Herbarium),

and increased invasion of heathland, woodland and roadsides by African bulbous weeds *(Gladiolus caryophyllaceus,* Wild gladiolis; *Sparaxis bulbifera*, Harlequin flower) purportedly due to warming and drying in association with proliferation of recreational tracks (252a, Balmoral, south-west Victoria). Woody exotic species reported to be increasing included subtropical fan palms germinating in the Swan Coastal Plain, Western Australia, wetlands due to warmer conditions (86b), and rampant invasion by *Rubus fruticosus* (Blackberry) in conjunction with forest logging and warming in the Central Highlands and alpine areas of Victoria (223b).

Cascading ecological consequences of these changes in plant establishment or abundance were occasionally mentioned. Examples included a perceived decline in *Macropus rufogriseus* (Red-necked wallaby) and *Wallabia biocolor* (Swamp wallaby) due to sparser understoreys associated with poor establishment of *Imperata cylindrica* (Blady grass) and other native grasses (151e, Gunderlong, Clear Mountain, south-east Queensland), and disease and decline in *Phascolarctos cinereus* (Koala) associated with limited availability of young, nutritious eucalypt leaves, in turn attributed to prolonged droughts and heatwaves limiting seed germination and survival of *Eucalyptus populnea* (Poplar box) in the Oakey area of south-east Queensland (192a).

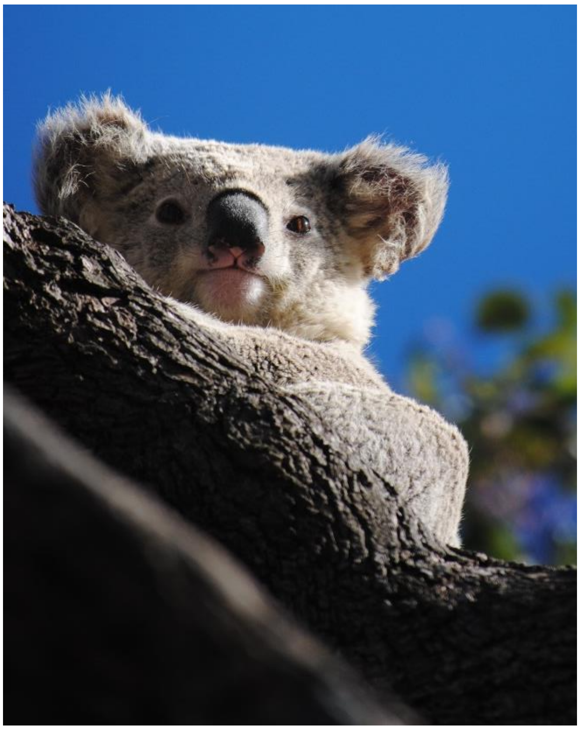

Image 6 *Phascolarctos cinereus* (Koala) in a *Eucalyptus populnea* (Poplar box) tree in the Oakey district, where *Phascolarctos cinereus* were reported to have declined substantially, partly attributed to prolonged droughts and heatwaves limiting *Eucalyptus populnea* seed germination, recruitment and leaf quality (192a). Image credit: N. Laws.

Animal abundance. Anecdotes describing changes in animal abundance were often directly attributed to warming and/or drying, including numerous cases of decline in aquatic species due to drying out of wetlands or dams (insects, fish, frogs, lizards), and more direct negative impacts of drying and warming on moths (Order Lepidoptera), mosquitoes (Family Cuculidae), leeches (Subclass Hirudinea), snakes (Suborder Serpentes), *Tiliqua rugosa* (Bobtail/Shingleback)*, Pogona minor* (Western bearded dragon), at least 13 bird species, and mammals such as possums (Family Phalangeridae), *Tachyglossus aculeatus* (Short-beaked echidna) and *Antechinus* spp. (Antechinus) (Table G in S1 Table). For example, decline in moths (Order Lepidoptera) in the Bulga, Hunter Valley area, New South Wales, region was attributed to early emergence on unseasonably warm winter

days, followed by mortality with return of cooler weather (300a). Another direct climate-change driver involved increased salinity in coastal wetlands leading to decline in birds (180b, Derwent River Estuary, southern Tasmania; Table J in S1 Table).

Decreases in animal abundance were also indirectly attributed to climate change through ecological cascades, via impacts on vegetation, food supplies or other processes. For example, decline in Thynine wasps (Family Thynnidae) in Box Ironbark forests of Victoria was attributed to declines in the local *Hakea* and ground-layer plants (32b, Plenty Gorge Park, Bundoora, Melbourne, Victoria), decline in trout (Family Salmonidae) in the North East Catchment Management Region, Victoria, was attributed to warming of mountain streams resulting from extreme drought- and fire-driven loss of *Eucalyptus delegatensis* (Alpine ash) forests (see above, 186a), decline in *Artamus cyanopterus* (Dusky woodswallow) was attributed to competition from other *Artamus* (Woodswallow) species seeking refuge from persistent warming (161e, Lake Burrendong area, central west New South Wales) and decline in butterflies (Order Lepidoptera) was attributed to disconnects between the timing of food plants' flowering and butterfly emergence (316b, Mullumbimby, north coast New South Wales). While most reported decreases in animal abundance involved native species, declines in *Sturnus vulgaris* (European starling) and *Vulpes vulpes* (European red fox) were also reported (28b, Cape Paterson, Gippsland, Victoria; 87b, Logan, south-east Queensland). Perceived *Vulpes vulpes* decline in Logan, for example, was also attributed to an ecological cascade: *'The warmer weather has led to an increased season for paralysis tick* [Ixodes holocyclus] *and this has resulted in a marked decline in fox numbers'* (87b, Anon.).

Anecdotal reports of increases in animal abundance were notably less common than decreases in abundance (Figure 4 main text). They typically involved increases in common, larger birds and mammals such as parrots (*Licmetis* sp., Corella; *Eolophus roseicapilla*, Galah; *Cacatua galerita*, Sulphur-crested cockatoo), *Ocyphaps lophotes* (Crested pigeon); *Nycticorax caledonicus* (Nankeen night heron), *Rusa unicolor* (Sambar deer), *Macropus giganteus* (Eastern grey kangaroo) and Wallabia bicolor (Swamp wallaby), typically due to warming and/or drying. A notable exception was a purported increase in small birds, small marsupials and reptiles, and subsequent increase in raptors, attributed to improved plant productivity (see 3.2.6, 240a, Gardners Bay, southern Tasmania; Table J in S1 Table). Other increases in animal abundance involved a suite of invertebrates, including beetles (Order Coleoptera), locusts (Family Acrididae), psyllids (Family Psyllidae) and scale (Superfamily Coccoidea) purported to be increasing due to warming or drying, increased numbers of termite (Infraorder Isoptera) nests due to increased drought and heat stress in eucalypts (see also 3.2.5), and swarming of insects due to larger rainfall events and localized flooding.

While cascading effects were common drivers of changes in fauna abundance, changes in fauna abundance were also occasionally reported as drivers of further ecological change. This includes increases in raptors due to increases in small prey animals as mentioned earlier (240a, Gardners Bay, southern Tasmania; Table G in S1 Table), and disappearance of *Westralunio carteri* (Freshwater mussel), due to decline in fish they rely on for dispersal and reproduction (86a, Swan Coastal Plain, Western Australia). Another such cascade involved thynine wasps (Family Thynnidae) as an intermediary to effects on plant pollination: *'Key species required for the associated thynine wasp pollinator (local* Hakea *and the grassy field layer) are also being impacted upon through the drier conditions, thus potentially reducing the ability of the orchid* [Caladenia amoena *(Charming Spider-orchid)*] *to be naturally pollinated.'* (32b, Plenty Gorge Park, Melbourne, Victoria, Garry French).

Although respondents commonly attributed changes in animal abundance to both land use and climate-change drivers in Part 1, explicit mention of interactions among drivers in anecdotal text was only occasional. These included potential exacerbation of reptile decline in the Perth region by poachers (313a, Stratton, Western Australia), exacerbated decline in small birds and wallabies in Gunderlong, Clear Mountain, south-east Queensland, due to increased traffic and human habitation (151e), and interactions between climate drivers, fire suppression and cat predation contributing to the decline of the *Pachycephala rufogularis* (Red-lored whistler) in the Riverland Biosphere Reserve, South Australia (201b). Reported interactions with climate change resulting in increases in animal abundances include increased grain spillage benefiting large parrots (*Eolophus roseicapilla*, Galah; *Cacatua galerita*, Sulphur-crested cockatoo; 7c, Bungendore, south-east New South Wales), potential interactions between removal of cattle grazing and increasing *Rusa unicolor* (Sambar deer) in treeless alpine vegetation (82a, Central Highlands, Victoria), and logging and clearing contributing to increases in *Macropus giganteus* (Eastern grey kangaroo) and *Wallabia bicolor* (Swamp wallaby, 220a, Moruya, south coast New South Wales).

3 Species

3.1 Species: distribution

Another common and rapid response of species to climate change is to track changing environments by shifting their distributions. This is especially apparent for mobile species, with many examples pertaining to birds, freshwater fish and marine taxa, whereas there can be greater time lags between climate change and plant responses (Scheffers et al. 2016). From the perspective of observing change in specific area or location, shifts in species distributions (including total extinction) are evident through disappearance of species previously known to be present, and appearance of new species.

Species disappearing. More than forty different species, encompassing invertebrates, amphibians, reptiles, birds, mammals, plants and fungi (to different levels of taxonomic resolution) were reported anecdotally as having disappeared in recent years potentially in association with climate change-related drivers (Table H in S1 Table). Similar to observations of decline in native but not exotic plants, these reports pertained solely to native species.

Warming and/or drying were implicated in almost all text anecdotes of species disappearing, with exceptions including unspecified changes in climate leading to extinction of the *Taudactylus dirurnus* (Day frog) via the *Batrachochytrium dendrobatidis* (Chrytrid fungus) and possible interactions with pesticides (56a, Mt Glorious, south-east Queensland), failure of *Swainsona procumbens* (Broughton's pea) to re-appear in pasture in Echuca, north central Victoria, after unprecedented wet conditions (77b), and failure of breeding in *Amytornis striatus* (Striated grasswren, 201b) of the Riverland Biosphere Reserve, South Australia, after unspecified extreme climate events. Drying of wetlands or waterbodies was frequently a purported driver, perceived to be causing disappearance of *Cherax quinquecarinatus* (Giligie, 139c, Jane Brook, Perth area, Western Australia), *Westralunio carteri* (Freshwater mussel , 86a, Swan Coastal Plain, Western Australia; 139c, Jane Brook, Perth area, Western Australia), four frog species (151a, Gunderlong, Clear Mountain, south-east Queensland), turtles (Order Testudines) and *Varanus* sp. (Monitor) (the latter two interacting with nutrient enrichment via algal blooms, 129a, Goegorup Lake, Barragup, Peel area, Western Australia), *Gallinula tenebrosa* (Dusky moorhens, 132a, Boondall Wetlands Conservation Reserve, Boondall, south-east Queensland), *Hydromys chrysogaster* (Water rat, 178a, Flagstaff Memorial Nature Reserve, Cootamundra, New South Wales) and stream bank plant species (*Cyathea cooperi*, Scaly

tree fern, 145a, Carindale, south-east Queensland). Species reported to be disappearing due to drought or drying of terrestrial environments (sometimes in interaction with fragmentation or fuel reduction burning) included *Dromaius novaehollandiae* (Emu) and *Coturnix* sp. (Quail) from Coleambally, Riverina region of New South Wales (71a), small possums (Family Phalangeridae) from Wallum heath (326a, Cooloola Cove, Wide Bay-Burnett area, Queensland), *Isoodon* sp. (Short-nosed bandicoot) from the Mt Lofty Ranges, South Australia (292c), and *Ornithogalum umbellatum* (grass lilies) and *Orchidaceae* (orchids) from Carindale, south-east Queensland (145a), although sometimes it was unclear whether respondents considered dry conditions to be climate-change driven (e.g. the millennial drought). An example of the latter involved a cascade leading from plant to animal disappearance:

'Ground cover plants, including common heath [Epacris impressa]*, blue pincushions* [Brunonia australis]*, pink bells* [Tetratheca ciliata] *have disappeared, and bracken has become sparser. This has led to the local extinction of the rufous bristlebird and the long-nosed bandicoot and possibly the potoroo... It seems the only explanation for loss of plants is the long millennial drought (1996-2010).'* (159a, Naringal East, south-west Victoria, Shirley Duffield).

Disappearances and concurrent appearances of species associated with rainfall decline were also implied in an anecdote from south-western Australia *'Forest species have moved west, wheat belt species are moving in from the east. Rainfall has gone from 21 inches a year to 14 inches a year.'* (17d, Trevor Bunce).

Warming alone was attributed as a driver for apparent disappearance of the Western Australian endemic shrub *Pimelea spectabilis* (Bunjong, 191a), and of *Pogona* sp. (Bearded dragon) in Junortoun, central Victoria: *'Bearded dragons were always around previously but not sighted at all for a few years. Climate change by way of warming seems to be the major culprit.'* (243c, Anon.), but warming was more commonly considered to act in concert with drying. This included interactions with intense fire, such as disappearance of lizards due to loss of the litter layer after repeated unprecedented fires across Alpine Ash landscapes (186a, North East Catchment Management Region, Victoria), and catastrophic extinctions of *Thylogale stigmatica* (Red-legged pademelon) and *Notamacropus dorsalis* (Black-striped wallaby) in hot dry years after unprecedented wildfire in the Forty Mile Scrub National Park, south-east Queensland (166a). Warming and drying effects on animals were also commonly mediated by direct effects on plants, including disappearance of small birds such as *Malurus melanocephalus* (Red-backed fairywren) and *Malurus lamberti* (Variegated fairywren) at Gunderlong, Clear Mountain, south-east Queensland due to drought and heatwave-induced thinning of bushland habitat (151f), disappearance of *Dicaeum hirundinaceum* (Mistletoebird) from the same location and Bendigo, north central Victoria, when mistletoes disappeared (151g, 188d), and disappearance of gliders (Family Petauridae) from Strathbogie Ranges/Merton, central Victoria, due to lack of moisture in stressed eucalypts (on which they feed) (107d).

New species arriving. Anecdotal evidence describing new plant species arriving in association with potential climate-change drivers pertained almost exclusively to exotic species (13 species, Table I in S1 Table), consistent with the contention that climate change is expected to increase exotic invasions (Walther et al. 2009). Examples include *'warmer conditions'* enabling *'exotic fan palms to germinate and grow in wetlands and along stream channels'* (86b, Swan Coastal Plain, Western Australia, Associate Professor Belinda J Robson), and *'extreme weather patterns...causing...increase in flooding'* resulting in invasion by exotics such as *Onopordum* spp. (thistles) and *Proboscidea lutea* (Devil's claw) in floodplain woodlands of the Murrumbidgee River, New South Wales (275a, Anon.).

One native plant species (a *Ficus* sp., Fig) was also reported as a new arrival in the Boondall Wetlands Conservation Reserve, south-east Queensland (132c).

On the other hand, appearance of new animal species most commonly pertained to native species (16 native species, Table I in S1 Table), with only one exotic bird (*Acridotheres tristis*, Indian myna, 7c, Bungendore, south-east New South Wales) and two presumed exotic insects (a moth, 170a, Eppalock, north central Victoria; *Vespula germanica*, European wasp, 292d, Mt Lofty Ranges, South Australia) reported as arriving in association with climate change. Most of the native species arriving were birds (13 species) including the *Trichoglossus moluccanus* (Rainbow lorikeet) in Bullen Range Nature Reserve, Australian Capital Territory, and Bungendore, south-east New South Wales, attributed potentially to milder winters, urbanization, and/or early eucalypt flowering (5b, 7c), and *Lichmera indistincta* (Brown honeyeaters) and *Aprosmictus erythropterus* (Red-winged parrots) in Dubbo, central west, New South Wales: *'Brown Honeyeaters and Red-winged Parrots now regular migrants and residents (respectively). Not the case 20 years ago'* (163a, Tim Hosking). Other animals arriving included four invertebrate species – a Chickweed-dependent moth (Order Lepidoptera), *Vespula germanica* (European wasp), fireflies (Family Lampyridae) and *Ixodes holocyclus* (Paralysis tick), the *Litoria caerulea* (Green tree frog), and several mammal species (*Phascolarctos cinereus* (Koalas), Flying foxes (Family Pteropodidae), *Oryctolagus cuniculus* (European rabbit) (Table I in S1 Table). For example:

'According the NSW NPWS Atlas, no Green Tree Frogs [Litoria caerulea] *were recorded at Willandra National Park prior to 2009 (1974-2009). However, following heavy rains in 2010, green tree frogs were observed to colonise and successfully breed at the creeks and ephemeral water holes on the park. They appear to have established in the area in the within the last decade. This may be a result of changes in temperature and rainfall patterns associated with climate change'* (185a, Anon.).

About one third of new arrivals applied to species appearing at higher elevation due to warming. This included two of the invertebrate species, in particular, one respondent reporting on Lowanna, north coast New South Wales, noted: *'Over the past 20 years have watched the fireflies migrate up our mountain'* (272a, Anon.), and another noted that *Ixodes holocyclus* (Paralysis tick, predominantly a coastal lowland species) is *'now found in our area of the tablelands'* (296d, Mongarlowe, Southern Tablelands, New South Wales, Anon.). Other species observed arriving at higher elevation included *Artamus personatus* and *Artamus superciliosus* (Masked and White-browed woodswallows) *'seeking water and habitat in a cooler environment'* (161e, Lake Burrendong area, central west New South Wales, Long term observations by Neville Mattick/Hargraves NSW), and *Phascolarctos cinereus* (Koala) moving up the mountain away *'from the increasingly hotter and habitat-depleted lowlands below'* (95e, Tamborine Mountain, south-east Queensland, Anon.). Notably, *Menura* sp. (Lyre birds) were reported as moving in the opposite direction: *'arriving from higher on the mountain only in the last few years, moving to moist gullies'* due to drier, more erratic rainfall (33b, Grassy Head, north coast, New South Wales, Anon.). A suite of weed species observed appearing at higher elevation included *Ageratina adenophora* (Crofton weed), *Ageratina riparia* (Mist flower), *Lantana camara* (Lantana) (272a, Lowanna, north coast New South Wales), *Chrysanthemoides monilifera* (Boneseed), *Freesia* spp. (Freesia), *Watsonia* spp. (Watsonia) and *Chenopodium album* (Goose foot) (16a, Aldgate, South Australia).

Several anecdotes also referred to potential southward or coastward range shifts. These included *'dryland bird species arriving'* due to increasing climate suitability in temperate Box Ironbark forests and grassy eucalypt woodlands of Redbank, south-west Victoria (164e, Anne Hughes, President, St Arnaud Field Naturalist Club and covenanted landholder), southward movement of the *Eudynamys*

orientalis (Eastern Koel): *'The eastern koel was wholly absent from Bungendore and has only been prominent for the past 5 or so years.... Anecdotally it appears that the koels have penetrated further south and this has been attributed to climate change...drying and warming conditions'* (7c, Bungendore, south-east New South Wales, Anon.), and of *Ocyphaps lophotes* (Crested pigeon) and *Corcorax melanorhamphos* (White-winged choughs): *'20 years ago Crested pigeons were not seen south of Bendigo, today they are now common as far south as the coast.'* (66b, Winter Swamp, Cardigan, Victoria, Ray Draper, Central Highlands Environmental Consultancy), *'Warmer, drier conditions bringing crested pigeons and choughs south.'* (157a, Westmere, south-west Victoria, M. T. Casanova). Remaining cases were not explicit about the type of shift involved (e.g. noting only that the species had appeared where it previously had not been recorded). Other examples of perceived range shifts involved shifts at the ecological community level, discussed later under biome shifts.

While perceived drivers of species arrivals most commonly involved direct effects of warming or drying, intermediary steps were occasionally noted, including replacement of local species (e.g. *'Casuarina is now significantly depleted with many drier country trees establishing in the area* [Boondall Wetlands Conservation Reserve, south-east Queensland]....*Fig trees* [Ficus sp.] *are appearing where none were before'* (132c, Anon.), and dependence on arrival of plants providing habitat (*Manorina melanophrys*, Bell miner, arriving after spread of *Lantana camara*, Lantana, in turn purported to result from reduced frosts, 300b, observed at Bulga, New South Wales). Consequences of species arrivals were also described, including competitive effects of invasive plants (e.g. *'Freesias are...displacing native orchids'*, 16a, Aldgate, South Australia, Anon.), plant arrivals facilitating animal arrivals (see above), and animal arrivals disadvantaging local natives (e.g. *'Arrival of predatory bird species about 5 years ago: butcher birds, kookaburras which like warmer, dryer climate ... with consequence drastic reduction in all the smaller bird species (virtually 100% reduction)'*, 76a, Cygnet Wetlands, southern Tasmania, Anon.).

4 Communities to biomes

4.1 Communities and ecosystems

Changes other than those described in relation to the prior nine primary questions pertained mostly to community, ecosystem or biome level outcomes described in the climate change impact frameworks of Bellard et al. (2012) and Scheffers et al. (2016), representing some of the more complex ecological impacts of climate change that can be difficult to predict (Walther 2010). Here we discuss these higher-level changes under four main categories: impacts on interspecific and intertrophic interactions, community productivity, ecosystem structure and composition, and landscape-scale ecological processes.

Interspecific and intertrophic relationships. Direct effects of climate change on individuals, populations and species are expected in turn to impact (positively or negatively) on other species that they interact with in ecological communities (Parmesan 2006, Bellard et al. 2012). Text anecdotes elucidated over 50 cases of potentially climate change-induced changes to interspecific or intertrophic relationships, through descriptions of ecological cascades (Table J in S1 Table), a range of which have already been highlighted in earlier sections. Most involved a single cascade (i.e. a primary climate-change driven change leading to a secondary change), although 11 cases involved two cascades (three biological changes), and one case involved three cascades (four levels of biological change: warming and eutrophication-induced algal blooms leading to decline in lizards at

Lake Goegorup, Western Australia, with subsequent change to insect populations, in turn affecting birds, 129a).

The reported cascades implied changes to at least eight different types of ecological interactions (Table 7 main text), the most commonly-reported type involving plant-herbivore interactions (Table J in S1 Table). Changes to plant-herbivore interactions pertained to both invertebrate and vertebrate herbivores, and were initiated either through climate change-induced changes to plant health or abundance (e.g. climate-stressed *Eucalyptus camaldulensis* (River red gum) more susceptible to lerp (Family Psyllidae) infestations, leading to tree death (187a, Hamilton, south-west Victoria), and *Banksia robur* (Swamp banksia) dying in Wallum heath, leading to disappearance of small mammals, (326a, Cooloola Cove, Wide Bay-Burnett area, Queensland)), or by climate change-induced changes to invertebrate populations (e.g. longer weevil (Superfamily Curculionidaea) persistence or earlier lerp (Family Psyllidae) 'attack') resulting in greater plant damage. The next most commonly perceived changes in interspecific relationships were to host-pathogen interactions, typically involving reduced resilience of plant (e.g. eucalypts, banksias) or animal (e.g. *Phascolarctos cinereus,* Koala; *Pteropus poliocephalus*, Grey headed flying foxes; *Pteropus alecto*, Black flying fox; *Pteropus scapulatus*, Little red flying fox) hosts to pathogens due to climate stress; and changes in habitat (typically involving a change in vegetation structure or density) with flow on effects to birds or mammals, or sometimes to other plants (e.g. tree ferns), reptiles (lizards) or fish (trout) (Table J in S1 Table).

Changes to competitive interactions, predator-prey interactions and loss of synchronization were other occasionally reported types of change to interspecific relationships. The former typically involved suppression of local native plants by invading native or exotic plants (e.g. *Ligustrum sinense* (Small leafed privet) producing viable seed up to 3 months earlier in Upper Coopers Creek, north coast New South Wales, leading to increased competitiveness over native plants, 153a), but included two cases of native or exotic birds displacing native birds (e.g. *Artamus personatus* and *A. superciliosus* (Masked and White browed woodswallows) displacing *Artamus cyanopterus* (Dusky woodswallow) in the Lake Burrendong area, central west New South Wales, 161e). While not strictly reported as changes to interspecific relationships, a number of other species replacements were also reported (see section 'Ecosystem structure and composition', below), that may involve a competitive dynamic.

Perceived changes in predator-prey relationships included both primary climate-driven changes to predators resulting in impacts on prey (e.g. arrival of predatory bird species (*Cracticus* sp., Butcher bird; *Dacelo novaeguineae*, Laughing kookaburra) and subsequent drastic reduction or disappearance of a suite of native and exotic small birds (76a, Main Ridge, Melbourne, Victoria), and vice-versa (e.g. dry conditions causing decline in mosquitoes (Family Cuculidae) and other bugs, leading to starvation of frogs (Order Anura), 113e, Ewlyamartup, Western Australia). Five of the six reported cases of loss of synchronization involved changed flowering times with flow on effects to insects, birds and/or mammals, including a triple cascade involving altered timing of flowering leading to asynchrony between *Petaurus breviceps* (Sugar glider) prey and hatching of owl young (Order Strigiformes, 316b, Mullumbimby, north coast New South Wales, see also 3.2.4 'Animal mortality'). The sixth case involved changed fruiting times leading to longer food availability for *Strepera graculina* (Pied currawong) (see 3.2.6 'Animal phenology', 316b).

Less commonly-reported changes to interspecific relationships involved uncoupling of mutualisms and changed host-parasite interactions. Perceived uncoupling of mutualisms included potentially reduced pollination of *Caladenia amoena* (Charming spider-orchid) due to climate change-induced

decline in thynine wasp (Family Thynnidae) pollinators in Box-Ironbark forest of Victoria (32b, Plenty Gorge Park, Bundoora, Melbourne, Victoria), disappearance of *Westralunio carteri* (Freshwater mussel) in a Swan Coastal Plain stream due to loss of fish on which they depend for reproduction and dispersal (86a, Jane Brook, Western Australia), and two examples of decline in *Dicaeum hirundinaceum* (Mistletoebird) due to decline in Mistletoe (Order Santalales, 151g, 188d at Gunderlong in south-east Queensland, and Bendigo in north central Victoria, respectively). There was only one case of changed host-parasite interactions, involving a lengthened season for *Ixodes holocyclus* (Paralysis tick) due to warmer weather experienced at Logan in south-east Queensland, resulting in *Vulpes vulpes* (European fox) decline (87b).

Community productivity. Scheffers et al. (2016) considered changes in ecosystem productivity to be one of the most critical impacts of climate change on ecosystems, with the global trends to date resulting in a net increase in terrestrial plant growth. Respondents of our survey occasionally alluded to changes in plant growth rates or length of growing season in association with changing climate or weather conditions, implying changes in ecosystem (or sub-stratum) productivity (Table J in S1 Table). These included several cases suggesting reduced productivity, including *'poor growth of understorey species such as native grasses'* due to hotter, drier conditions (151e, Gunderlong, south-east Queensland, Dr J. Blok), *'less grass growth'* due to elevated growing season temperatures (161c, Lake Burrendong area, central west New South Wales, long term observations by Neville Mattick/Hargraves NSW), and reduced pasture productivity due to frosts in Logan, south-east Queensland (87a). The remaining six examples involved increasing productivity. These included increased algal blooms due to warming and eutrophication (129a) , *'more herbs and grasses and bushes' ('mostly bluebush* [Maireana *spp.*] *and salt bush* [Atriplex *spp.*] *varieties with a sprinkling of native fuschias* [Eremophila *spp.*] *and other things)...rather than hectares of bare ground'* potentially due to *'changes to the latitude of 'typical weather patterns''* in Forrest, south-east Western Australia (79a, Cathy Brown); *'Kikuyu* [Pennisetum clandestinum] *pasture* [in Kangaroo Valley, south coast New South Wales] *now growing all year round rather than being frosted off and unproductive in winter (May to November)'* (303a, Greg Thompson, Brogers Creek Landcare); *'extra growth of* Triodia *spp. and colonisation of more marginal habitats in wet years'* in far north Queensland (25a, Dan Kelman, Qld Herbarium); and *'More large rainfall events leading to more fuel in subsequent dry seasons'* (90a, Simpsons Gap National Park, Northern Territory, Ashley Sparrow). Respondents variously linked increases in productivity with changes in fire regimes, improved agricultural production and increases in fauna populations, whereas productivity declines were linked with reduced faunal health or abundance.

Ecosystem structure and composition. A range of anecdotes highlighted impacts of climate change on vegetation structure that typically resulted from cumulative effects of warming and/or drying on individual tree or shrub vigour or mortality. For example: in Gunderlong, south-east Queensland, a respondent noted *'The canopy of the taller trees is also much more open and foliage less dense, more leaves are falling throughout the year littering the forest floor'* (151f, Dr J. Blok). Another respondent from the Dandenong Ranges, Victoria, noted that *'Increase in summer heat and water stress is leading to a thinning of vegetation and loss of tree canopy'* (183a, David Jones). Violent storms (276a) and extensive hot fires (73b) were also seen as drivers of vegetation structural change. Outcomes of these changes included reduced habitat quality for fauna (e.g. for small birds (*Malurus lamberti* and *M. melanocephalus,* Variegated and Red-backed fairy-wrens; *Acanthiza pusilla*, Thornbill; *Sericornis* sp., Scrubwren; and robins (Family Petroicidae), 151f at Gunderlong, south-east Queensland), drying out of understorey (276a, Main Ridge, Melbourne, Victoria), shifts in

vegetation structural type (see below), and greater vulnerability to erosion (183a, Dandenong Ranges, Melbourne, Victoria).

Reported changes in species composition ranged from minor species or functional group replacements to indications of major shifts in vegetation types (Table J in S1 Table). The former included changes in algal composition due to warming and nutrient enrichment (129a, Goegorup Lake, Western Australia), declines in orchids and other flowering plants and increases in grasses in association with later autumn rains (140a, Lake Clifton, Western Australia), and increases in the diversity of weed species in response to summer rains (309b, Ellen Brook/Brockman River catchments, south-west Western Australia).

Shifts potentially indicative of substantial ecosystem change most commonly involved aridification of vegetation or impacts of sea-level rise. Emerging evidence for aridification included reports such as *'Bracken fern* [Pteridium esculentum] *now dominates the area that once was full of rainforest understorey, due to drying out of landscape'* (44a, Bunya Mountains National Park, south-east Queensland, Mark Cant, QPWS), *'drier forests are also seeing die back, reverting to a more woodland form'* (183a, Dandenong Ranges, Victoria, David Jones), *'decreased rainfall has created conditions suitable for dry rainforest plants and trees'* (132c, Boondall Wetlands Conservation Reserve, south-east Queensland, Anon.), and *'a gradual shift from lush subtropical riverine rainforest to a drier forest with fewer species and not a lot of understorey'* (316a, Mullumbimby, north coast New South Wales, Anon.). Aridification was also noted in wetlands, for example *'A small freshwater lake disappeared and is now covered with trees'* (perceived as a consequence of decreasing rainfall and increased ground water use, 246b, Neerabup, south-west Western Australia, Anon.), and similarly, *'whilst we have seen a reduction in* Melaleuca rhaphiophylla *in the wetland area, we have seen an increase in* Eucalyptus gomphocephala *which appears to be replacing the* Melaleuca *on the wetland boundaries'* (315a, Karnup, south-west Western Australia, Anon.).

Examples related to sea-level rise included several cases of encroachment of mangroves (27b, Princess Charlotte Bay, far north Queensland; 198b, Sandfly Beach, Kakadu National Park, Northern Territory), although one case involved mangrove die-off due to freshwater influx over several years following a 2011 flood in south-east Queensland (which may or may not have been related to climate change, 27a). Invasion of saline communities (including 'salt grasses') into woodlands, grasslands and wetlands was also reported over the past 60 years in Princess Charlotte Bay (27b). Only one example pertained to vegetation becoming more mesic, potentially in relation to increasing CO_2 and changed control burning regimes on the Atherton Tablelands, far north Queensland:

'While rainforest encroachment into eucalypt communities in wet tropical Australia is not new, in the last 20 years it has accelerated. While fire has traditionally been used to limit rainforest establishment, I suspect there is another factor involved - increased atmospheric CO_2. A suite of fire resistant, suckering rainforest species is now taking over a large proportion of the wet eucalypt communities' (100a, Anon.).

5 Landscapes

5.1 Landscape processes

While our study focused on biotic change, anecdotes also highlighted direct and indirect impacts of climate change on landscape scale processes, in particular, fire regimes, salinization and erosion (Table J in S1 Table). Examples of potentially climate-driven changes in fire regimes typically

pertained to one of two types: strengthening of rain-growth-fire cycles in arid Australia, and increase in large, intense fires in mountainous landscapes of south-eastern Australia. The former were commonly associated with increases in *Cenchrus ciliaris* (Buffel grass) and *Triodia* sp. (Spinifex) productivity in increasingly wet years, and resulting in structural vegetation change (25a, far north Queensland; 90a, Simpsons Gap National Park, Northern Territory; 211a, Alice Springs, Northern Territory: see earlier descriptions).

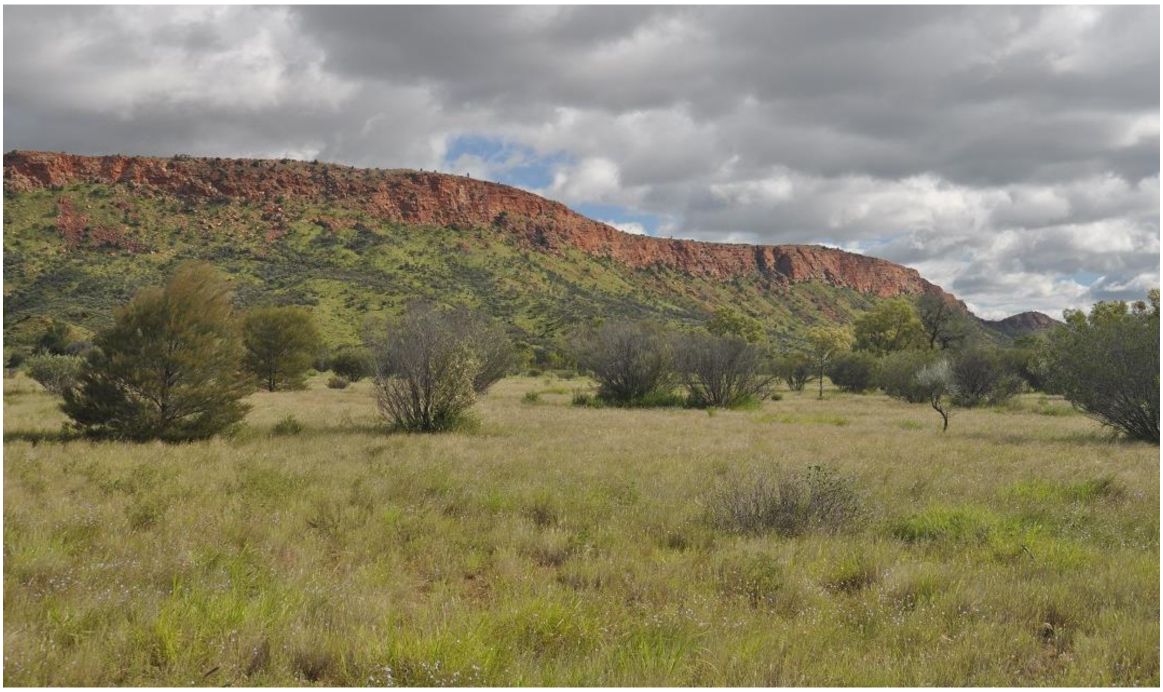

Plate 7 *Cenchrus ciliaris* (Buffel grass) after recent rain (March 2011), evident to the top of Heavytree Range near Alice Springs, Northern Territory. These increasing flushes in plant productivity across wider parts of the landscape are thought to be contributing to an intensifying rain-grass-fire-drought cycle in arid Australia (90a). Image credit: Ashley Sparrow.

It was not always clear whether respondents considered intense fires in mountainous landscapes to be a consequence of climate change, but terminology such as 'unprecedented' and 'catastrophic', and comments including *'in Tasmania dry lightning strikes were almost unknown'* (14b, Deborah Hunter, Mole Creek Caving Club), suggests this is likely or feasible (Table I in S1 Table). Indeed, a respondent working in *Eucalyptus delegatensis* (Alpine ash) forest of the Australian Alps noted *'I believe this is a climate change impact as there are many ecological indicators that this type of fire is unprecedented'* (186a, Jim Blackney). Drivers of these unprecedented intense fires were reported to include increased dry lightning incidence (especially in Tasmania, 14b, 91a), extremely warm and/or dry summer conditions (186a, 223b), and in one case, exacerbated by the large extent of forest logging (223b). These fires were usually interpreted as leading to unprecedented vegetation change or loss, including *'dry lightning over 2 days burned...thousands of hectares of climax rainforest...12,000 years of peat accumulation destroyed* [in north-west Tasmania] (14b, Deborah Hunter, Mole Creek Caving Club), *'decimation of all vegetation on the plateau'* (204b, Barrington Tops, New South Wales, a bushwalker of over 30 years experience), and decline in Alpine Ash forests, with *'litter burnt to mineral earth...lizards...gone...rivers...so warm the trout wouldn't bite*...[trees] *having difficulty regenerating'* (186a, Jim Blackney).

Additional examples of apparent changes in fire regime included escalating fire-invasion cycles after invasion by *Acacia longifolia* (Sallow wattle) into the Wimmera Region of south-west Victoria (73a),

suggesting indirect effects of climate change on fire in fragmented landscapes, via its effects on land use: *'The drier conditions have allowed broad acre cropping to be undertaken in areas that were once considered unviable due to water logging or poorer soil...we are witnessing a lot more burning off as a tool to handle crop residues and in the process paddock trees and roadside vegetation is being irreparably damaged'* (73c, Anon.).

Respondents occasionally noted increases in soil, stream or beach erosion in association with potential impacts of climate change on vegetation or fauna. This included soil erosion as a consequence of climate change impacts on vegetation cover, e.g. *'This overall decrease in bush density has made it more vulnerable to erosion'* (151f, Gunderlong, south-east Queensland, Dr J. Blok), *'more and longer dry periods reduce the amount of feed, bake the crust and result in erosion when rain occurs'* (306b, Moormbool, Central Highlands Victoria, Anon.), *'loss of understorey in bush areas...results in the following cascade: failure to hold moisture- flash floods, erosion, loss of seed bank, simplification of habitat'* (164f, Stoney Creek Nature Conservation Reserve/Kara Kara National Park, south-west Victoria, Anne Hughes, President, St Arnaud Field Naturalist Club and covenanted landholder). On the other hand, stream and beach erosion were mostly reported as potential drivers of biological impacts. In particular, stream erosion was reported where increased flooding was perceived, e.g. *'Major storm/flash flooding events in summer 2012 and 2013 gouged out the creek line with major soil erosion and loss of chains of ponds'* (239c at Tharwa, Australian Capital Territory, Anon.), leading to perceived consequences such as weed invasion or decline in waterway plant and animal diversity. Beach erosion was similarly noted to result in impacts on sea birds and dune vegetation (180b, 198a), but it was not clear whether respondents felt the erosion was due to sea-level rise or normal levels of beach erosion.

Finally, reports of impacts on landscape hydrology related largely to coastal examples of salinization in association with sea-level rise. These included direct contact of some vegetation with increasingly high levels of spring tides (198a), examples of mangrove invasion or retreat described above (27b, 117a, 198b), and salinization of soils, ground water, ponds and wetlands, affecting trees, birds and tadpoles (27a, 180a, b).

Literature Cited

Bellard, C., C. Bertelsmeier, P. Leadley, W. Thuiller, and F. Courchamp. 2012. Impacts of climate change on the future of biodiversity. Ecology Letters **15**:365-377.

Duke, N. C., J. M. Kovacs, A. D. Griffiths, L. Preece, D. J. E. Hill, P. van Oosterzee, J. Mackenzie, H. S. Morning, and D. Burrows. 2017. Large-scale dieback of mangroves in Australia. Marine and Freshwater Research **68**.

Gardner, J. L., T. Amano, P. R. Y. Backwell, K. Ikin, W. J. Sutherland, and A. Peters. 2014. Temporal patterns of avian body size reflect linear size responses to broadscale environmental change over the last 50 years. Journal of Avian Biology **45**:529-535.

Nicotra, A. B., O. K. Atkin, S. P. Bonser, A. M. Davidson, E. J. Finnegan, U. Mathesius, P. Poot, M. D. Purugganan, C. L. Richards, F. Valladares, and M. van Kleunen. 2010. Plant phenotypic plasticity in a changing climate. Trends in Plant Science **15**:684-692.

Parmesan, C. 2006. Ecological and evolutionary responses to recent climate change. Annual Review of Ecology Evolution and Systematics **37**:637-669.

Scheffers, B. R., L. De Meester, T. C. L. Bridge, A. A. Hoffmann, J. M. Pandolfi, R. T. Corlett, S. H. M. Butchart, P. Pearce-Kelly, K. M. Kovacs, D. Dudgeon, M. Pacifici, C. Rondinini, W. B. Foden, T. G. Martin, C. Mora, D. Bickford, and J. E. M. Watson. 2016. The broad footprint of climate change from genes to biomes to people. Science **354**.

Sgro, C. M., A. J. Lowe, and A. A. Hoffmann. 2011. Building evolutionary resilience for conserving biodiversity under climate change. Evolutionary Applications **4**:326-337.

Walther, G. R. 2010. Community and ecosystem responses to recent climate change. Philosophical Transactions of the Royal Society B-Biological Sciences **365**:2019-2024.
